# Synthesis of functionalized, 13-alkyl-substituted coralyne derivatives and investigation of their interactions with duplex and abasic site-containing DNA

**DOI:** 10.3762/bjoc.22.84

**Published:** 2026-07-13

**Authors:** Laurin Beckmann, Jason Lennard Kunze, Hannah Karola Strunk, Maurice Michel, Heiko Ihmels

**Affiliations:** 1 Department of Chemistry-Biology, and Research Center of Micro- and Nanochemistry and (Bio-)Technology (Cμ), University of Siegen, Adolf-Reichwein-Str. 2, 57068 Siegen, Germany; 2 Department of Oncology and Pathology, Karolinska Institutet Science for Life Laboratory, Alpha 5, Tomtebodavägen 23a, 171 65 Solna, Sweden; 3 Center for Molecular Medicine, Karolinska Institute and Karolinska Hospital, L8:02, Visionsgatan 18, 17176 Stockholm, Sweden

**Keywords:** APE1 inhibition, DNA binders, N-hetarenes, papaverine, protoberberines

## Abstract

With the goal to develop coralyne-based ligands for abasic site-containing DNA (AP-DNA), different synthetic routes towards the functionalization of coralyne were tested. In particular, the alkylation of the benzylic position in papaverine and subsequent cyclization by treatment with acetic anhydride in sulfuric acid led to the synthesis of a coralyne derivative with an alkoxyamine-functionalized linker attached at position C13. Firstly, it was demonstrated with a resembling model compound that the alkylation of the coralyne scaffold does not influence the DNA-binding properties. Furthermore, it was shown that the alkoxyamine function can be readily released from a Boc-protected precursor and that the corresponding coralyne–alkoxyamine conjugate can operate as a ligand for AP-DNA and as inhibitor of enzymatic repair of abasic sites.

## Introduction

DNA is a main target in the development of antitumor agents, because the inhibition or restriction of the essential functions of DNA has a strong influence on cell viability [1]. Therefore, the search for new compounds that selectively bind to DNA and thus significantly influence its biological activity is still an intensively investigated interdisciplinary research field [2–5]. In particular, one focus is on external ligands which form complexes with DNA by groove binding or intercalation and thus block the binding sites of the DNA and/or significantly change the helix structure at the binding site [2–5]. These structural changes may, in turn, suppress recognition processes between DNA and physiologically relevant enzymes, which are essential for important DNA-based cellular processes. For instance, genotoxic, antitumoral DNA ligands, such as topoisomerase inhibitors, operate according to this principle [6–7].

**Figure 1:**
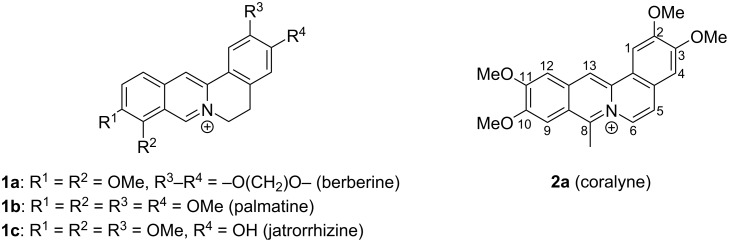
Structures of the representative DNA-binding protoberberine alkaloids **1a**–**c** and coralyne (**2a**).

In this context, several classes of polycyclic, nitrogen-containing hetarenes (N-hetarenes) have been shown to bind selectively and with high affinity to DNA [8–12], and, in some cases, to cause photoinduced DNA damage [13–15]. As a result, the synthesis and development of novel DNA-binding N-hetarenes still constitutes a cornerstone in current drug development and in the search for promising drug candidates. Along these lines, the protoberberines, such as, e.g., berberine (**1a**), palmatine (**1b**), or jatrorrhizine (**1c**) (Figure 1), may be considered as privileged structures because they provide a favorable combination of versatile and strong association with nucleic acids with pronounced DNA-targeted biological activity [10,16–17]. Particularly noteworthy within the series of known protoberberines is coralyne (**2a**). This synthetic dibenzo[*a,g*]quinolizinium derivative is structurally closely related to the alkaloids berberine (**1a**) and palmatine (**1b**) [18] and alike these protoberberines, coralyne (**2a**) binds to different nucleic acids [19–23]. For instance, it intercalates into double-stranded DNA, along with aggregation along the DNA backbone. Coralyne (**2a**) also binds to non-canonical DNA structures, such as G-quadruplex DNA (G4-DNA) and triplex DNA [24–27]. In addition, it is considered a highly potential drug candidate as it has significant biological activity [28–29]. To add to that, coralyne (**2a**) is a very efficient photosensitizer that induces DNA damage upon irradiation [30–34]. And this feature that can be utilized in photodynamic therapy (PDT) [35].

**Scheme 1:**
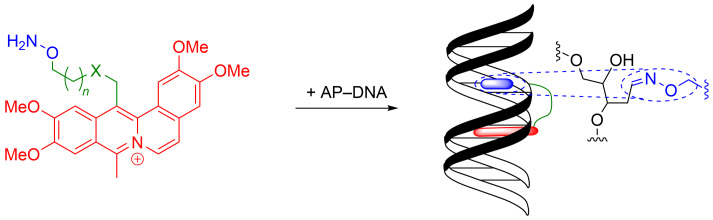
Sketch of the proposed combination of covalent oxime ether formation and DNA intercalation with a coralyne–alkoxyamine conjugate.

To further explore the high propensity of coralyne to bind to DNA and to induce DNA photodamage, we aimed at the further functionalization of this scaffold with additional DNA-binding units. Specifically, we proposed that the attachment of an (aminooxy)alkyl functionality may lead to a highly selective ligand for abasic site-containing DNA (AP-DNA) [36–37]. The latter is duplex DNA with apurinic/apyrimidinic (AP) sites, more generally named abasic sites (Scheme 1). Such AP sites play a key role in enzymatic DNA repair processes [38–39] and therefore, AP-DNA-binding ligands have the potential to be used as chemotherapeutic drugs [40]. In this context, the (aminooxy)alkyl group is an important feature because it allows the covalent attachment of the drug to the AP site through oxime formation with the aldehyde functionality of ring-opened ribose [37–38]. Thus, a coralyne–(aminooxy)alkyl conjugate might offer the combination of selective covalent attachment with intercalation of the coralyne unit (Scheme 1), thus providing a more efficient blocking of the AP site, along with a more efficient preliminary DNA binding of the ligand before covalent connection. Herein, we report on our general attempts to synthesize coralyne derivatives with additional substituents in position 13 as well as exemplary studies of their ability for DNA binding and enzyme inhibition.

## Results

**Scheme 2:**
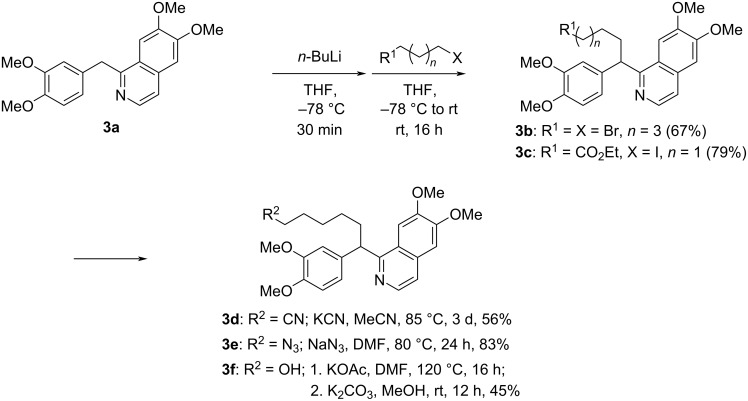
Synthesis of alkylated papaverine derivatives **3b**–**f**.

The synthesis of the coralyne derivatives started with papaverine (**3a**), as it is a readily available starting material and its transformation to coralyne (**2a**) is an established and reliable synthetic route [41–43]. Moreover, the alkylation of the benzylic position of papaverine (**3a**) is well described [44]. Hence, papaverine (**3a**) was deprotonated in the benzylic position by the reaction with *n*-BuLi, and the resulting anion was treated with 1,5-dibromopentane or ethyl 4-iodobutanoate [45] to give the alkylated papaverine derivatives **3b** and **3c** in 67% and 79% yield, respectively (Scheme 2). Subsequent nucleophilic substitution of **3b** with KCN furnished the nitrile-functionalized papaverine **3d** in 56% yield. However, attempts to hydrolyze the nitrile **3d** to the corresponding carboxylic acid failed both under mild and harsh alkaline conditions as well as under acidic conditions, as TLC and ^1^H NMR-spectroscopic analysis of the reaction mixtures showed either no conversion or decomposition. In another approach, the reaction of the bromopentyl-substituted papaverine **3b** with NaN_3_ or potassium acetate yielded the azide **3e** in 83% and the ester (Scheme 2). The direct alkaline hydrolysis of the latter furnished the hydroxyalkyl-substituted product **3f** in 45% yield (over the two reaction steps). All novel compounds were identified and characterized by ^1^H and ^13^C NMR spectroscopy (2D, COSY, HSQC, HMBC), mass spectrometry, and elemental analysis (if necessary as hydrochloride salts). Additionally, IR-spectroscopic analysis of the azide **3e** revealed characteristic bands for the azide group at 2094 cm^−1^.

**Scheme 3:**
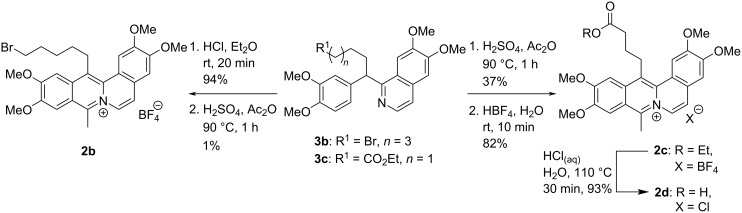
Synthesis of 13-alkylated coralyne derivatives **2b**–**d**.

The papaverine derivatives **3b** and **3e** were firstly transformed to their corresponding hydrochloride salts, which were subsequently treated with acetic anhydride in the presence of conc. H_2_SO_4_ at 90 °C (Scheme 3). Under these conditions, the azide **3e** decomposed, as indicated by ^1^H NMR-spectroscopic analysis of the reaction mixture, whereas the bromoalkyl-substituted papaverine **3b** gave the coralyne derivative **2b**, albeit in a very low yield of 1%. The product **2b** was identified by NMR spectroscopy, mass spectrometry, and absorption spectroscopy. The papaverine **3c** was directly made to react with acetic anhydride and conc. H_2_SO_4_ to give the corresponding coralyne **2c** as sulfoacetate salt in 37% yield. After ion metathesis by treatment with HBF_4_ the resulting tetrafluoroborate salt **2c** was submitted to acid-catalyzed hydrolysis to give the coralyne carboxylic acid derivative **2d** in 93% yield (Scheme 3). The novel compounds **2c** and **2d** were identified and characterized by NMR spectroscopy (^1^H, ^13^C, COSY, HSQC, HMBC), mass spectrometry, and elemental analysis.

**Scheme 4:**
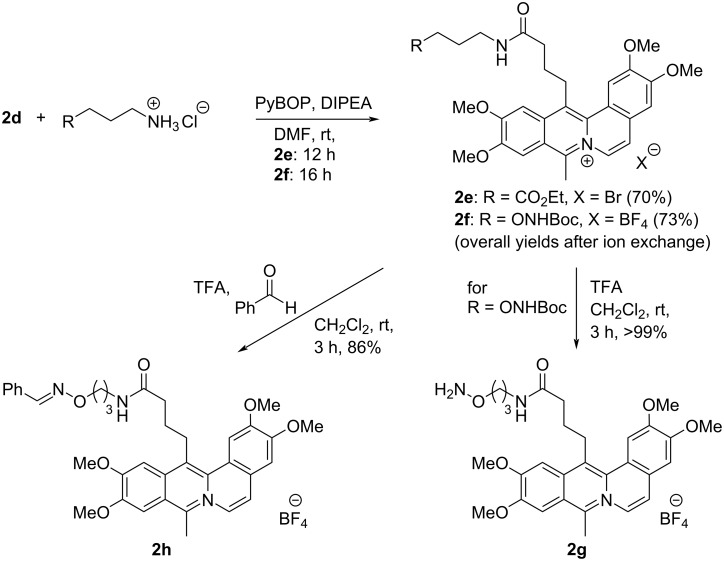
Synthesis of coralyne derivatives **2e**–**h** (Boc: *tert*-butyloxycarbonyl; DIPEA: *N*,*N*-diisopropylethylamine; PyBOB: benzotriazol-1-yl-oxytripyrrolidinophosphonium hexafluorophosphate; TFA: trifluoroacetic acid).

For the synthesis of a reference compound without alkoxyamine unit, the carboxylic acid unit of coralyne **2d** was esterified by the reaction with ethyl 4-aminobutanoate hydrochloride [46] and PyBOP as coupling reagent (Scheme 4). The structure of the resulting coralyne was confirmed by NMR spectroscopy (^1^H, ^13^C, ^19^F) and mass spectrometry, but the analysis also revealed a mixture of counter anions PF_6_^−^ and Cl^−^. Therefore, the product was converted into its bromide salt by ion exchange chromatography to give coralyne **2e** in an overall yield of 70%. Likewise, coralyne **2d** was functionalized with an aminooxyalkyl linker [37] by PyBOP-assisted coupling and ion metathesis to give the coralyne derivative **2f** (Scheme 4), which was identified and characterized by 1D and 2D NMR spectroscopy (^1^H, ^13^C, ^19^F), high-resolution mass spectrometry, and elemental analysis.

The Boc protection group in coralyne **2f** was removed by the reaction with trifluoroacetic acid (TFA) in CH_2_Cl_2_ to give the aminooxy-functionalized coralyne derivative **2g** in quantitative yield (Scheme 4). To examine exemplarily whether the latter product can be subsequently employed for oxime ether formation, it was also generated in the presence of benzaldehyde, which furnished the corresponding oxime–coralyne conjugate **2h** in 86% yield. The products **2g** and **2h** were identified by NMR-spectroscopic analysis and mass spectrometry.

### DNA-binding properties

**Figure 2:**
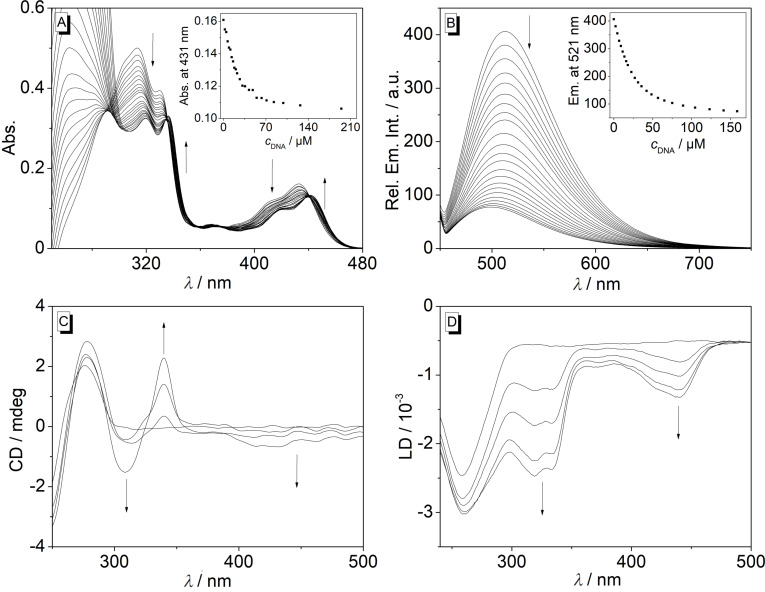
Photometric (A) and fluorimetric (B) titration of ct DNA to **2e** (*c* = 10 µM) (B) in BPE buffer (*c*_Na+_ = 16 mM, pH 7.0; with 10% v/v DMSO). Inset: plot of absorption (A) or emission (B) versus *c*_DNA_. CD (C) and LD (D) spectra of **2e** (*c* = 10 µM) in the presence of ct DNA in BPE buffer (*c*_Na+_ = 16 mM, pH 7.0; with 10% v/v DMSO) at LDR = 0.0–2.0. Red: DNA in the absence of ligand, blue: LDR = 2.0. The arrows indicate the development of the bands during DNA titration (absorption, emission), or the development of bands with increasing LDR (CD, LD).

To examine whether the DNA-binding properties of the coralyne unit are maintained even after attachment of the amidoalkyl functionality, the interactions of the coralyne **2e** with calf thymus DNA (ct DNA) were examined with photometric and fluorimetric titrations as well as with circular dichroism (CD) and linear dichroism (LD) spectroscopy (Figure 2). The experiments were carried out in aqueous buffer solutions with 10% of DMSO, because of the low water-solubility of **2e**.

Upon addition of DNA, the absorption of the long-wavelength band (λ_max_ = 433 nm) of coralyne derivative **2e** decreased with a significant red shift of the absorption maximum. Moreover, two isosbestic points at 335 nm and 440 nm formed initially during DNA titration, but they faded with progressing titration (Figure 2A). The resulting binding isotherm from the complete titration data was used to determine the binding constant of the ligand as *K*_b_ = 1.4 × 10^5^ M^−1^ [47]. In aqueous buffer solution (with 10% DMSO), the coralyne derivative **2e** showed a strong fluorescence with a maximum at 509 nm and a fluorescence quantum yield of Φ_fl_ = 0.40. The titration of **2e** with ct DNA led to strong quenching of the fluorescence by up to 82% at saturation (Figure 2B). CD-spectroscopic analysis of the ligand–DNA complexes revealed slight pertubation of the characteristic band of the DNA at 285 nm. At the same time, induced CD (ICD) bands were formed in the range of the ligand absorption (>300 nm), namely a relatively strong bisignate ICD band at 300–360 nm and a weak negative band at 400–460 nm (Figure 2C). Further analysis of the DNA-bound ligand by LD spectroscopy showed a negative LD band at 300–350 nm and a weaker negative one at 400–460 nm, which both increased with increasing ligand-to-DNA ratio (LDR) (Figure 2D).

### Interaction of **2g** with abasic site-containing DNA

**Figure 3:**
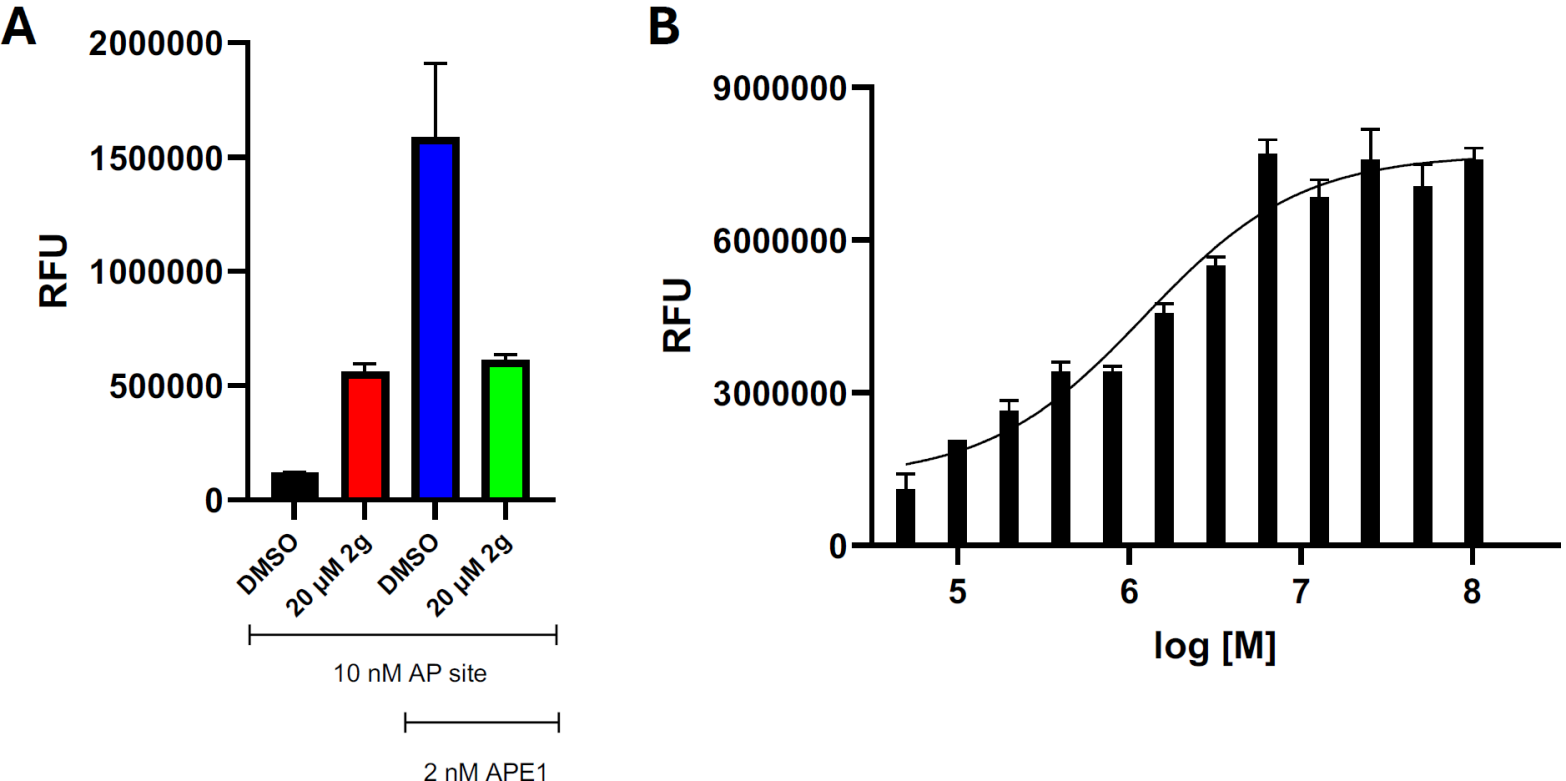
A: Influence of **2g** on APE1-mediated cleavage of AP-site DNA. The AP–DNA (10 nM) was incubated with **2g** (20 µM) prior to addition of APE1 (2 nM). APE1-mediated cleavage is indicated by increasing fluorescence intensity (RFU) of the reporter dye. B: Determination of the IC_50_ of **2g** for inhibition of APE1-mediated AP-site processing. AP–DNA was preincubated with a two-fold dilution series of **2g**, followed by addition of APE1. The fluorescence response (RFU) after completion of the reaction (≈1 h) was plotted as a function of ligand concentration (log[M]) . Nonlinear regression analysis (solid line): IC_50_ = 0.78 µM.

To investigate whether the aminooxy-functionalized coralyne derivative **2g** interacts with AP sites in DNA and whether such binding leads to DNA cleavage or inhibition of AP-site processing, a series of experiments was performed with a fluorescence-based AP-site cleavage assay (Figure 3A) [48]. In this assay, cleavage of the AP site results in separation of a fluorophore–quencher pair, leading to an increase in the fluorescence signal [49]. AP sites were generated enzymatically by treatment of uracil-containing DNA with uracil–DNA glycosylase (UNG2), producing the corresponding AP–DNA substrate. The AP–DNA (10 nM) was then incubated with compound **2g** at 20 µM to determine whether the ligand induces strand cleavage. The reaction was monitored fluorimetrically with an excitation wavelength of 485 nm and an emission wavelength of 535 nm. At 20 µM, an increased background signal was observed, likely due to autofluorescence of compound **2g**, which partially overlapped with the assay signal (Figure 3A). Importantly, under these conditions no significant increase in fluorescence was detected, demonstrating that compound **2g** did not induce detectable DNA strand cleavage. To assess whether the ligand binds to AP sites and interferes with their enzymatic processing, the activity of apurinic/apyrimidinic endonuclease 1 (APE1) was examined in the presence of the ligand. In this assay, cleavage of the AP site by APE1 separates the fluorophore from the quencher, which results in a characteristic fluorescence signal. If compound **2g** binds to the AP site through its aminooxy functionality, access of APE1 to the lesion site would be expected to be hindered, and the fluorescence remains quenched. Accordingly, a repetition of the above experiment was performed, in which the two conditions including either AP sites or an AP site incubated with 20 µM of **2g** were further incubated with APE1 (Figure 3A). In the absence of ligand, rapid cleavage by APE1 produced the expected fluorescence increase. In contrast, in the presence of **2g** the signal was reduced to the level caused by **2g** alone.

To quantify this inhibitory effect, the IC_50_ value of compound **2g** toward APE1-mediated AP-site cleavage was determined with a two-fold serial dilution series (Figure 3B). The AP–DNA substrate (10 nM) was preincubated with ligand concentrations starting at 20 µM, followed by 10 µM, 5 µM, and lower concentrations. After a 10–15 min preincubation, APE1 was added to initiate the reaction, and fluorescence signals were recorded after completion of the assay. Analysis of the resulting dose–response curve enabled determination of the concentration of compound **2g** required to inhibit APE1-mediated cleavage by 50% (IC_50_ = 0.78 µM). Based on these results, a ligand concentration of 20 µM should be selected for future experiments, as it resulted in near-complete blocking of the AP site while maintaining acceptable fluorescence background levels. Taken together, these experiments indicate that compound **2g** interacts with AP sites and competitively inhibits APE1-mediated cleavage, which is consistent with binding of the ligand to the AP site.

## Discussion

The spectrometric titrations of coralyne derivative **2e** with ct DNA clearly indicate the complex formation. In particular, the hypochromism and the bathochromic shift of the absorption maxima, the fluorescence quenching, and the formation of clear ICD signals upon addition of DNA are characteristic features of a DNA-binding compound [49–51]. In addition, most of the titration spectra resemble the ones of coralyne (**2a**) under similar conditions, and the binding constant (*K*_b_ = 1.4 × 10^5^ M^−1^) is also in the same range as the one of the parent compound **2a** (*K*_b_ = 1.0 × 10^5^ M^−1^) [52–53]. It should be noted, however, that the fading isosbestic points during the photometric titration indicate two different binding modes, and accordingly, the calculated binding constants from the complete titration data only reflect the average binding constant of the combination of these modes. In this context, the LD-spectroscopic analysis of the ligand–DNA complex unambiguously indicated the intercalation of the coralyne unit of **2e** into the DNA by the characteristic negative LD band in the absorption range of the ligand [54–55]. At the same time, the relatively strong bisignate ICD signal is usually caused by exciton coupling [56] and thus suggests that the ligand also forms aggregates along the backbone, as has been shown for the parent coralyne [20,56].

It may be considered an inconvenience of coralyne and its derivatives that they usually exhibit a relatively low water solubility, as indicated by the required addition of DMSO to achieve reasonably high concentrations for spectroscopic analysis of compound **2e**. However, at least in the case of coralyne (**2a**), it has been shown that this property does not severley limit its bioavailability and cell permeability, and thus its bioactivity in real samples [29–30]. Although systematic tests along these lines have not been made with compound **2e**, it may be assumed that, because of its similarity with **2a**, it might also have sufficient, albeit very low, solubility in biological media.

The fluorimetric analysis of APE1-mediated DNA cleavage (Figure 3) indicated that the aminooxy-functionalized coralyne derivative **2g** binds to AP site-containing DNA, as the observed effect is characteristic of AP–DNA ligands. Moreover, this result is in agreement with the control experiment that demonstrated the general ability of compound **2g** to form oxime ethers with aldehydes (Scheme 4). As a result, this covalent attachment interferes with the enzymatic processing of these lesions by APE1. The observed concentration-dependent suppression of APE1-mediated cleavage, together with the determined IC_50_ value, suggest that the ligand effectively occupies the AP site, most likely through a combination of oxime formation between the aminooxy linker and the aldehyde functionality of the ring-opened ribose, along with intercalation of the coralyne unit into the DNA duplex (see above). Such a dual interaction mode would be expected to stabilize the ligand–DNA complex and sterically hinder access of repair enzymes to the lesion site [41,57]. With the available data, however, insertion of the coralyne part of **2g** in the AP site cannot be excluded. Nevertheless, as this is only a dynamic non-covalent interaction, the competing covalent attachment through oxime formation will prevail.

## Conclusion

In summary, several approaches towards the functionalization of the coralyne core were tested starting from the alkylation of the benzylic position in papaverine and subsequent coralyne formation by a Pictet–Gams-type cyclization. One route eventually enabled the synthesis of conjugate **2g** with an attached alkoxyamine-functionalized linker, that is well known to bind to the ribose residues in abasic sites of damaged DNA [37–38,58]. In first control experiments, it was demonstrated that, indeed, the alkoxyamine can be released readily from a Boc-protected precursor and directly intercepted by reaction with an aldehyde. Complementary, it was shown with a resembling model compound, **2e**, that the alkylation of the coralyne scaffold does not influence the DNA-binding properties. Finally, biological tests unambiguously indicated that the alkoxyamine-functionalized coralyne derivative **2g** can act as a targeted AP-site ligand and inhibitor of APE1-mediated repair of AP sites.

Taken together these results provide a blue-print for the development of selective coralyne-based AP–DNA ligands. Even though only one example was made available, so far, the approach may be easily varied to obtain different linker lengths and linking units, so that ligands with optimized binding properties towards target AP–DNA should be available with this route.

## Experimental

### Equipment

NMR spectra: Jeol JNM-ECZR (^1^H: 500 MHz, ^13^C: 125 MHz, ^19^F: 470 MHz); Varian VNMR-S 600 (^1^H: 600 MHz, ^13^C: 150 MHz); at 25 °C. The chemical shifts (δ) are given in ppm and are referenced relative to tetramethyl silane (TMS, δ = 0.00 ppm), hexafluorobenzene (C_6_F_6_ δ_F_ = -164.9 ppm), or residual solvent signals (DMSO-*d*_6_: δ_H_ = 2.50 ppm, δ_C_ = 39.52 ppm); MestReNova 12.0.4-22023. MS (ESI): Finnigan LCQ Deca (*U* = 6 kV, working gas: argon, capillary temperature: 200 °C, auxiliary gas: nitrogen); analysis with FreeStyle 1.3 SP2. HRMS (ESI): Thermo Fischer Exactive (*U* = 2.7–3.5 kV, sheath gas: argon, capillary temperature 300 °C). Elemental analysis: HEKAtech EUROEA combustion analyzer (in-house). Melting points: Büchi 545 (Büchi, Flawil, CH); uncorrected. Absorption spectra: Varian Cary 100; quartz glass cuvettes (*d* = 10 mm). Emission spectra: Varian Cary Eclipse; (*d* = 10 mm). CD and LD spectra: applied photophysics chirascan spectropolarimeter; quartz glass cuvettes (*d* = 10 mm). Spectra processing: OriginPro (8.5.1G SR2); implemented smoothing function “adjacent averaging” with factor of 10 (absorption, CD and LD) or 20 (emission).

### Materials

Alfa Aesar GmbH + Co. KG (Kandel, DE): NaI (99%); BLD Pharmatech Ltd. (Shanghai, CHN): benzotriazol-1-yl-oxytripyrrolidinophosphonium hexafluorophosphate (PyBOP, 98%); Carl Roth GmbH + Co. KG (Karlsruhe, DE): *N*,*N*-diisopropylethylamine (DIPEA, 99%), trifluoroacetic acid (TFA, 99%); Fermentas (Vilnius, LTU): pBR322; Fluka (Darmstadt, DE): 4-aminobutyric acid (98%); KMF Laborchemie-Handels GmbH (Köln, DE): benzaldehyde (99%); Merck KGaA (Darmstadt DE): ct DNA (type 1); Sigma-Aldrich (St. Louis, US): 4-bromobutanoate (95%); *n*-BuLi (2.50 M in hexane); Tokio Chemical Industries Co., Ltd. (Tokio, JPN): papaverine hydrochloride (98%). Column chromatography: silica gel (60 M, 40–63 µm, 230–400 mesh ASTM, Macherey-Nagel GmBH & Co. KG).

Ethyl 4-iodobutanoate [46] and 1,1-dimethylethyl *N*-(3-aminopropoxy)carbamate hydrochloride [37] were synthesized according to literature protocols. Papaverine (**3a**) was obtained by neutralization of papaverine hydrochloride [58]. Benzaldehyde was distilled under reduced pressure prior to use. Other reagents and other solvents were used without further purification.

The buffer solutions were prepared from biochemistry-grade chemicals and E-Pure^®^ water (18 MΩ cm). They were filtered through a membrane filter (pore size 0.45 μM; Carl Roth GmbH, Karlsruhe) prior to use and stored at 4 °C. BPE buffer: *c*(Na_2_HPO_4_) = 6.0 mM, *c*(NaH_2_PO4) = 2.0 mM, *c*(Na_2_EDTA) = 1.0 mM, pH 7.0. TBE buffer (10×): *c*(tris(hydroxymethyl)aminomethane) = 89.0 mM, *c*(B(OH)_3_) = 89.0 mM, *c*(Na_2_EDTA) = 1.0 mM, pH 8.0; I_1/2_ buffer: *c*(KH_2_HPO_4_) = 5.0 mM, *c*(NaCl) = 50.0 mM, pH 7.4.

The ct DNA (approximately 2 mg/mL) was dissolved in BPE buffer and stored at 4 °C for at least 24 h. The solution was filtered through a membrane filter before use (pore size 0.45 μM; Carl Roth GmbH, Karlsruhe). The concentration (in base pairs, bp) was determined photometrically (λ_max_ = 260 nm, ε = 12824 cm^−1^ M^−1^) [25].

Oligonucleotides for biochemical assay: Complementary strands end-labeled with carboxyfluorescein (FAM) and 4-((4-(dimethylamino)phenyl)azo)benzene (DAB) were ordered from ATD BIO. Sequences were 5′-FAM-TCTG CCA XCA CTG CGT CGA CCT G-3′ and 5′-CAG GTC GAC GCA GTG YTG GCA GT-DAB-3′, where X is a lesion such as uracil, thymine glycol, 8-oxoA, AP-site analogue, inosine, hydroxymethyl cytosine and Y is the corresponding required complementary base.

Proteins: Enzymes (UNG, APE1, NEIL1, NTHL1, NEIL3, OGG1) and mutants were produced as reported previously [59].

### General methods

The reaction mixtures were stirred with a magnetic stirrer equipped with a stirring bar. The average room temperature (rt) was 22–24 °C. If not stated otherwise, operations were conducted at room temperature. Temperatures given in the experimental procedures refer to the medium that surrounds the reaction vessel. Solvents were removed at reduced pressure with a rotary evaporator. DMF was distilled from CaH_2_ and stored with added molecular sieves (4 Å). THF was distilled from sodium. DIPEA was stored with added molecular sieves (3 Å).

For photometric, fluorimetric, and polarimetric DNA titrations, stock solutions (*c* = 1.00 mM) were prepared in MeOH and stored at 4 °C. From this stock solution, a solution of the ligand (*c* = 10 µM) in BPE buffer containing 10% (v/v) of DMSO was prepared. The titrations were performed with a sample volume of *V*_sample_ = 1.0 mL. To avoid dilution effects during the titrations, a corresponding amount of the ligand was added to the DNA solution. A spectrum of the ligand was recorded prior to the titration. After each addition of DNA solution, the measurement was conducted after an equilibration time of 3 min. The concentration of the DNA was increased, until 20 equiv of DNA (bp) were added, or until the DNA precipitated from the solution. Absorption spectra were recorded from 250 to 600 nm at a rate of 120 nm min^−1^ at 20 °C. Emission spectra were recorded form 440 nm to 880 nm at a rate of 120 nm min^−1^ at 20 °C with a detector voltage of 550 V. Fluorescence quantum yield of **2e** was determined according to published protocol relative to coumarin 153 (in EtOH) [60] with λ_ex_ = 421 nm.

The data from photometric DNA titration of **2e** were analyzed in a modified Scatchard plot, that is, a plot of the saturation factor, SF, versus the DNA concentration. The binding constant was determined from fitting of the experimental data to the theoretical model [48].

For CD and LD-spectroscopic analysis, a stock solution of DNA in BPE buffer with a concentration of 200 µM was prepared. The ligand solution was taken directly from a stock solution in MeOH and the solvent was removed with a vigorous N_2_ gas stream. Samples were prepared in BPE buffer with constant DNA concentration (10 µM) and increasing ligand concentrations. The CD and LD spectra were recorded with a measuring speed of 1.0 nm s^−1^ at 20 °C in a cuvette with 10 mm (CD) path length, or a rotating cuvette (LD) with 1 mm path length. In LD experiments, the shear gradient was 1200 s^−1^.

The fluorimetric biochemical assay with AP-containing DNA was conducted with purified enzymes (see Materials) according to literature [60] and the "EUbOPEN" protocols [61] and adapted where mentioned. Assay progression on 10 nM substrate was monitored in a kinetic mode, and a time-resolved curve was obtained for each condition and concentration in triplicates except where stated otherwise. A standard fluorescence plate reader with λ_ex_ = 485 nm and λ_fl_ = 535 nm was used.

## Supporting Information

File 1 Complete description of synthesis and compound characterization with complete set of NMR spectra (Figures S1–S65).

## Data Availability

All data that supports the findings of this study is available in the published article and/or the supporting information of this article.
