# Supplementary material for: Synthesis of functionalized, 13-alkyl-substituted coralyne derivatives and investigation of their interactions with duplex and abasic site-containing DNA
Source: Beilstein J Org Chem. 2026 Jul 13;22:1057–66. doi: 10.3762/bjoc.22.84 (PMC13382958; doi:10.3762/bjoc.22.84)
Supplement: File 1 — Complete description of synthesis and compound characterization with complete set of NMR spectra (Figures S1–S65). [file Beilstein_J_Org_Chem-22-1057-s001.pdf]

BEILSTEIN JOURNAL OF ORGANIC CHEMISTRY

Supporting Information

for

Synthesis of functionalized, 13-alkyl-substituted coralyne derivatives and investigation of their interactions with duplex and abasic site-containing DNA

Laurin Beckmann, Jason Lennard Kunze, Hannah Karola Strunk, Maurice Michel and Heiko Ihmels

Beilstein J. Org. Chem. **2026,** *22,* 1057–1066. doi:10.3762/bjoc.22.84

Complete description of synthesis and compound characterization with complete set of NMR spectra (Figures S1–S65)

License and Terms: This is a supporting information file under the terms of the Creative Commons Attribution License (https://creativecommons.org/licenses/by/4.0). Please note that the reuse, redistribution and reproduction in particular requires that the author(s) and source are credited and that individual graphics may be subject to special legal provisions.
The license is subject to the *Beilstein Journal of Organic Chemistry* terms and conditions: (https://www.beilstein-journals.org/bjoc/terms)

Table of contents

1. Synthesis S2
2. NMR spectra S12

1. Synthesis

1-(6-Bromo-1-(3,4-dimethoxyphenyl)hexyl)-6,7-dimethoxyisoquinoline (**3b**)

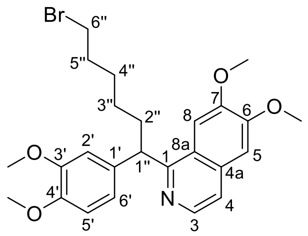

A solution of *n*-BuLi (2.5 M, in hexane, 4.3 mL, 11 mmol) was added dropwise to a solution of papaverine (**3a**, 3.00 g, 8.85 mmol) in anhydrous THF (100 mL) at −78 °C under Ar atmosphere. Stirring was continued for 30 min at −78 °C. Then, 1,5-dibromopentane (2.4 g, 1.4 mL, 11 mmol) was added dropwise at −78 °C, and the resulting reaction mixture was stirred for 1 h at −78 °C followed by 1 h at rt. Water (90 mL) and CH_2Cl_2 (200 mL) were added, the layers were separated, and the aqueous layer was extracted with CH_2Cl_2 (3 × 100 mL). The combined organic layers were washed with brine (1 × 200 mL), dried with Na_2SO_4 and filtered. The solvent was removed under reduced pressure and the crude was purified by column chromatography (SiO_2, neutral, CH_2Cl_2/MeOH 99:1, R_f = 0.4). The resulting colorless oil was washed with *n*-pentane (1 × 20 mL) to give **3b** as a colorless solid (2.91 g, 5.96 mmol, 67%); mp 101–102 °C. – ^{1}H NMR (500 MHz, $CDCl_3$): δ = 1.27–1.42 (m, 2H, 3''-H), 1.44–1.60 (m, 2H, 4''-H), 1.85 (quint, ^3J = 7.0 Hz, 2H, 5''-H), 2.13–2.22 (m, 1H, 2''-H), 2.41–2.51 (m, 1H, 2''-H), 3.37 (t, ^3J = 7.0 Hz, 2H, 6''-H), 3.77 (s, 3H, 3'-OMe), 3.81 (s, 3H, 4'-OMe), 3.95 (s, 3H, 7-OMe), 3.98 (s, 3H, 6-OMe), 4.60 (t, ^3J = 7.3 Hz, 1H, 1''-H), 6.76 (d, ^3J = 8.2 Hz, 1H, 5'-H), 6.87 (d, ^3J = 2.0 Hz, 1H, 2'-H), 6.92 (dd, ^3J = 8.2, ^4J = 2.0 Hz, 1H, 6'-H), 7.05 (s, 1H, 5-H), 7.38 (d, ^3J = 5.7 Hz, 1H 4-H), 7.43 (s, 1H, 8-H), 8.44 (d, ^3J = 5.7 Hz, 1H, 3-H). – ^{13}C NMR (125 MHz, $CDCl_3$): δ = 27.3 (C11), 28.3 (C12), 32.7 (C13), 34.1 (C14), 35.5 (C10), 48.9 (C9), 55.9 (C15, C16, C17, C18), 103.8 (C5), 105.5 (C8), 111.0 (C6´), 118.4 (C4), 120.2 (C5´), 123.0 (C8a), 133.4 (C4a), 137.2 (C1´), 140.8 (C3), 147.6 (C3´), 149.2 (C4´), 149.8 (C6), 152.3 (C7), 159.9 (C1). – El. Anal. for $C_{25}H_{30}BrNO_4$: calcd.(%): C 61.48, H 6.19, N 2.87; found (%): C 61.47, H 6.17, N 2.65.

1-(6-Bromo-1-(3,4-dimethoxyphenyl)hexyl)-6,7-dimethoxyisoquinoline hydrochloride (**3b-HCl**)

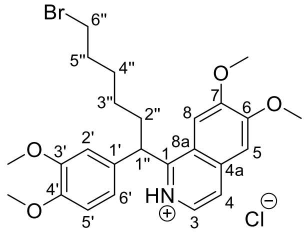

A solution of HCl (1.0 M, 2 mmol) in Et_2O (2 mL) was added to a stirred solution of **3b** (500 mg, 1.02 mmol) in Et_2O (120 mL), and the resulting suspension was stirred for 20 min at rt. The precipitate was filtered off and washed with *n*-pentane (2 × 10 mL) and Et_2O (2 × 10 mL) to give to product as colorless solid (502 mg, 956 µmol, 94%), mp 110–111 °C. – ^{1}H NMR (500 MHz, DMSO-d_6): δ = 1.30 (quint, ^3J = 7.6 Hz, 2H, 3''-H), 1.47 (hept, ^3J = 6.7 Hz, 2H, 4''-H), 1.74 (d quint, ^3J = 41 Hz, 4J = 7.0 Hz, 2H, 5''-H), 2.40–2.48 (m, 1H, 2''-H), 2.53–2.59 (m, 1H, 2''-H), 3.49 (t, ^3J = 7.0 Hz, 2H, 6''-H), 3.67 (s, 3H, 3'-OMe), 3.75 (s, 3H, 4'-OMe), 4.02 (s, 3H, 7-OMe), 4.05 (s, 3H, 6-OMe), 5.36 (t, ^3J = 7.4 Hz, 1H, 1''-H), 6.87 (d, ^3J = 8.2 Hz, 1H, 5'-H), 7.06 (dd, ^3J = 8.2, ^4J = 1.7 Hz, 1H, 6'-H), 7.33 (d, ^3J = 1.7 Hz, 1H, 2'-H), 7.74 (s, 1H, 5-H), 7.99 (s, 1H, 8-H), 8.13 (d, ^3J = 6.5 Hz, 1H, 4-H), 8.40 (d, ^3J = 6.5 Hz, 1H, 3-H), 15.44 (br s, 1H, 2-H).

1''-(Ethoxy-4''-carbonylpropyl)-1-(3,4-dimethoxybenzyl)-6,7-dimethoxyisoquinoline (**3c**)

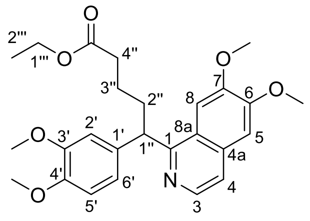

A solution of *n*-BuLi in hexane (2.50 M, 3.90 mL, 9.75 mmol) was added dropwise to a solution of papaverine (**3a**) (3.00 g, 8.85 mmol) in anhydrous THF (150 mL) at −78 °C under an Ar atmosphere, and stirring was continued for 30 min at −78 °C. The reaction mixture was added dropwise to a solution of ethyl 4-iodobutanoate (2.35 g, 2.40 mL, 9.72 mmol) in anhydrous THF (100 mL) at −78 °C. The mixture was allowed to slowly warm to rt, and stirring was continued for 16 h at rt. CH_2Cl_2 (300 mL) and water (200 mL) were added, the layers were separated and the aqueous layer was extracted with CH_2Cl_2 (2 × 100 mL). The combined organic layers were washed with water (1 × 300 mL) and brine (1 × 300 mL), dried with Na_2SO_4 and filtered. The solvent was removed under reduced pressure, and the residue was purified by column chromatography (SiO_2, neutral, EtOAc/Et_3N 99.5:0.5, R_f = 0.62) to give the product **3c** as a yellow oil (3.16 g, 6.96 mmol, 79%). – ^{1}H NMR (500 MHz, $CDCl_3$): δ = 1.22 (t, ^{3}J = 7.1 Hz, 3H, 2'''-H), 1.58–1.76 (m, 2H, 3''-H), 2.16–2.25 (m, 1H, 2''-H), 2.30–2.42 (m, 2H, 4''-H), 2.42–2.52 (m, 1H, 2''-H), 3.76 (s, 3H, 3'-OMe), 3.80 (s, 4'-OMe), 3.95 (s, 3H, 7-OMe), 3.98 (s, 3H, 6-OMe), 4.09 (q, ^{3}J = 7.1 Hz, 2H, 1'''-H), 4.62 (t, ^{3}J = 7.3 Hz, 1H, 1''-H), 6.75 (d, ^{3}J = 8.2 Hz, 1H, 5'-H), 6.86 (d, ^{3}J = 2.0 Hz, 1H, 2'-H), 6.91 (dd, ^{3}J = 8.2, ^{4}J = 2.0 Hz, 1H, 6'-H), 7.01 (s, 1H, 5-H), 7.39 (d, ^{3}J = 5.6 Hz, 1H 4-H), 7.42 (s, 1H, 8-H), 8.43 (d, ^{3}J = 5.6 Hz, 1H, 3-H).

The product was fully characterized as isoquinolinium tetrafluoroborate (NMR) or hydrochloride salt (El. Anal.):

1''-(Ethoxy-4''-carbonylpropyl)-1-(3,4-dimethoxybenzyl)-6,7-dimethoxyisoquinolinium tetrafluroborate (**3c-HBF$_4$**). To a stirred solution of **3c** (185 mg, 408 µmol) in Et_2O (200 mL) an aq. solution of HBF_4 (w/w 50%, 100 µL) was added dropwise. The colorless precipitate was filtered off and washed with Et_2O. The product was obtained as a hygroscopic, light yellow amorphous solid (203 mg, 375 µmol, 92%); mp 181–185 °C. – ^{1}H NMR (500 MHz, DMSO-d_6): δ = 1.15 (t, ^{3}J = 7.1 Hz, 3H, 2'''-H), 1.57 (pent., ^{3}J = 7.5 Hz, 2H, 3''-H), 2.29−2.37 (m, 2H, 2''-H), 2.39−2.46 (m, 2H, 4''-H), 3.68 (s, 3H, 4'-OMe), 3.73 (s, 3H, 3'-OMe), 4.02 (s, 3H, 6-OMe or 7-OMe), 4.03 (q, ^{3}J = 6.9 Hz, 1'''-H), 4.04 (s, 3H, 6-OMe or 7-OMe), 5.34 (t, ^{3}J = 7.1 Hz, 1H, 1''-H), 6.89 (d, ^{3}J = 8.4 Hz, 1H, 5'-H), 6.95 (dd, ^{3}J = 8.4 Hz, ^{4}J = 1.9 Hz, 1H, 6'-H), 7.17 (d, ^{4}J = 1.9 Hz, 1H, 2'-H), 7.73 (s, 1H, 5-H or 8-H), 7.97 (s, 1H, 5-H or 8-H), 8.13 (d, ^{3}J = 6.2 Hz, 1H, 4-H), 8.37 (d, ^{3}J = 6.6 Hz, 1H, 3-H). – ^{13}C NMR (125 MHz, DMSO-d_6): δ = 14.6 (C2'''), 23.0 (C3''), 32.5 (C2''), 33.7 (C4''), 45.0 (C1''), 56.0 (4'-OMe), 56.2 (3'-OMe), 57.1 (6-OMe or 7-OMe), 57.2 (6-OMe or 7-OMe), 60.4 (C1'''), 105.8 (C5 or C8), 107.3 (C5 or C8), 112.2 (C2'), 112.7 (C5'), 121.6 (C6'), 121.5 (C8a), 122.2 (C4), 130.6 (C3), 132.1 (C1'), 137.2 (C4a), 148.6 (C4'), 149.5 (C3'), 152.7 (C6 or C7), 156.6 (C6 or C7), 156.7 (C1), 173.2 (C=O).

1''-(Ethoxy-4''-carbonylpropyl)-1-(3,4-dimethoxybenzyl)-6,7-dimethoxyisoquinolinium chloride (**3c-HCl**). To a stirred solution of **3c** (313 mg, 691 µmol) in Et_2O (300 mL), a solution of HCl in Et_2O (1.0 M, 0.8 mL, 0.8 mmol) was added dropwise. The colorless precipitate was filtered off and washed with Et_2O. The product was obtained as a light yellow amorphous solid (330 mg, 674 µmol, >97%); mp 189–190 °C. – El. Anal. for $C_{26}H_{32}ClNO_6$, calcd. (%): C 63.73, H 6.58, N 2.86, found (%): C 62.87, H 6.53, N 2.77.

7-(6,7-Dimethoxyisoquinolin-1-yl)-7-(3,4-dimethoxyphenyl)heptanenitrile (**3d**)

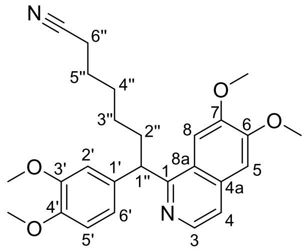

A solution of **3b** (1.00 g, 2.05 mmol) and KCN (200 mg, 3.07 mmol) in MeCN (6 mL) was stirred for 3 d at 85 °C. The solvent was removed under reduced pressure, and the residue was dissolved in Et_2O (150 mL) and water (100 mL). After separation, the organic layer was washed with water (3 × 100 mL) and brine (1 × 100 mL), dried with Na_2SO_4 and filtered. The solvent was removed under reduced pressure and the residue was washed with *n*-pentane (5 × 2 mL) to give **3d** as an orange-colored solid (501 mg, 1.15 mmol, 56%); mp 54–55 °C. – ^{1}H NMR (500 MHz, $CDCl_3$): δ = 1.29–1.44 (m, 2H, 3”-H), 1.45–1.60 (m, 2H, 4”-H), 1.65 (quint, ^3J = 6.9 Hz, 2H, 5”-H), 2.13–2.21 (m, 1H, 2”-H), 2.30 (t, ^3J = 6.9 Hz, 2H, 6”-H), 2.43–2.52 (m, 1H, 2”-H), 3.77 (s, 3H, 3’-OMe), 3.81 (s, 3H, 4’-OMe), 3.94 (s, 3H, 7-OMe), 3.98 (s, 3H, 6-OMe), 4.59 (t, ^3J = 7.3 Hz, 1H, 1”-H), 6.76 (d, ^3J = 8.2 Hz, 1H, 5’-H), 6.85 (d, ^3J = 2.0 Hz, 1H, 2’-H), 6.91 (dd, ^3J = 8.2, ^4J = 2.0 Hz, 1H, 6’-H), 7.02 (s, 1H, 5-H), 7.39 (d, ^3J = 5.7 Hz, 1H, 4-H), 7.42 (s, 1H, 8-H), 8.42 (d, ^3J = 5.7 Hz, 1H, 3-H). – ^{13}C NMR (125 MHz, $CDCl_3$): δ = 17.0 (C6”), 25.2 (C5”), 27.1 (C3”), 28.6 (C4”), 35.5 (C2”), 48.9 (C1”), 55.8 (3’-OMe, 4’-OMe, 6-OMe), 56.0 (C7-OMe), 103.7 (C8), 105.4 (C5), 110.9 (C2’), 111.0 (C5’), 118.4 (C4), 119.9 (6”-CN), 120.1 (C6’), 122.9 (C8a), 133.3 (C4a), 137.0 (C1’), 140.7 (C3), 147.5 (C4’), 149.1 (C3’), 149.7 (C6), 152.2 (C7), 159.7 (C1). – MS (ESI$^+$) for $C_{26}H_{30}N_2O_4$ (434.54 g/mol): *m*/*z* = 435 (100) [M + H]$^+$.

1-(6-Azido-1-(3,4-dimethoxyphenyl)hexyl)-6,7-dimethoxyisoquinoline (**3e**)

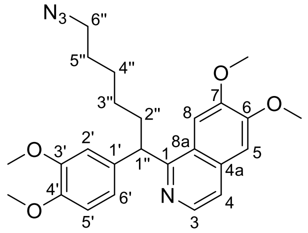

A solution of **3b** (500 mg, 1.02 mmol) and NaN_3 (133 mg, 2.04 mmol) in DMF (10 mL) was stirred for 18 h at 80 °C. After cooling the solution to rt, it was diluted with water (100 mL) and extracted with CH_2Cl_2 (3 × 50 mL). The combined organic layers were washed with water (5 × 100 mL) and brine (3 × 100 mL), dried with Na_2SO_4 and filtered. The solvent was removed under reduced pressure, and the residue was washed with *n*-pentane (1 × 50 mL). The resulting oil was dried under reduced pressure (28 µbar) for 8 h at 80 °C to give **3e** (380 mg, 844 µmol, 83%) as yellow liquid. – ^{1}H NMR (500 MHz, $CDCl_3$): δ = 1.29–1.40 (m, 2H, 3”-H), 1.40–1.51 (m, 2H, 4”-H), 1.59 (quint, ^3J = 7.1 Hz, 2H, 5”-H), 2.11–2.23 (m, 1H, 2”-H), 2.42–2.52 (m, 1H, 2”-H), 3.21 (t, ^3J = 6.9 Hz, 2H, 6”-H), 3.76 (s, 3H, 3’-OMe), 3.81 (s, 3H, 4’-OMe), 3.95 (s, 3H, 7-OMe), 3.98 (s, 3H, 6-OMe), 4.59 (t, ^3J = 7.3 Hz, 1H, 1”-H), 6.76 (d, ^3J = 8.2 Hz, 1H, 5’-H), 6.86 (d, ^3J = 2.0 Hz, 1H, 2’-H), 6.91 (dd, ^3J = 8.2, ^4J = 2.0 Hz, 1H, 6’-H), 7.02 (s, 1H, 5-H), 7.38 (d, ^3J = 5.6 Hz, 1H, 4-H), 7.43 (s, 1H, 8-H), 8.44 (d, ^3J = 5.6 Hz, 1H, 3-H) – ^{13}C-NMR (125 MHz, $CDCl_3$): δ = 26.8 (C4”), 27.6 (C3”), 28.8 (C5”), 35.6 (C2”), 48.9 (C1”), 51.4 (C6”), 55.8 (3’-OMe, 4’-OMe, 6-OMe), 56.0 (C7-OMe), 103.6 (C8), 105.4 (C5), 111.0 (C2’, C5’), 118.3 (C4), 120.1 (C6’), 122.9 (C8a), 133.3 (C4a), 137.1 (C1’), 140.8 (C3), 147.5 (C4’), 149.1 (C3’), 149.7 (C6), 152.2 (C7), 159.9 (C1). – MS (ESI$^+$) for $C_{25}H_{30}N_4O_4$ (450.54 g/mol): *m*/*z* = 451 (100) [M + H]$^+$. – IR (liquid film): $\tilde{\nu}$ = 3050 cm^{-1} (–N_3). – El. Anal. for $C_{25}H_{30}N_4O_4$ × HCl × 0.5 H_2O (496.01), calcd (%): C 60.54, H 6.51, N 11.30, found (%): C 60.70, H 6.51, N 11.26.

1-(6-Azido-1-(3,4-dimethoxyphenyl)hexyl)-6,7-dimethoxyisoquinoline hydrochloride (**3e-HCl**)

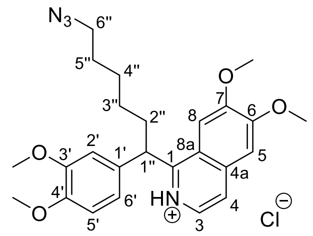

A solution of HCl (1.0 M, 1 mmol) in Et_2O (1 mL) was added to a stirred solution of **3e** (235 mg, 553 µmol) in Et_2O (50 mL), and the suspension was stirred for 20 min at rt. The precipitate was filtered off and washed with *n*-pentane (2 × 10 mL) and Et_2O (1 × 10 mL) to give **3e-HCl** as light yellow solid (167 mg, 343 µmol, 64%); mp 101–102 °C. – ^{1}H NMR (500 MHz, DMSO-*d*$_6$): δ = 1.30 (quint, ^{3}J = 7.4 Hz, 2H, 3''-H), 1.34–1.46 (m, 2H, 4''-H), 1.52 (quint, ^{3}J = 6.8 Hz, 2H, 5''-H), 2.42–2.49 (m, 1H, 2''-H), 2.53–2.60 (m, 1H, 2''-H), 3.28 (t, ^{3}J = 6.8 Hz, 2H, 6''-H), 3.67 (s, 3H, 3'-OMe), 3.75 (s, 3H, 4'-OMe), 4.02 (s, 3H, 7-OMe), 4.05 (s, 3H, 6-OMe), 5.36 (t, ^{3}J = 7.9 Hz, 1H, 1''-H), 6.87 (d, ^{3}J = 8.4 Hz, 1H, 5'-H), 7.06 (dd, ^{3}J = 8.4, ^{4}J = 2.1 Hz, 1H, 6'-H), 7.37 (d, ^{3}J = 2.1 Hz, 1H, 2'-H), 7.73 (s, 1H, 5-H), 8.00 (s, 1H, 8-H), 8.13 (d, ^{3}J = 6.2 Hz, 1H, 4-H), 8.40 (d, ^{3}J = 6.2 Hz, 1H, 3-H), 15.68 (br s, 1H, 2-H).

6-(6,7-Dimethoxyisoquinolin-1-yl)-6-(3,4-dimethoxyphenyl)hexan-1-ol (**3f**)

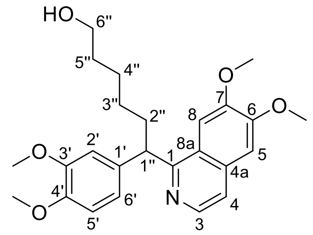

A solution of **3b** (300 mg, 613 µmol) and KOAc (121 mg, 1.23 mmol) in DMF (6 mL) was stirred for 16 h at 120 °C. After cooling the reaction mixture to rt, it was added dropwise to Et_2O (400 mL) and the precipitate was filtered off. The filtrate was washed with water (2 × 100 mL) and brine (1 × 100 mL). The solvent was removed under reduced pressure to yield a colorless solid (178 mg). A solution of the latter and K_2CO_3 (132 mg, 952 µmol) in MeOH (4 mL) was stirred for 12 h at rt. The reaction mixture was diluted with water (25 mL) and extracted with EtOAc (3 × 50 mL). The combined organic layers were washed with water (3 × 100 mL) and brine (3 × 100 mL), dried with Na_2SO_4 and filtered. The solvent was removed under reduced pressure, and the residue was washed with *n*-pentane (2 × 20 mL) to yield the product **3f** as a light brown solid (117 mg, 275 µmol, 45%); mp 98–99 °C. – ^{1}H NMR (500 MHz, $CDCl_3$): δ = 1.28–1.41 (m, 2H, 3''-H), 1.42–1.51 (m, 2H, 4''-H), 1.57 (quint, ^{3}J = 6.9 Hz, 2H, 5''-H), 1.68 (br s, 1H, 6''-OH), 2.12–2.23 (m, 1H, 2''-H), 2.42–2.52 (m, 1H, 2''-H), 3.60 (t, ^{3}J = 6.9 Hz, 2H, 6''-H), 3.76 (s, 3H, 3'-OMe), 3.81 (s, 3H, 4'-OMe), 3.94 (s, 3H, 7-OMe), 3.98 (s, 3H, 6-OMe), 4.60 (t, ^{3}J = 7.3 Hz, 1H, 1''-H), 6.75 (d, ^{3}J = 8.2 Hz, 1H, 5'-H), 6.86 (d, ^{3}J = 2.0 Hz, 1H, 2'-H), 6.91 (dd, ^{3}J = 8.2, ^{4}J = 2.0 Hz, 1H, 6'-H), 7.02 (s, 1H, 5-H), 7.38 (d, ^{3}J = 5.7 Hz, 1H, 4-H), 7.43 (s, 1H, 8-H), 8.44 (d, ^{3}J = 5.7 Hz, 1H, 3-H). –^{13}C NMR (125 MHz, $CDCl_3$): δ = 25.9 (C4''), 27.8 (C3''), 32.7 (C5''), 35.7 (C2''), 49.0 (C1''), 55.8 (3'-OMe, 4'-OMe, 6-OMe), 56.0 (C7-OMe), 63.0 (C6''), 103.8 (C8), 105.4 (C5), 110.8 (C5'), 110.9 (C2'), 118.3 (C4), 120.1 (C6'), 123.0 (C8a), 133.3 (C4a), 137.2 (C1'), 140.7 (C3), 147.5 (C4'), 149.1 (C3'), 149.7 (C6), 152.2 (C7), 160.0 (C1). – MS (ESI^{+}) for $C_{25}H_{31}NO_5$ (425.53 g/mol): *m*/*z* = 426 (100) $[M + H]^{+}$. – El. Anal. for $C_{25}H_{31}NO_5 \times HCl \times 0.5\ H_2O$ (470.99), calcd (%): C 63.75, H 7.06, N 2.97, found (%): C 64.00, H 7.06, N 2.91.

13-(5-Bromopentyl)-2,3,10,11-tetramethoxy-8-methylisoquinolino[3,2-*a*]isoquinolin-7-ium tetrafluoroborate (**2b**)

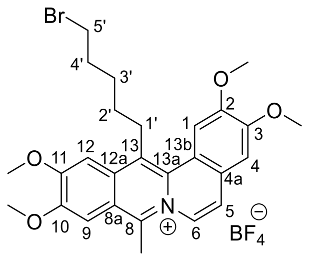

A mixture of H_2SO_4 (735 mg, 400 µL, 7.50 mmol) and acetic anhydride (2.17 g, 2.0 mL, 21.2 mmol) was stirred for 10 min at 90 °C until the mixture turned dark red. Then, **3b** (490 mg, 933 µmol) was added, and the mixture was stirred for 1 h at 90 °C. The reaction mixture was allowed to cool to rt, and MeOH (8 mL) was added dropwise. The solution was stirred for 20 min at rt. The mixture was diluted with water (5 mL), and HBF_4 (w/w 50%, 1 mL) was added. The suspension was extracted with $MeNO_2$ (3 × 50 mL), the organic layer was washed with water (1 × 100 mL), dried with Na_2SO_4 and filtered. The solvent was removed under reduced pressure and the residue was washed with *n*-hexane (1 × 50 mL) and toluene (1 × 50 mL). The crude was crystallized from MeOH and further purified by column chromatography (SiO_2, neutral, CH_2Cl_2/MeOH 96:4, R_f = 0.12) to yield the product **2b** as yellow solid (8.3 mg, 14 µmol, 2%). NMR spectroscopic analysis still showed aliphatic impurities. – ^{1}H NMR (500 MHz, DMSO-d_6): δ = 1.63–1.72 (m, 2H, 3'-H), 1.83 (quint, ^3J = 6.7 Hz, 1H, 4'-H), 1.91 (quint, ^3J = 6.7 Hz, 1H, 4'-H), 2.01–2.10 (m, 2H, 2'-H), 3.39 (s, 3H, 8-Me), 3.56 (t, ^3J = 6.7 Hz, 1H, 5'-H), 3.66 (t, ^3J = 6.7 Hz, 1H, 5'-H), 3.79 (t, ^3J = 8.2 Hz, 2H, 1'-H), 4.02 (s, 3H, 2-OMe), 4.04 (s, 3H, 3-OMe), 4.12 (s, 3H, 10-OMe), 4.20 (s, 3H, 11-OMe), 7.62 (s, 1H, 12-H), 7.72 (s, 1H, 4-H), 7.84 (s, 1H, 1-H), 7.89 (d, ^3J = 7.7 Hz, 1H, 5-H), 7.91 (s, 1H, 9-H), 8.70 (d, ^3J = 7.7 Hz, 1H, 6-H). – ^{13}C NMR (125 MHz, $CDCl_3$): δ = 18.0 (8-Me), 27.7 (C3''), 28.9 (C2''), 31.2 (C1''), 31.9 (C4''), 43.5 (C5''), 56.1 (2-OMe, 3-OMe), 56.6 (2-OMe, 3-OMe), 103.0 (C12), 105.4 (C9), 108.1 (C4), 110.7 (C1), 119.2 (C13b), 119.9 (C5), 121.4 (C8a), 125.2 (C4a), 125.3 (C6), 130.0 (C13), 132.6 (C12a), 135.0 (C13a), 145.4 (C8), 145.9 (C3), 151.9 (C2), 152.2 (C10), 155.8 (C11). – MS (ESI$^+$) for $C_{27}H_{31}BBrF_4NO_4$ (600.26 g/mol): *m*/*z* = 512 (100), 514 (98.8) [M – BF_4]$^+$.

Attempted coralyne synthesis with **3d**

A mixture of H_2SO_4 (0.16 g, 74 µL, 1.4 mmol) and acetic anhydride (400 mg, 370 µL, 3.90 mmol) was stirred for 10 min at 90 °C until the mixture turned dark red. Papaverine **3d** (100 mg, 205 µmol) was added and stirring was continued for 1 h at 90 °C. The reaction mixture was allowed to cool to rt, MeOH (1.3 mL) was added dropwise, and the solution was stirred for 20 min at rt. The reaction mixture was extracted with $MeNO_2$ (3 × 25 mL), the organic layer was washed with water (1 × 50 mL), dried with Na_2SO_4 and filtered. The solvent was removed under reduced pressure to yield a brown oil (97.3 mg). NMR-spectroscopic analysis of the residue indicated decomposition.

13-(Ethoxy-3'-carbonylpropyl)-2,3,10,11-tetramethoxy-8-methylisoquinolino[3,2-*a*]isoquinolin-7-ium, sulfoacetate salt

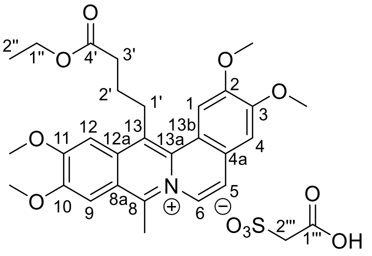

A mixture of H_2SO_4 (3.31 g, 1.80 mL, 32.4 mmol) and acetic anhydride (7.56 g, 7.00 mL, 74.1 mmol) was stirred for 15 min at 90 °C until the mixture turned dark red. A solution of **3c** (2.64 g, 5.81 mmol) in acetic anhydride (7.00 mL) was added, and stirring was continued for 1 h at 90 °C. The reaction mixture was cooled to 0 °C, iPrOH (40 mL) was added, and stirring was continued for 20 min at this temperature. The resulting precipitate was filtered off and washed with Et_2O (3 × 25 mL) and *n*-pentane (3 × 25 mL) to yield the product as a yellow powder (1.34 g, 2.17 mmol, 37%); mp 274–275 °C. – ^{1}H NMR (500 MHz, DMSO-d_6): δ = 1.17 (t, ^3J = 7.1 Hz, 3H, 2''-H), 2.17–2.31 (m, 2H, 2'-H), 2.63 (t, ^3J = 6.5 Hz, 2H, 3'-H), 3.36 (s, 3H, 8-Me), 3.39 (s, 1H, 2'''-H), 3.82 (t, ^3J = 8.3 Hz, 2H, 1'-H), 4.01 (s, 3H, 2-OMe), 4.02 (s, 3H, 3-OMe), 4.06 (q, ^3J = 7.1 Hz, 2H, 1''-H), 4.12 (s, 3H, 10-OMe), 4.22 (s, 3H, 11-OMe), 7.71 (s, 1H, 4-H), 7.83 (s, 1H, 1-H), 7.88 (d, ^3J = 7.7 Hz, 1H, 5-H), 7.90 (s, 2H, 9-H, 12-H), 8.69 (d, ^3J = 7.7 Hz, 1H, 6-H). – ^{13}C NMR (125 MHz, DMSO-d_6): δ = 14.1 (C2''), 18.0 (8-Me), 24.7 (C2'), 30.7 (C1'), 32.8 (C3'), 55.8 (3-OMe), 56.1 (2-OMe), 56.6 (10-OMe), 56.9 (11-OMe), 57.1 (C2'''), 60.1 (C1''), 103.1 (C12), 105.4 (C9), 108.0 (C4), 110.6 (C1), 119.0 (C13b), 119.5 (C5), 121.4 (C8a), 125.2 (C4a, C6), 129.4 (C13), 132.9 (C12a), 135.1 (C13a), 145.5 (C8), 149.3 (C3), 151.8 (C2), 152.2 (C10), 156.7 (C11), 167.3 (C1'''), 173.0 (C4').

13-(Ethoxy-3'-carbonylpropyl)-2,3,10,11-tetramethoxy-8-methyliso-quinolino[3,2-*a*]isoquinolin-7-ium (**2c**), tetrafluoroborate salt

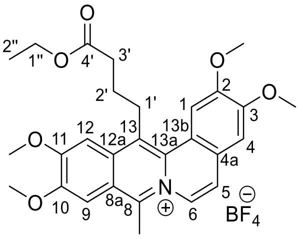

An aq. solution of HBF_4 (w/w 50%,1.50 mL, 12.2 mmol) was added dropwise to a stirred solution of 13-(ethoxy-3'-carbonylpropyl)-2,3,10,11-tetramethoxy-8-methylisoquinolino[3,2-*a*]isoquinolin-7-ium sulfoacetate (922 mg, 1.49 mmol) in water (515 mL), and stirring was continued for 10 min at rt. The resulting precipitate was filtered off and was washed with Et_2O (3 × 20 mL) and *n*-pentane (2 × 20 mL) to give the tetrafluoroborate salt of **2c** as a yellow powder (727 mg, 1.29 mmol, 87%); mp 266–267 °C (dec.). – ^{1}H NMR (500 MHz, DMSO-d_6): δ = 1.17 (t, ^3J = 7.1 Hz, 3H, 2''-H), 2.18–2.31 (m, 2H, 2'-H), 2.63 (t, ^3J = 6.5 Hz, 2H, 3'-H), 3.38 (s, 3H, 8-Me), 3.83 (t, ^3J = 8.3 Hz, 2H, 1'-H), 4.02 (s, 6H, 2-OMe, 3-OMe), 4.06 (q, ^3J = 7.1 Hz, 2H, 1''-H), 4.12 (s, 3H, 10-OMe), 4.22 (s, 3H, 11-OMe), 7.71 (s, 1H, 4-H), 7.83 (s, 1H, 1-H), 7.89 (d, ^3J = 7.7 Hz, 1H, 5-H), 7.90 (s, 2H, 9-H, 12-H), 8.69 (d, ^3J = 7.7 Hz, 1H, 6-H). – ^{13}C NMR (125 MHz, DMSO-d_6): δ = 14.0 (C2''), 18.0 (8-Me), 24.7 (C3'), 30.7 (C1'), 32.8 (C2'), 55.8 (3-OMe), 56.1 (2-OMe), 56.6 (10-OMe), 56.9 (11-OMe), 60.1 (C1''), 103.1 (C12), 105.4 (C9), 108.0 (C1), 110.7 (C4), 119.1 (C4a, C13b), 119.9 (C5), 121.4 (C8a), 125.2 (C6), 129.5 (C12a), 132.9 (C13), 135.1 (C13a), 145.5 (C8), 149.3 (C3), 151.8 (C2), 152.2 (C10), 156.7 (C11), 173.0 (C4'). – El. Anal. for $C_{28}H_{32}BF_4NO_6$, calcd. (%): C 59.48, H 5.71, N 2.48, found (%): C 59.44, H 5.37, N 2.41.

13-(3'-Carboxypropyl)-2,3,10,11-tetramethoxy-8-methyliso-quinolino[3,2-*a*]isoquinolin-7-ium chloride (**2d**)

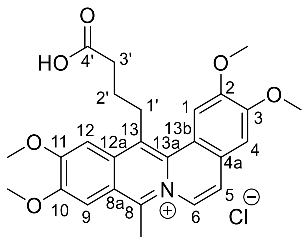

A solution of **2c** (tetrafluorborate salt) (722 mg, 1.28 mmol) in aq. HCl (3 M, 870 mL) was stirred at 110 °C for 30 min. The solution was cooled to 0 °C, and the resulting precipitate was filtered off. The solid was washed with H_2O (1 × 10 mL), ice cold MeCN (1 × 1 mL), ice cold MeOH (1 × 1 mL), Et_2O (3 × 10 mL) and *n*-pentane (3 × 20 mL) to give **2d** as a yellow powder (580 mg, 1.19 mmol, 93%); mp 204–205 °C (dec.). – ^{1}H NMR (500 MHz, DMSO-d_6): δ = 2.19–2.35 (m, 2H, 2'-H), 2.63 (t, ^3J = 6.4 Hz, 2H, 3'-H), 3.39 (s, 3H, 8-Me), 3.77 (t, ^3J = 8.5 Hz, 2H, 1'-H). 4.01 (s, 3H, 2-OMe), 4.03 (s, 3H, 3-OMe), 4.12 (s, 3H, 10-OMe), 4.21 (s, 3H, 11-OMe), 7.71 (s, 1H, 4-H), 7.83 (s, 1H, 1-H), 7.88 (d, ^3J = 7.7 Hz, 1H, 5-H), 7.90 (s, 1H, 9-H), 8.02 (s, 1H, 12-H), 8.70 (d, ^3J = 7.7 Hz, 1H, 6-H), 11.39–13.56 (br s, 1H, CO_2H). – ^{13}C NMR (125 MHz, DMSO-d_6): δ = 18.6 (8-Me), 25.2 (C2'), 31.5 (C1'), 33.6 (C3'), 56.4 (3-OMe), 56.7 (2-OMe), 57.1 (10-OMe), 57.6 (11-OMe), 103.8 (C12), 106.0 (C9), 108.6 (C4), 111.2 (C1), 119.7 (C13b), 120.5 (C5), 122.0 (C8a), 125.7 (C6), 125.8 (C4a), 130.2 (C13), 133.5 (C12a), 135.6 (C13a), 146.1 (C8), 149.8 (C3), 152.3 (C2), 156.7 (C10), 157.3 (C11), 175.2 (C4'). – MS (ESI$^+$) for $C_{26}H_{28}NO_6Cl$ (485.96 g/mol): *m*/*z* = 450 (100) [M – Cl]$^+$.– El. Anal. for $C_{26}H_{28}NO_6Cl \times 2\ H_2O$ (485.96), calcd (%): C 59.83, H 6.18, N 2.68, found (%): C 59.83, H 6.00, N 2.67.

13-(4'-((4''-Ethoxy-3'-carbonylpropyl)amino)-4'-oxobutyl)-2,3,10,11-tetramethoxy-8-methylisoquinolino[3,2-*a*]isoquinolin-7-ium bromide (**2e**)

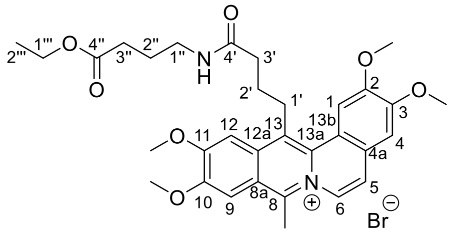

Anhydrous DIPEA (239 mg, 317 µL, 1.85 mmol) was added to a solution of **2d** (300 mg, 617 mmol), ethyl 4-aminobutanoate hydrochloride (124 mg, 740 µmol) and PyBOP (353 mg, 679 µmol) in anhydrous DMF (60 mL), and the mixture was stirred for 16 h at rt under Ar atmosphere. The reaction mixture was added dropwise to Et_2O (1.5 L), and the resulting precipitate was filtered off. The crude product was washed with Et_2O (3 × 20 mL) and *n*-pentane (3 × 20 mL) to give the product **2e** as an dark orange-colored solid (384 mg; mp. 181–182 °C; mixed chloride/hexafluorophosphate salt). A solution of the latter (381 mg) in MeOH (250 mL) was passed through an ion exchange resin (Amberlite IRA-900, Br$^-$). The solvent was removed under reduced pressure, and the crude was purified by column chromatography (SiO_2, neutral, CH_2Cl_2/MeOH, 94/6 R_f = 0.13) to give the product **2e** (bromide salt) as a yellow powder (276 mg, 429 µmol, 70% over two steps). An analytically pure sample was obtained by crystallization form *n*-hexane/*i*-PrOH; mp 185–186 °C. – ^{1}H NMR (600 MHz, DMSO-d_6): δ = 1.13 (t, ^3J = 7.1 Hz, 3H, 2'''-H), 1.66 (quint, ^3J = 7.0 Hz, 2H, 2''-H), 2.22–2.41 (m, 4H, 2'-H, 3''-H), 2.47 (t, ^3J = 6.3 Hz, 2H, 3'-H), 3.11 (q, ^3J = 7.0 Hz, 2H, 1''-H), 3.38 (s,3H, 8-Me), 3.65 (t, ^3J = 7.5 Hz, 2H, 1'-H), 3.99–4.03 (m, 5H, 2-OMe, 1'''-H), 4.04 (s, 3H, 3-OMe), 4.11 (s, 3H, 10-OMe), 4.23 (s, 3H, 11-OMe), 7.71 (s, 1H, 4-H), 7.80 (s, 1H, 1-H), 7.87 (d, ^3J = 7.7 Hz, 1H, 5-H), 7.88 (s, 1H, 9-H), 7.97 (s, 1H, 12-H), 8.03 (t, ^3J = 7.0 Hz, 1H, 4'-NH), 8.69 (d, ^3J = 7.7 Hz, 1H, 6-H). – ^{13}C NMR (150 MHz, DMSO-d_6): δ = 14.1 (C2'''), 18.1 (8-Me), 24.5 (C2''), 25.0 (C2'), 30.9 (C3''), 31.1 (C1'), 34.3 (C3'), 37.8 (C1''), 55.8 (3-OMe), 56.1 (2-OMe), 56.6 (10-OMe), 57.1 (11-OMe), 59.7 (C1'''), 103.2 (C12), 105.3 (C9), 108.1 (C4), 110.4 (C1), 119.1 (C13b),

120.0 (C5), 121.4 (8a), 125.2 (C6), 125.3 (C4a), 129.6 (C13), 133.0 (C12a), 134.9 (C13a), 145.5 (C8), 149.2 (C3), 151.7 (C2), 152.2 (C10), 156.8 (C11), 171.7 (C4’), 172.6 (C4”). – El. Anal. for $C_{32}H_{39}N_2O_7Br \times 1.5\ H_2O$ (670.60), calcd (%): C 57.31, H 6.31, N 4.18, found (%): C 57.32, H 5.95, N 4.01.

13-(2,2-Dimethyl-4,11-dioxo-3,6-dioxa-5,10-diazatetradecan-14-yl)-2,3,10,11-tetramethoxy-8-methylisoquinolino[3,2-*a*]isoquinolin-7-ium, bromide salt

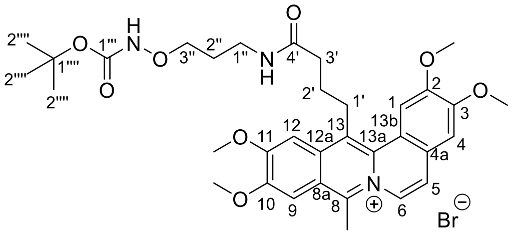

Anhydrous DIPEA (172 mg, 240 µL, 1.33 mmol) was added to a solution of **2d** (200 mg, 412 µmol), 1,1-dimethylethyl *N*-(3-aminopropoxy)carbamate hydrochloride (111 mg, 488 µmol) and PyBOP (232 mg, 444 µmol) in anhydrous DMF (40 mL), and the mixture was stirred for 16 h at rt under an Ar atmosphere. The reaction mixture was added dropwise to Et_2O (1.4 L), and the resulting precipitate was filtered off. The solid was washed with Et_2O (3 × 20 mL), cold MeOH (1 × 1 mL), and *n*-pentane (3 × 20 mL) to give the product as a dark yellow solid (268 mg; mp 178–179 °C; mixed chloride/hexafluorophosphate salt). A solution of the latter (620 mg) in MeOH (400 mL) was passed through an ion exchange resin (Amberlite IRA-900, Br^-). The solvent was removed under reduced pressure, and the crude was purified by column chromatography (SiO_2, neutral, $CHCl_3$/MeOH 92/8, R_f = 0.14) to give the bromide salt as a yellow powder (555 mg, 789 µmol, 77% over two steps); mp 180–181 °C (dec.). – ^{1}H NMR (600 MHz, DMSO-d_6): δ = 1.32 (s, 9H, 2’’’’-H), 1.66 (quint, ^3J = 6.5 Hz, 2H, 2”-H), 2.27–2.41 (m, 2H, 2’-H), 2.47 (t, ^3J = 6.2 Hz, 2H, 3’-H), 3.17 (q, ^3J = 6.5 Hz, 2H, 1”-H), 3.37 (s, 3H, 8-Me), 3.62 (t, ^3J = 8.4 Hz, 2H, 1’-H), 3.71 (t, ^3J = 6.5 Hz, 2H, 3”-H), 4.00 (s, 3H, 2-OMe), 4.03 (s, 3H, 3-OMe), 4.11 (s, 3H, 10-OMe), 4.23 (s, 3H, 11-OMe), 7.70 (s, 1H, 4-H), 7.77 (s, 1H, 1-H), 7.86 (s, 1H, 9-H), 7.87 (d, ^3J = 7.7 Hz, 1H, 5-H), 7.95 (s, 1H, 12-H), 8.04 (t, ^3J = 6.5 Hz, 1H 4’-NH), 8.69 (d, ^3J = 7.7 Hz, 1H, 6-H), 9.94 (s, 1H, 3”-ONH). – ^{13}C NMR (150 MHz, DMSO-d_6): δ = 18.2 (8-Me), 25.0 (C2’), 27.7 (C2”), 27.9 (C2’’’’), 31.1 (C1’), 34.3 (C3’), 35.7 (C1”), 55.8 (3-OMe), 56.1 (2-OMe), 56.6 (10-OMe), 57.1 (11-OMe), 73.3 (C3”), 79.5 (C1’’’), 103.2 (C12), 105.3 (C9), 108.1 (C4), 110.3 (C1), 119.0 (C13b), 119.9 (C5), 121.3 (C8a), 125.2 (C6), 125.3 (C4a), 129.5 (C13), 132.9 (C12a), 134.8 (C13a), 145.4 (C8), 149.2 (C3), 151.7 (C2), 152.2 (C10), 156.8 (C11), 156.8 (C1’’’), 171.6 (C4’). – HRMS (ESI$^+$) for $C_{34}H_{44}N_3O_8$ (calcd for cation: 622.3123): *m*/*z* = 622.3106 (44) [M – Br]$^+$. – El. Anal. for $C_{34}H_{44}N_3O_8Br \times 1.5\ H_2O$ (729.67), calcd (%): C 55.97, H 6.49, N 5.76, found (%): C 56.00, H 6.31, N 5.62.

13-(2,2-Dimethyl-4,11-dioxo-3,6-dioxa-5,10-diazatetradecan-14-yl)-2,3,10,11-tetramethoxy-8-methylisoquinolino[3,2-a]isoquinolin-7-ium tetrafluoroborate (**2f**)

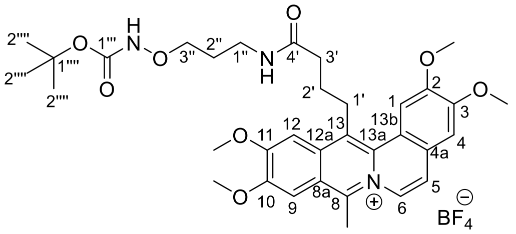

A solution of $NaBF_4$ (80.0 mg, 725 µmol) in H_2O (1 mL) was added dropwise to a stirred solution of the bromide salt of **2f** (50.0 mg, 72.5 µmol) in H_2O (35 mL), and stirring was continued for 20 min at rt. The resulting precipitate was filtered off, and was washed with Et_2O (3 × 5mL) and *n*-pentane (1 × 5 mL) to give the tetrafluorborate salt **2f** as a yellow powder (48.7 mg, 68.6 µmol, 95%); mp 177–178 °C (dec.). – ^{1}H NMR (600 MHz, DMSO-d_6): δ = 1.32 (s, 9H, 2’’’’-

H), 1.66 (quint, ^3J = 6.5 Hz, 2H, 2''-H), 2.16–2.41 (m, 2H, 2'-H), 2.46 (t, ^3J = 6.2 Hz, 2H, 3'-H), 3.17 (q, ^3J = 6.5 Hz, 2H, 1''-H), 3.37 (s, 3H, 8-Me), 3.65 (t, ^3J = 8.4 Hz, 2H, 1'-H), 3.71 (t, ^3J = 6.5 Hz, 2H, 3''-H), 4.01 (s, 3H, 2-OMe), 4.03 (s, 3H, 3-OMe), 4.11 (s, 3H, 10-OMe), 4.24 (s, 3H, 11-OMe), 7.70 (s, 1H, 4-H), 7.81 (s, 1H, 1-H), 7.89 (d, ^3J = 7.7 Hz, 1H, 5-H), 7.88 (s, 1H, 9-H), 7.98 (s, 1H, 12-H), 8.00 (t, ^3J = 6.5 Hz, 1H 4'-NH), 8.69 (d, ^3J = 7.7 Hz, 1H, 6-H), 9.94 (s, 1H, 3''-ONH). – ^{13}C-NMR (150 MHz, DMSO-*d*$_6$): *δ* = 18.0 (8-Me), 25.0 (C2'), 27.7 (C2''), 27.9 (C2'''), 31.0 (C1'), 34.3 (C3'), 35.7 (C1''), 55.8 (3-OMe), 56.1 (2-OMe), 56.6 (10-OMe), 57.1 (11-OMe), 73.3 (C3''), 79.5 (C1'''), 103.3 (C12), 105.3 (C9), 108.1 (C4), 110.4 (C1), 119.1 (C13b), 120.2 (C5), 121.4 (C8a), 125.2 (C6), 125.3 (C4a), 129.6 (C13), 133.0 (C12a), 134.9 (C13a), 145.5 (C8), 149.2 (C3), 151.8 (C2), 152.2 (C10), 156.2 (C11), 156.8 (C1'''), 171.6 (C4'). – El. Anal. for $C_{34}H_{44}N_3O_8BF_4 \times 0.5\ H_2O$ (718.55), calcd (%): C 56.83, H 6.31, N 5.58, found (%): C 56.60, H 6.00, N 5.55.

13-(4-((3-(Aminooxy)propyl)amino)-4-oxobutyl)-2,3,10,11-tetramethoxy-8-methylisoquinolino[3,2-*a*]isoquinolin-7-ium (**2g**)

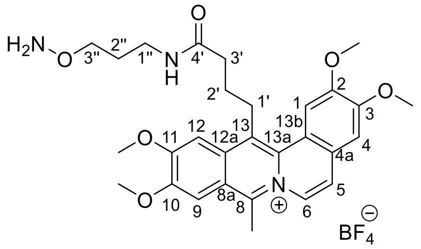

A solution of **2f** (50.0 mg, 70.4 µmol) in CH_2Cl_2/TFA 1:1 (5.0 mL) was stirred for 3 h at rt under Ar atmosphere. The solvent was evaporated under a N_2 stream, and the residue was washed with Et_2O (3 × 5 mL) to give the crude product **2g** as an orange-colored powder (57.9 mg, <70.4 µmol, > 99%); mp 141–142 °C (dec.). – ^{1}H NMR (500 MHz, DMSO-*d*$_6$): *δ* = 1.73 (quint, ^3J = 6.5 Hz, 2H, 2''-H), 2.23–2.41 (m, 2H, 2'-H), 2.47 (t, ^3J = 6.1 Hz, 2H, 3'-H), 3.14 (q, ^3J = 6.5 Hz, 2H, 1''-H), 3.39 (s, 3H, 8-Me), 3.70 (t, ^3J = 8.3 Hz, 2H, 1'-H), 3.97 (t, ^3J = 6.5 Hz, 2H, 3''-H), 4.02 (s, 3H, 2-OMe), 4.05 (s, 3H, 3-OMe), 4.12 (s, 3H, 10-OMe), 4.25 (s, 3H, 11-OMe), 7.73 (s, 1H, 4-H), 7.85 (s, 1H, 1-H), 7.89 (d, ^3J = 7.7 Hz, 1H, 5-H), 7.90 (s, 1H, 9-H), 8.01 (s, 1H, 12-H), 8.08 (t, ^3J = 6.5 Hz, 1H 4'-NH), 8.71 (d, ^3J = 7.7 Hz, 1H 6-H), 10.67 (br s, 2H, 3''-ONH$_2$). – ^{13}C NMR (125 MHz, DMSO-*d*$_6$): *δ* = 18.1 (8-Me), 25.0 (C2'), 27.5 (C2''), 31.1 (C1'), 34.3 (C3'), 35.2 (C1''), 55.8 (3-OMe), 56.1 (2-OMe), 56.6 (10-OMe), 57.1 (11-OMe), 72.1 (C3''), 103.3 (C12), 105.4 (C9), 108.1 (C4), 110.5 (C1), 119.1 (C13b), 119.9 (C5), 121.4 (C8a), 125.2 (C6), 125.3 (C4a), 129.7 (C13), 133.0 (C12a), 135.0 (C13a), 145.5 (C8), 149.3 (C3), 151.8 (C2), 152.2 (C10), 156.2 (C11), 156.8 (C1'''), 171.8 (C4').

13-(4-((3-((Benzylideneamino)oxy)propyl)amino)-4-oxobutyl)-2,3,10,11-tetramethoxy-8-methyl-isoquinolino[3,2-*a*]isoquinolin-7-ium tetrafluoroborate (**2h**)

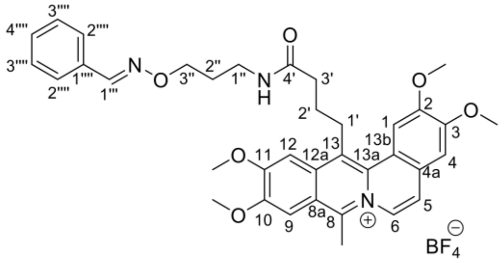

A solution of **2f** (20.0 mg, 28.2 µmol) and benzaldehyde (14.9 mg, 14.2 µL, 141 µmol) in CH_2Cl_2/TFA 1:1 (4.0 mL) was stirred for 4 h at rt under Ar atmosphere. The solvent was evaporated under a N_2 stream, and the residue was washed with Et_2O (3 × 5 mL) to give the product **2f** as an orange-colored powder (16.9 mg, 24.2 µmol, 86%); mp 89–90 °C (dec.). – ^{1}H NMR (600 MHz, DMSO-*d*$_6$): *δ* = 1.81 (quint, ^3J = 6.9 Hz, 2H, 2''-H), 2.25–2.40 (m, 2H, 2'-H),

2.48 (t, ^3J = 6.5 Hz, 2H, 3'-H), 3.21–3.26 (q, ^3J = 6.3 Hz, 2H, 1''-H), 3.35 (s, 3H, 8-Me), 3.63 (t, ^3J = 8.1 Hz, 2H, 1'-H), 4.02 (s, 3H, 2-OMe), 4.04 (s, 3H, 2-OMe), 4.09–4.13 (m, 5H, 10-OMe, 3''-H), 4.23 (s, 3H, 11-OMe), 7.26 (t, ^3J = 7.4 Hz, 2H, 3''''-H), 7.33 (t, ^3J = 7.4 Hz, 1H, 4''''-H), 7.37–7.42 (m, 2H, 2''''-H), 7.71 (s, 1H, 4-H), 7.81 (s, 1H, 1-H), 7.85–7.89 (m, 2H, 5-H, 9-H), 7.94 (s, 1H, 12-H), 8.06 (t, ^3J = 5.7 Hz, 1H, 4'-NH), 8.12 (s, 1H, 1'''-H), 8.68 (d, ^3J = 7.7 Hz, 1H, 6-H). – ^{13}C NMR (150 MHz, DMSO-d_6): δ =18.1 (8-Me), 25.1 (C2'), 28.6 (C2''), 31.0 (C1'), 34.4 (C3'), 35.5 (C1''), 55.8 (3-OMe), 56.1 (2-OMe), 56.6 (10-OMe), 57.2 (11-OMe), 71.2 (C3''), 103.2 (C12), 105.3 (C9), 108.1 (C4), 110.3 (C1), 119.1 (C13b), 119.9 (C5), 121.4 (C8a), 125.2 (C6), 125.3 (C4a), 126.5 (C2''''), 128.5 (C3''''), 129.7 (C13, C4''''), 131.8 (C1''''), 132.9 (C12a), 134.9 (C13a), 145.4 (C8), 148.5 (C1'''), 149.3 (C3), 151.7 (C2), 152.2 (C10), 156.8 (C11), 171.7 (C4'). – MS (ESI$^+$) for $C_{36}H_{40}BF_4N_3O_6$ (697.54 g/mol): m/z = 610 (100) [M – BF_4]$^+$.

2. NMR spectra

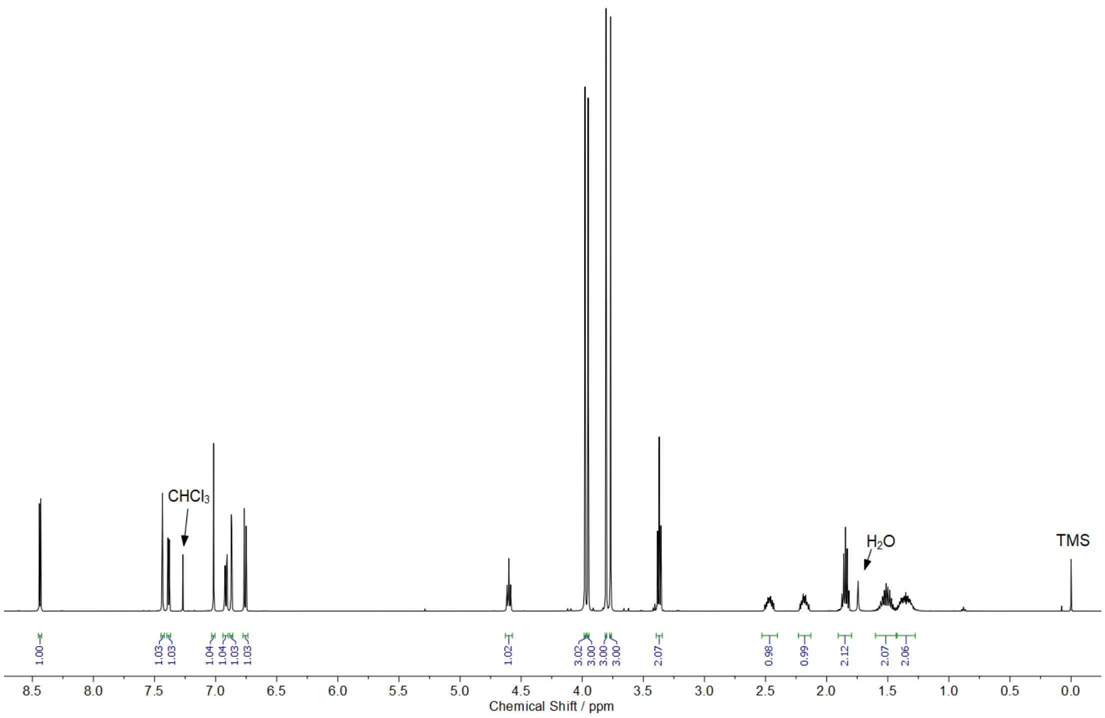

Figure S1. ^{1}H NMR spectrum (500 MHz, $CDCl_3$) of **3b**.

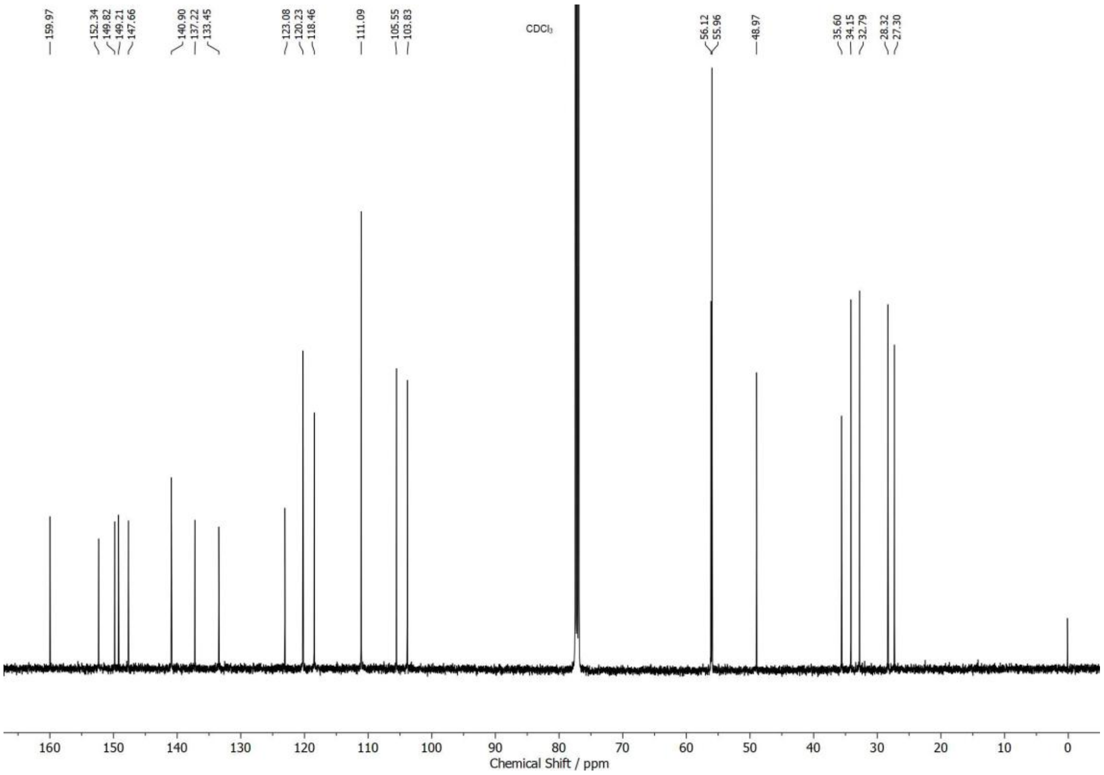

Figure S2. ^{13}C NMR spectrum (125 MHz, $CDCl_3$) of **3b**.

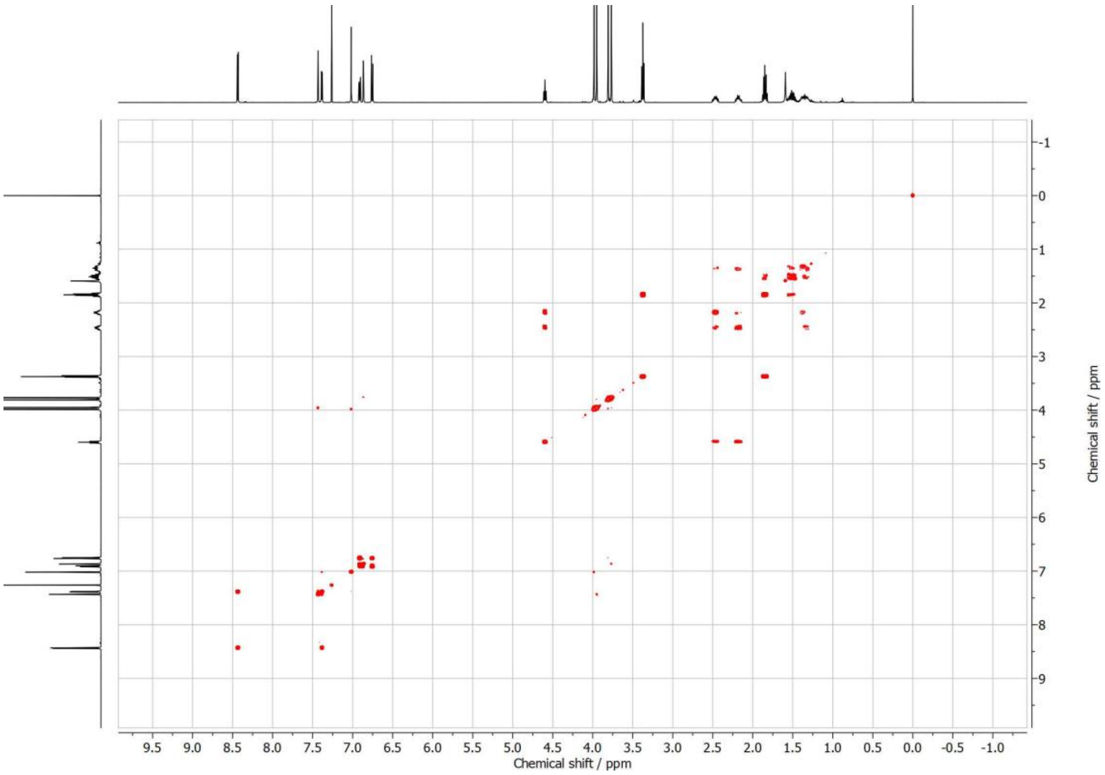

Figure S3. $^{1}H^{1}H$ COSY NMR spectrum (500 MHz, $CDCl_3$) of **3b**.

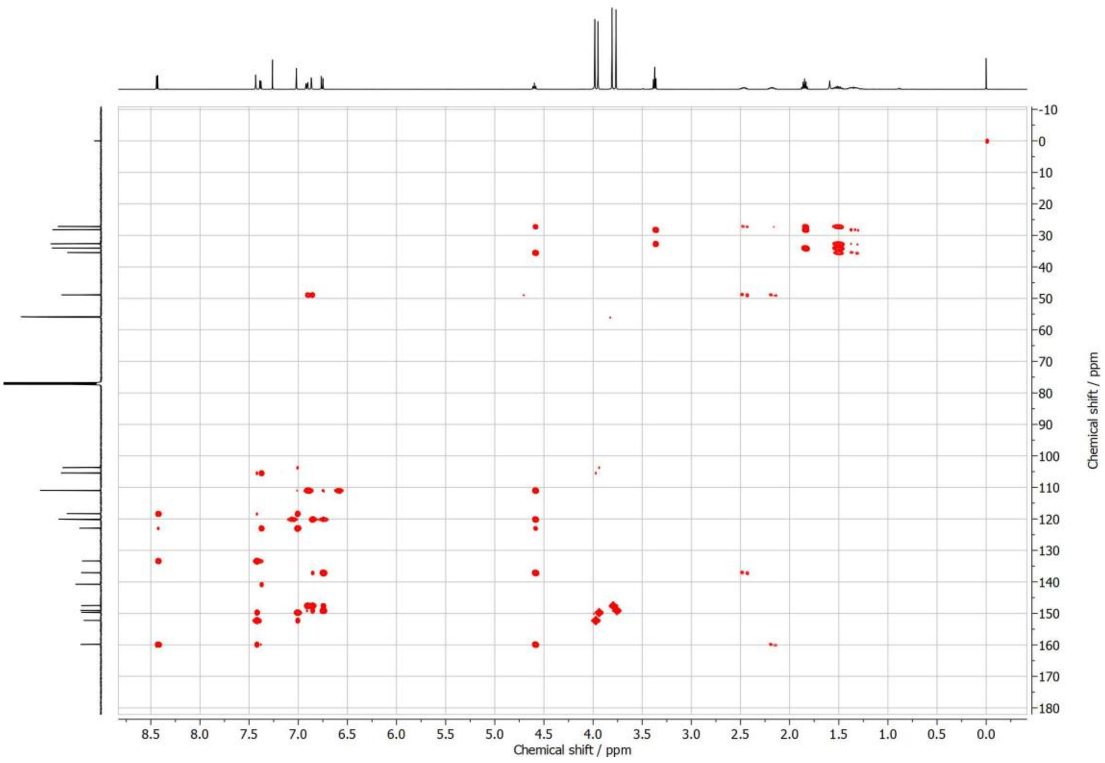

Figure S4. $^{1}H^{13}C$ HMBC NMR spectrum (500 MHz, $CDCl_3$) of **3b**.

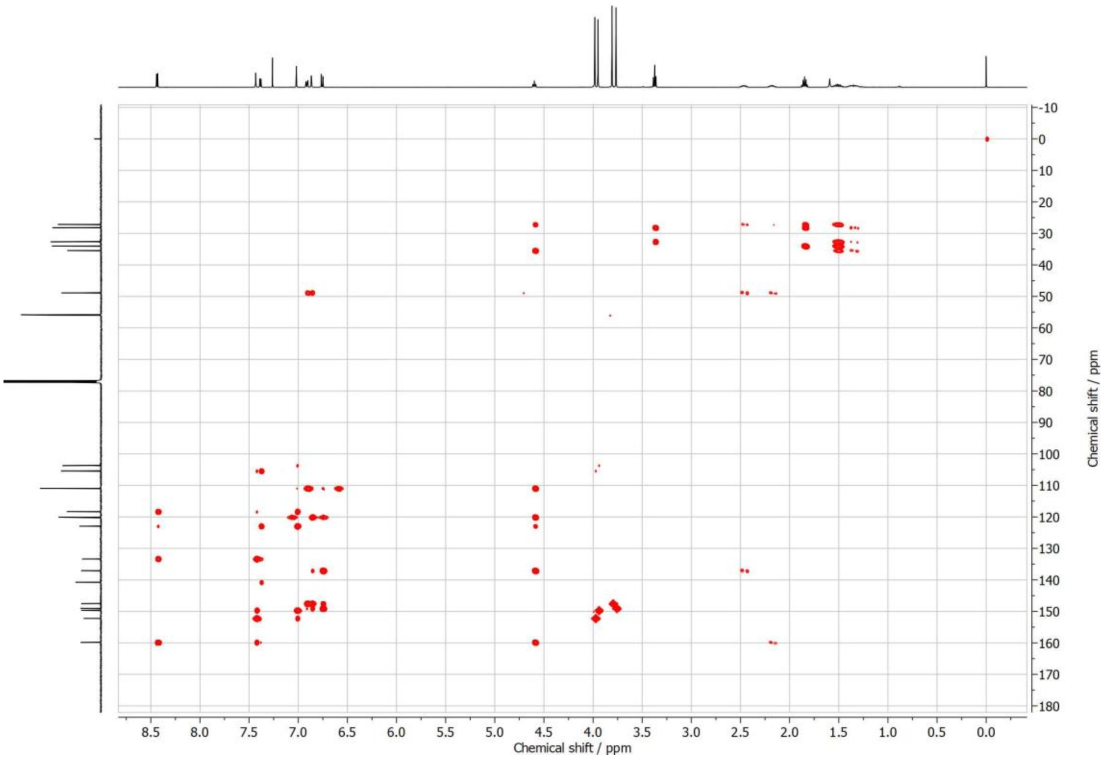

Figure S5. ^{1}H^{13}C HMBC NMR spectrum (500 MHz, $CDCl_3$) of **3b**.

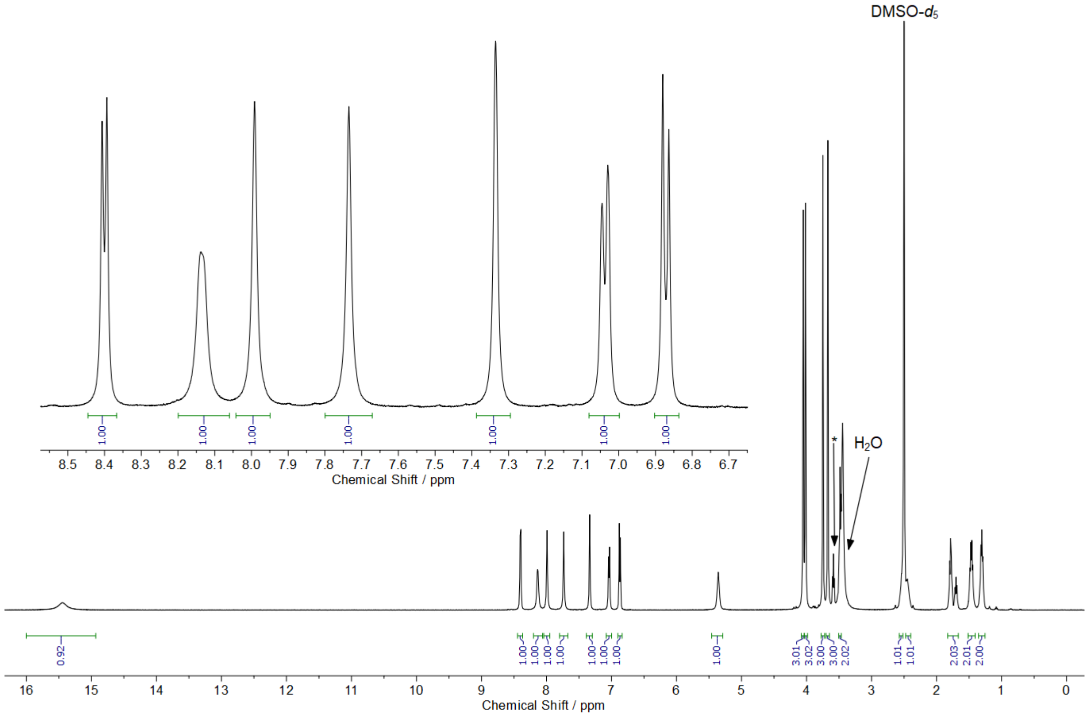

Figure S6. ^{1}H NMR spectrum (500 MHz, DMSO-d_6) of **3b-HCl**

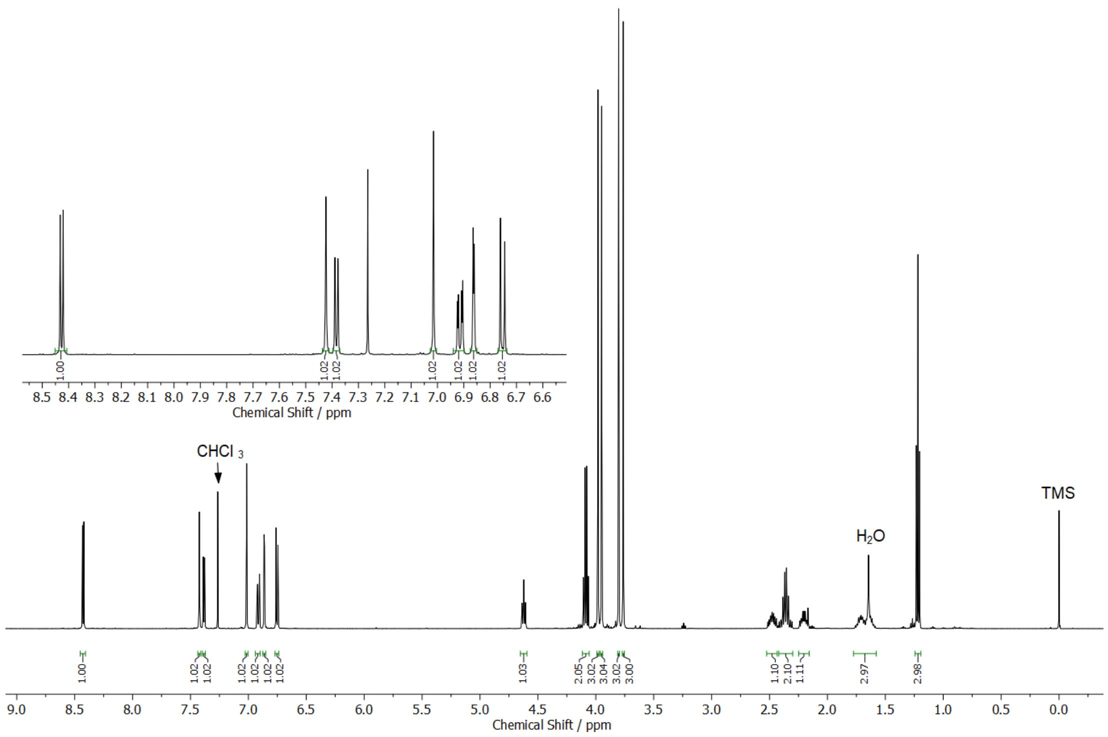

Figure S7. ^{1}H NMR spectrum (500 MHz, $CDCl_3$) of **3c**.

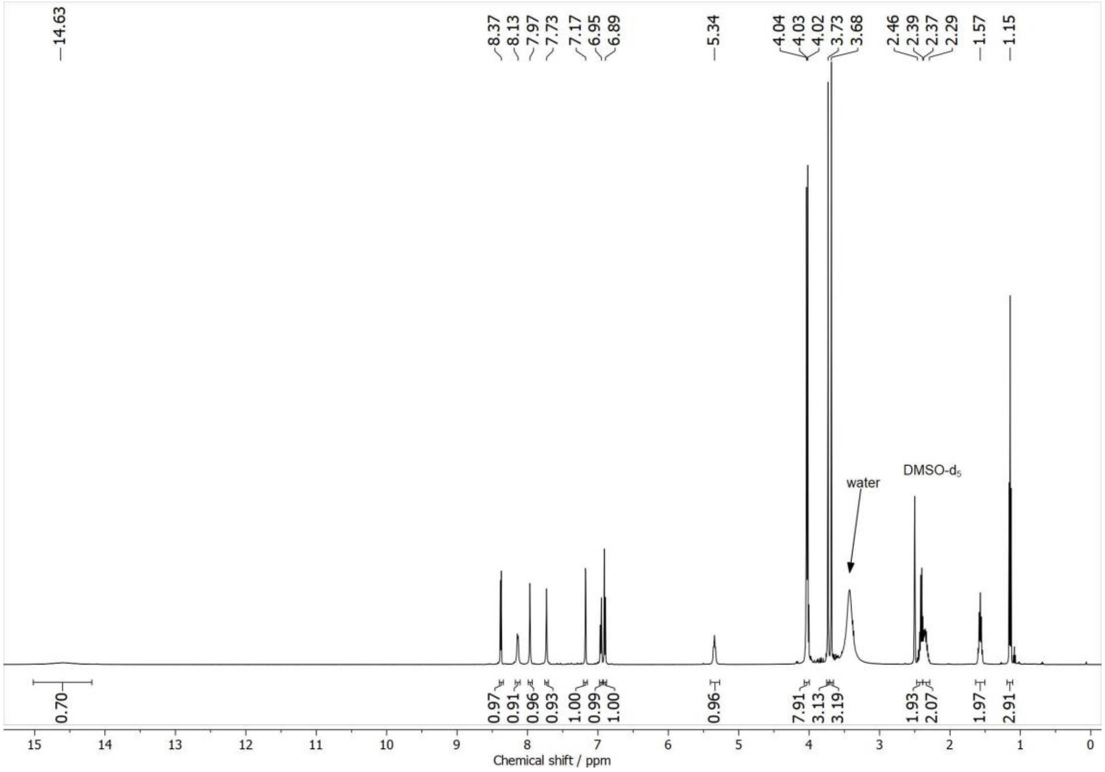

Figure S8. ^{1}H NMR spectrum (500 MHz, DMSO-*d*$_6$) of **3c-HBF$_4$**.

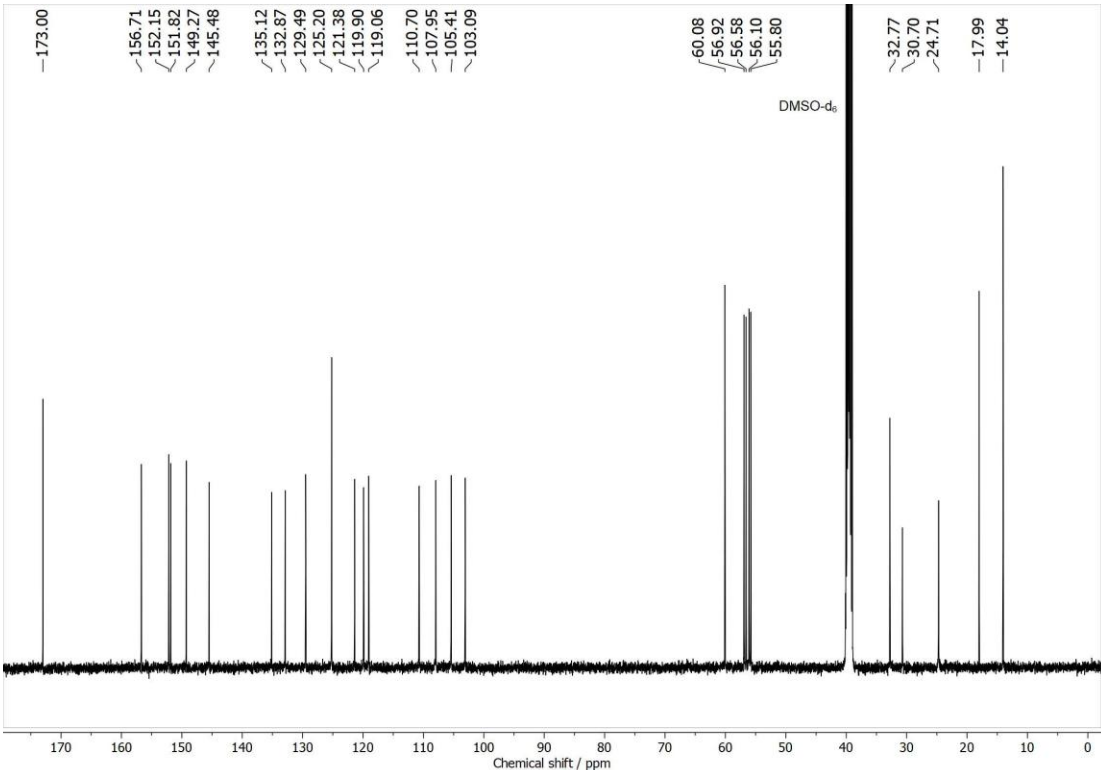

Figure S9. ^{13}C NMR spectrum (125 MHz, DMSO-d_6) of **3c-HBF$_4$**.

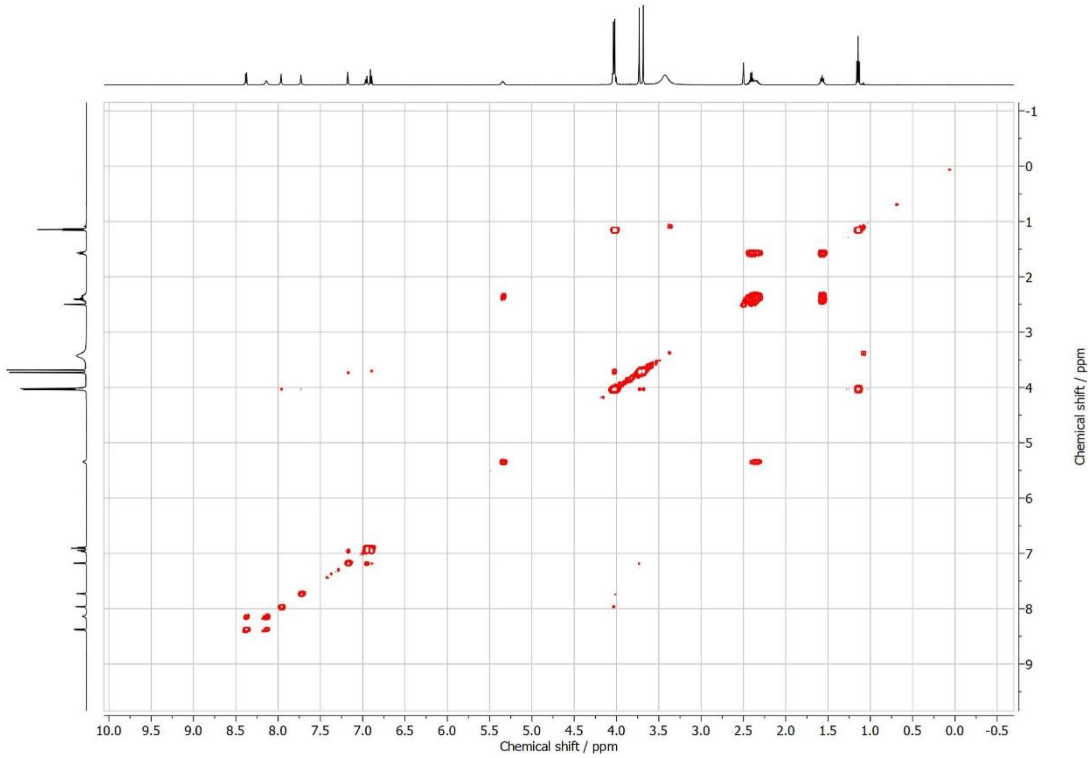

Figure S10. $^{1}H^{1}H$ COSY NMR spectrum (500 MHz, DMSO-d_6) of **3c-HBF$_4$**

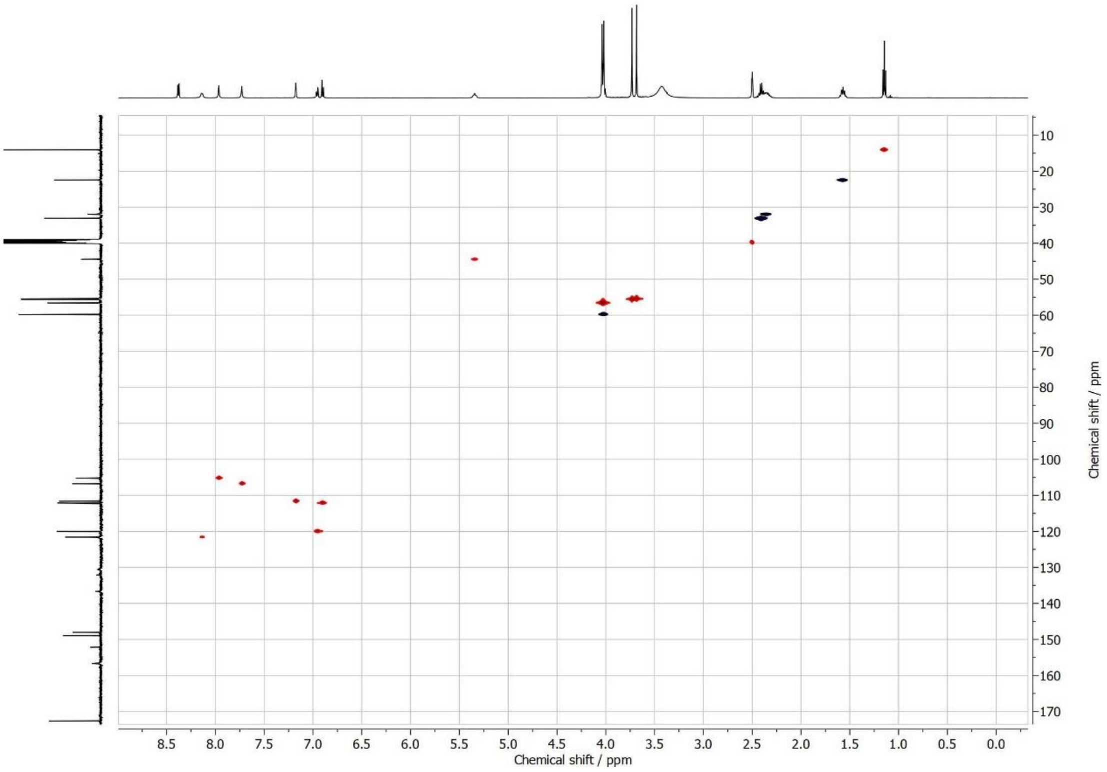

Figure S11. ^{1}H^{13}C HSQC NMR spectrum (500 MHz, DMSO-*d*$_6$) of **3c-HBF$_4$**.

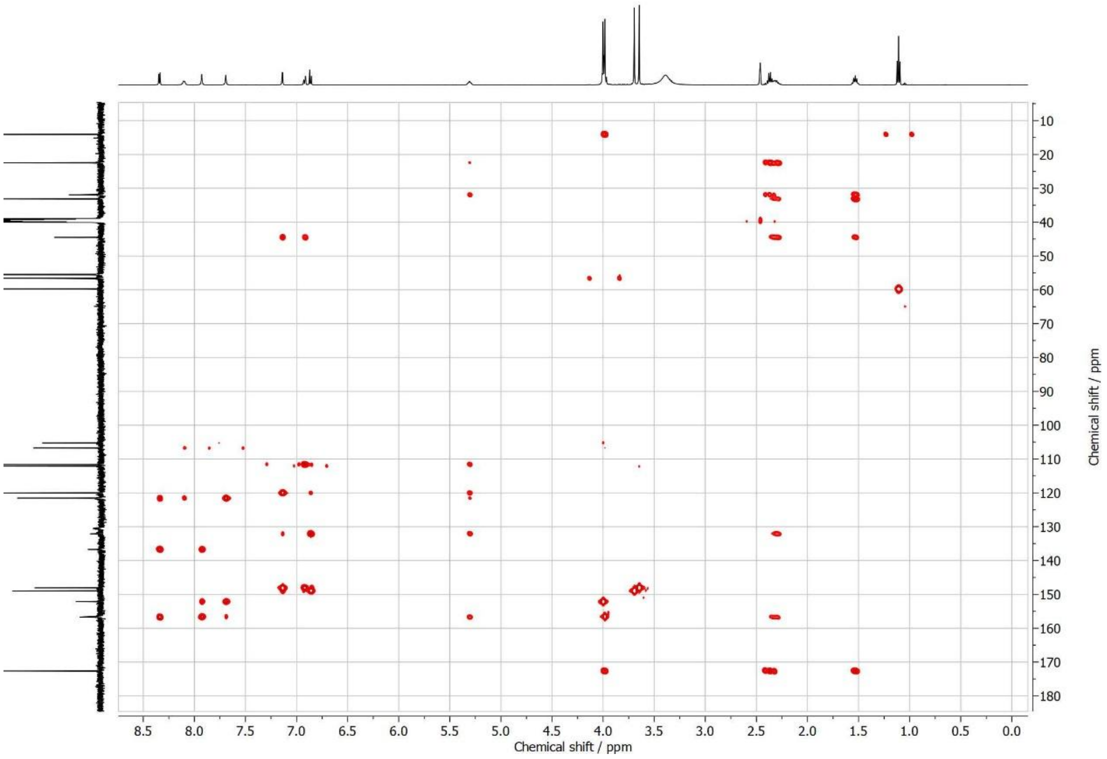

Figure S12. ^{1}H^{13}C HMBC NMR spectrum (500 MHz, DMSO-*d*$_6$) of **3c-HBF$_4$**.

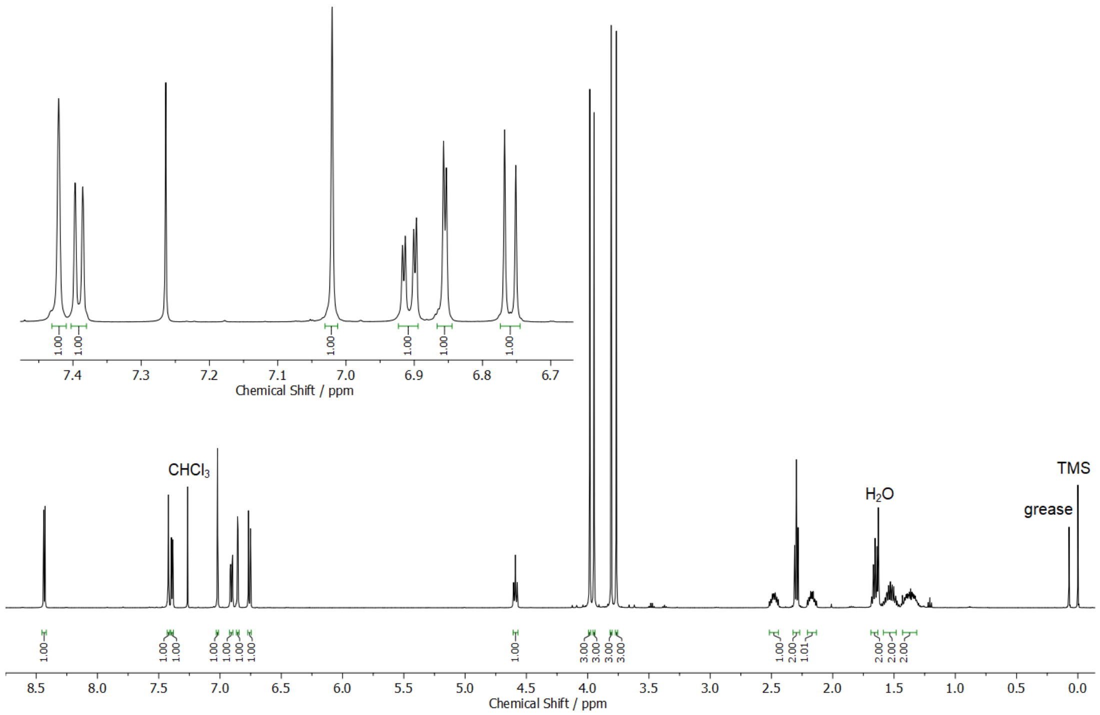

Figure S13. ^{1}H NMR spectrum (500 MHz, $CDCl_3$) of **3d**.

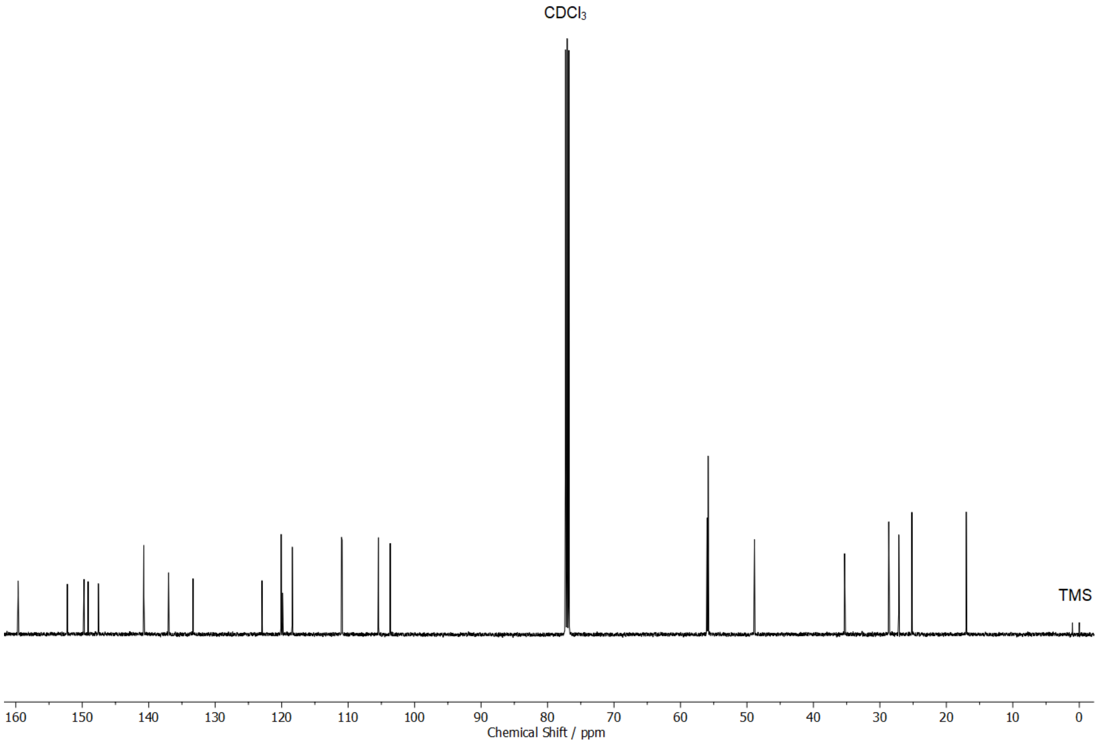

Figure S14. ^{13}C NMR spectrum (125 MHz, $CDCl_3$) of **3d**.

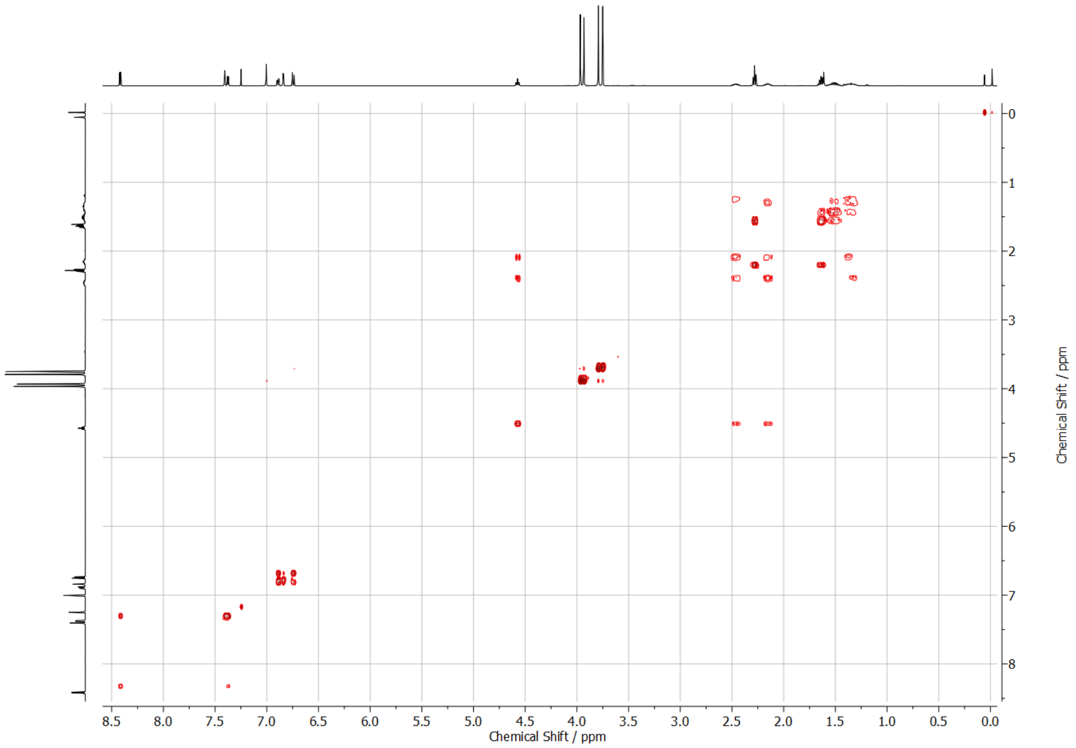

Figure S15. ^{1}H^{1}H COSY NMR spectrum (500 MHz, $CDCl_3$) of **3d**.

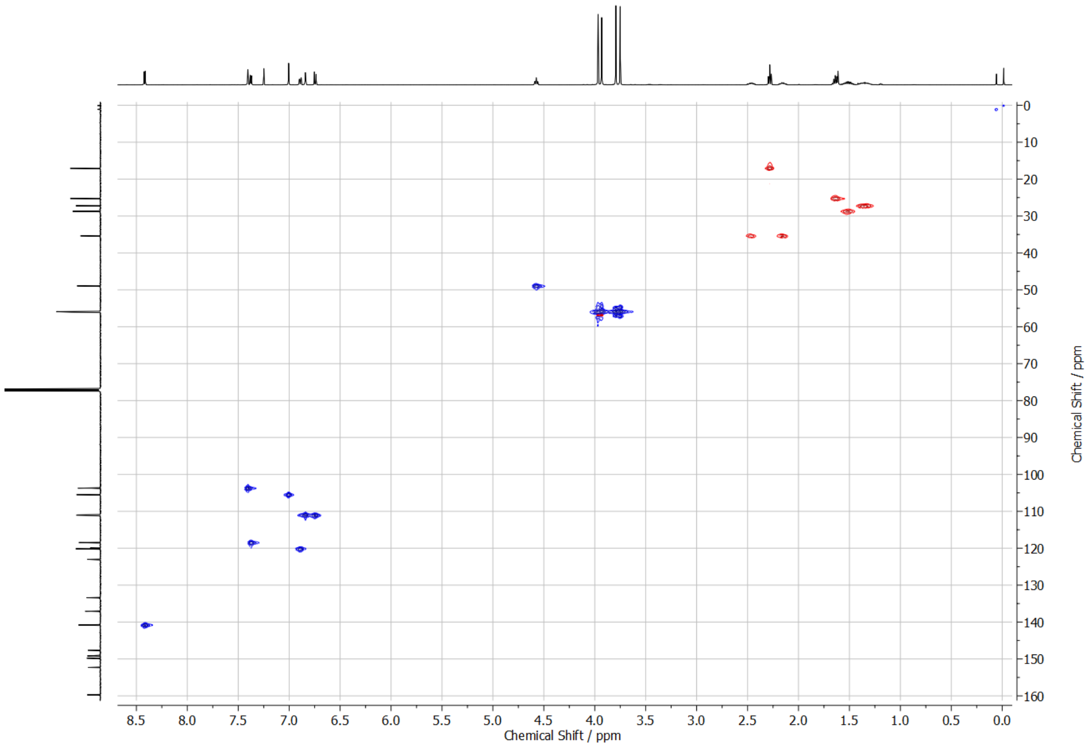

Figure S16. ^{1}H^{13}C HSQC NMR spectrum (500 MHz, $CDCl_3$) of **3d**.

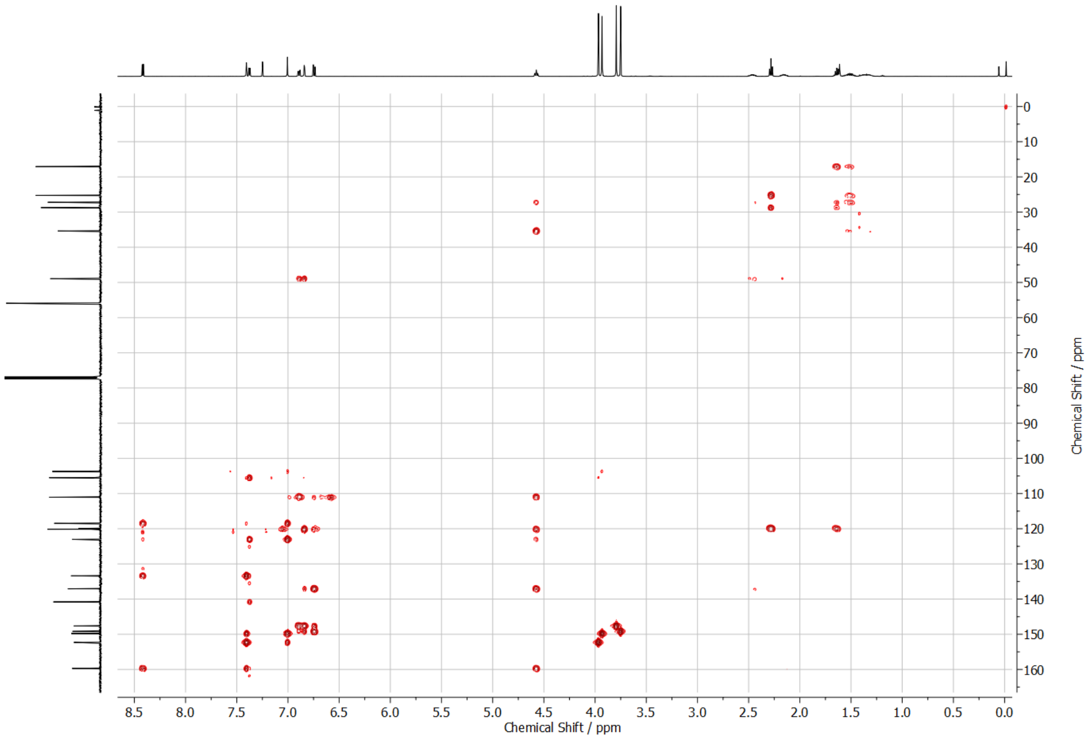

Figure S17. ^{1}H^{13}C HMBC NMR spectrum (500 MHz, $CDCl_3$) of **3d**.

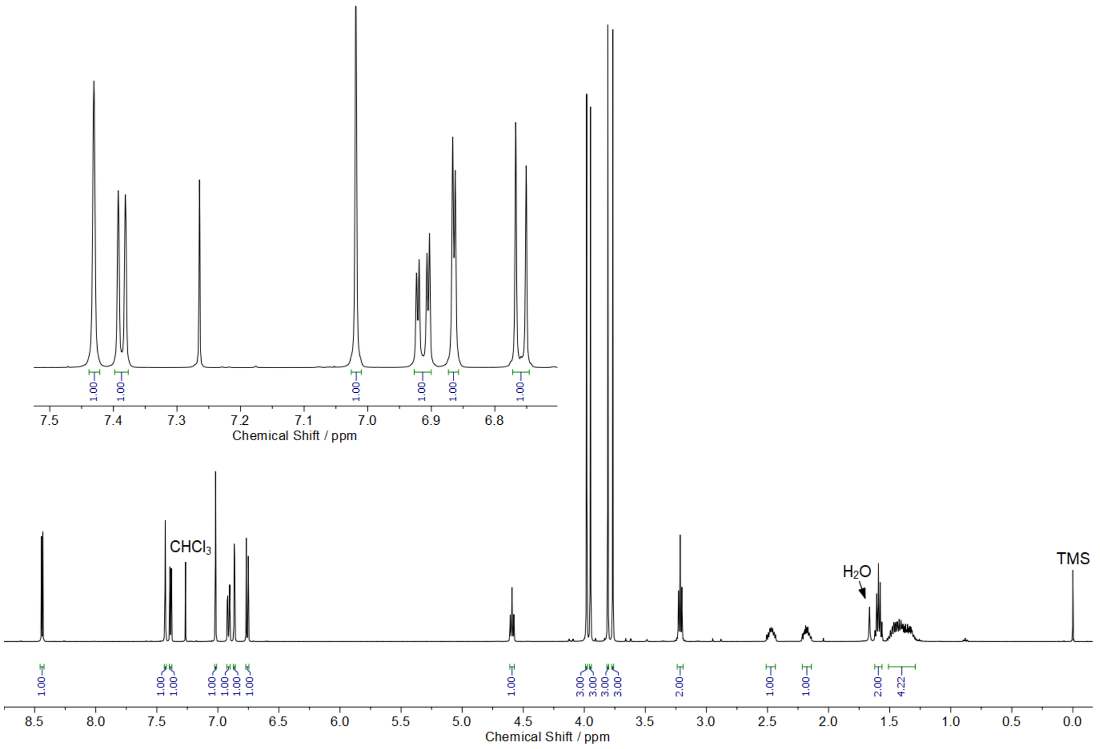

Figure S18. ^{1}H NMR spectrum (500 MHz, $CDCl_3$) of **3e**.

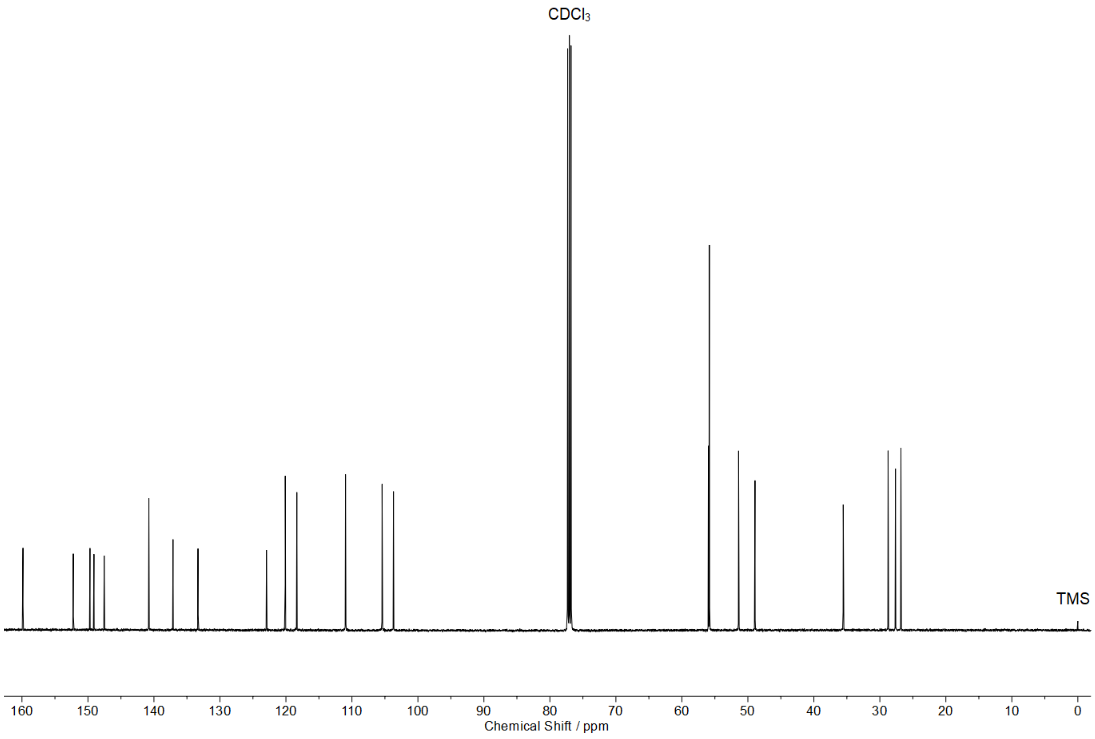

Figure S19. ^{13}C NMR spectrum (125 MHz, $CDCl_3$) of **3e**.

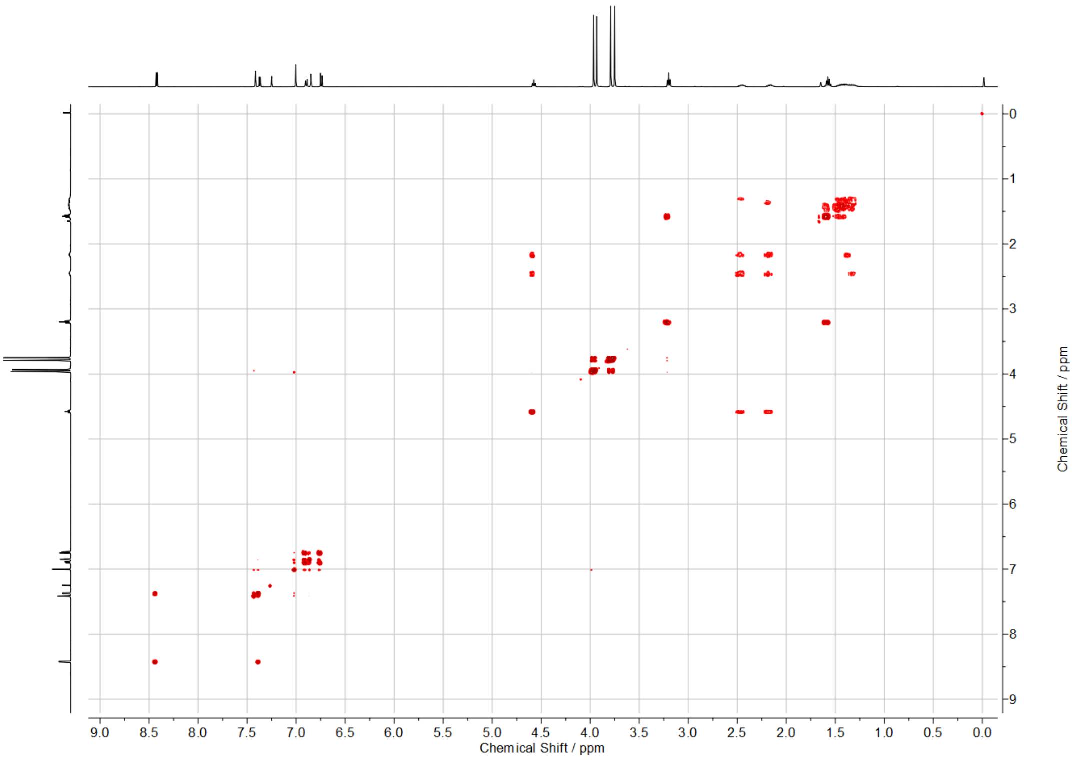

Figure S20. $^{1}H^{1}H$ COSY NMR spectrum (500 MHz, $CDCl_3$) of **3e**.

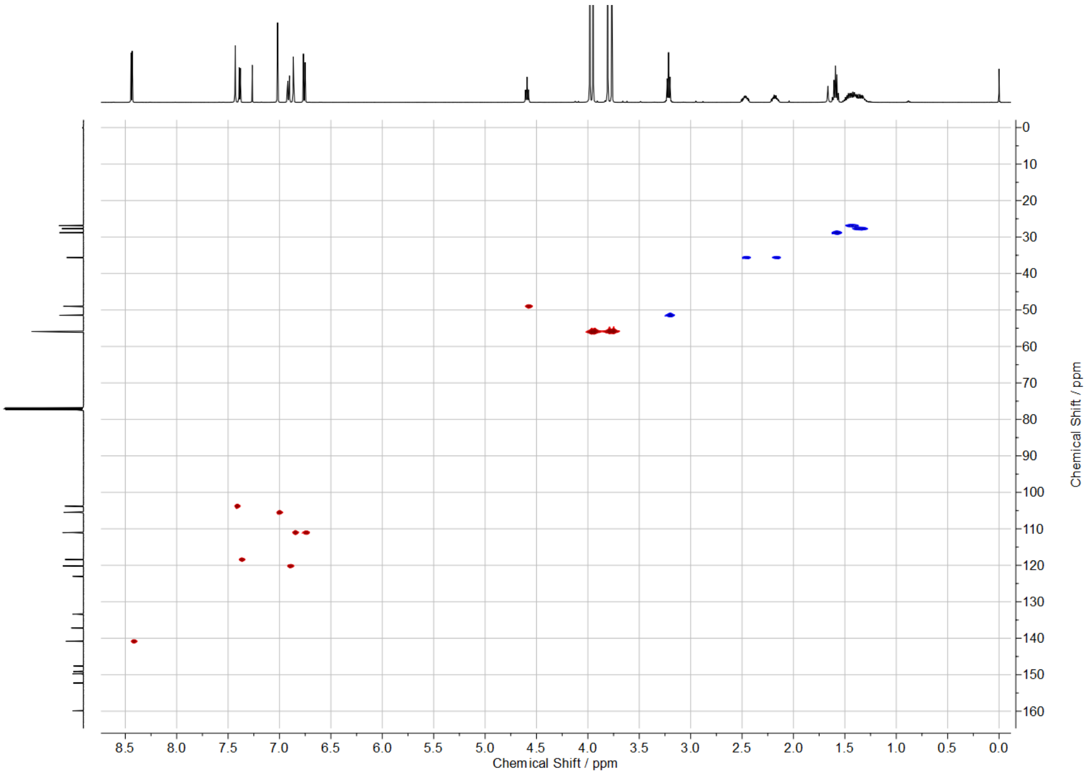

Figure S21. $^{1}H^{13}C$ HSQC NMR spectrum (500 MHz, $CDCl_3$) of **3e**.

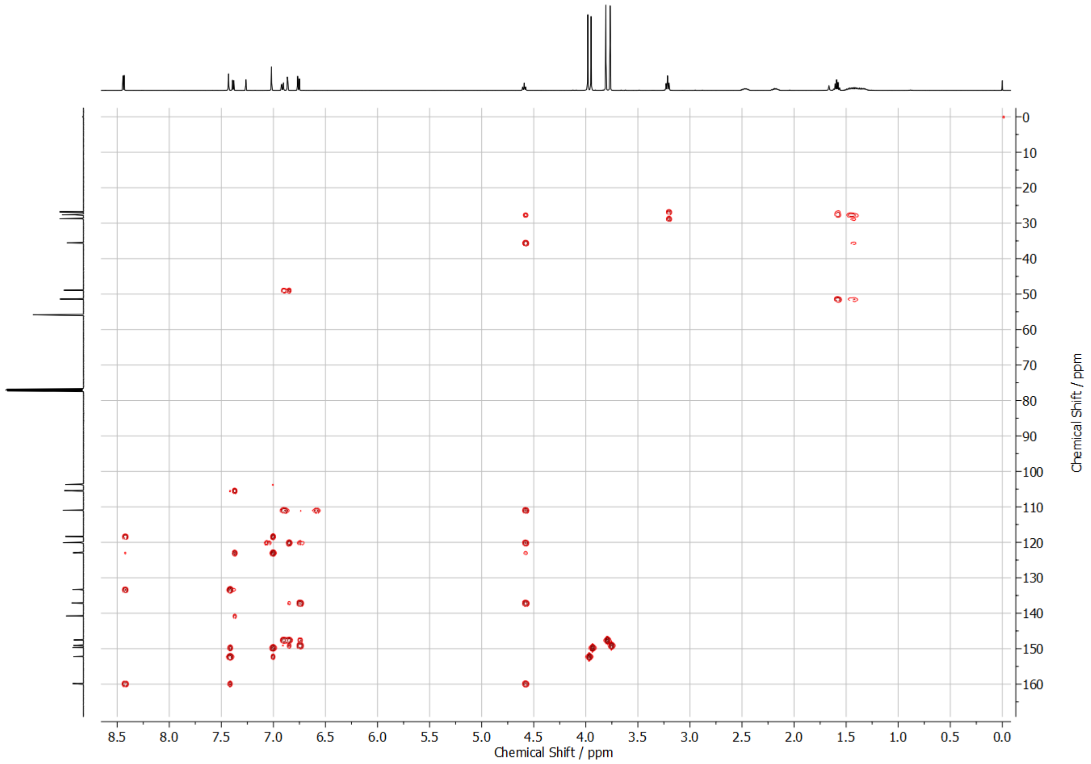

Figure S22. $^{1}H^{13}C$ HMBC NMR spectrum (500 MHz, $CDCl_3$) of **3e**.

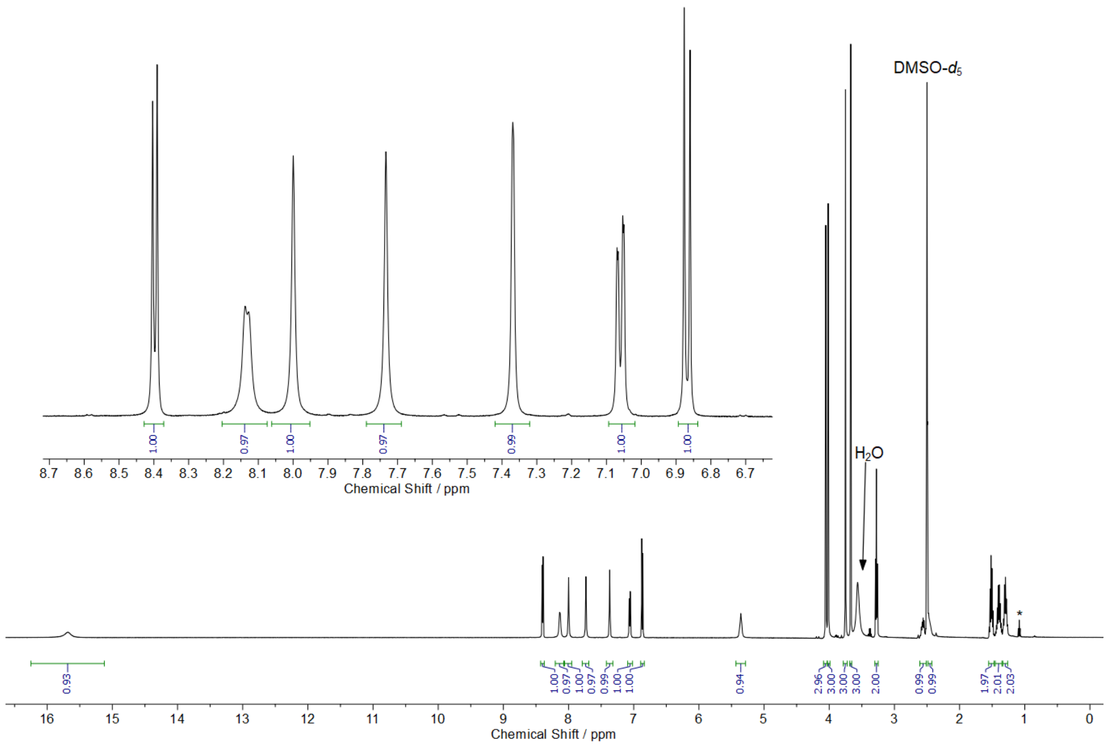

Figure S23. ^{1}H NMR spectrum (500 MHz, DMSO-d_6) of **3e-HCl**.

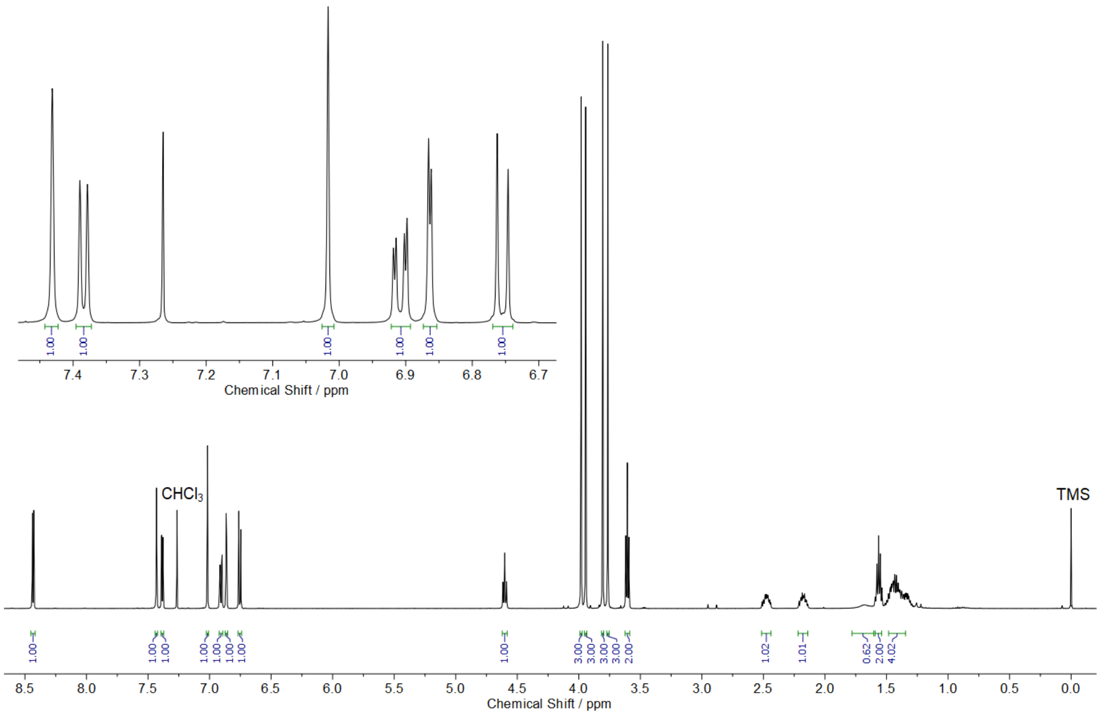

Figure S24. ^{1}H NMR spectrum (500 MHz, CDCl$_3$) of **3f**.

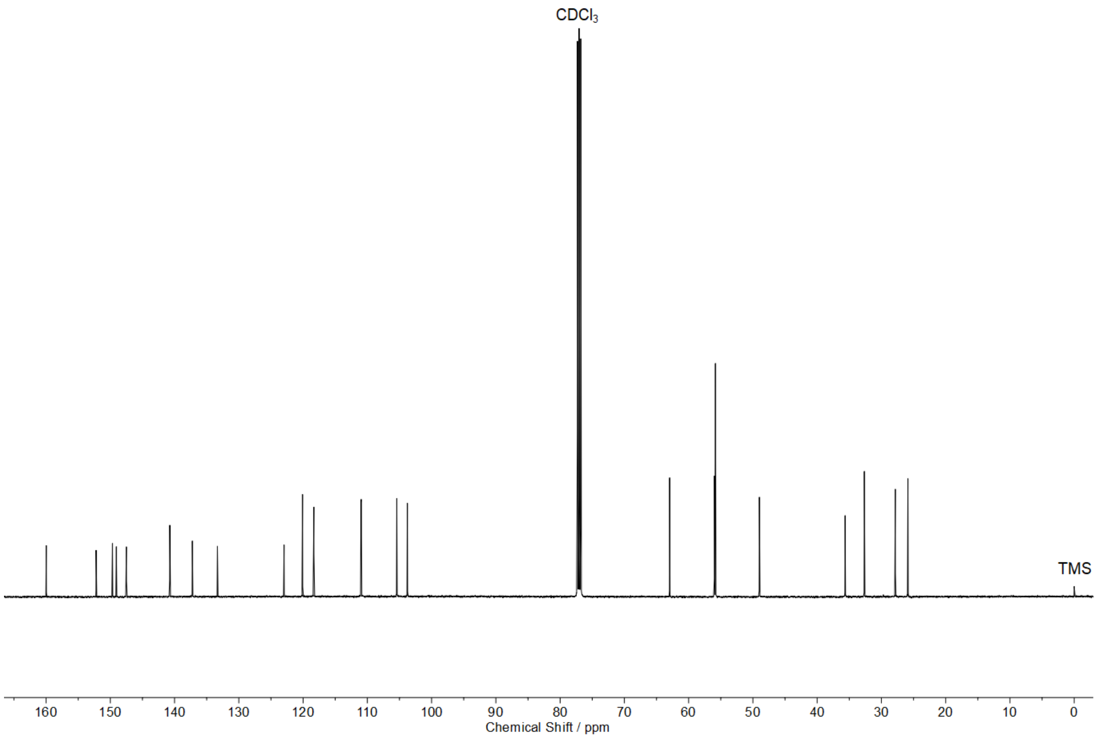

Figure S25. ^{13}C NMR spectrum (125 MHz, $CDCl_3$) of **3f**.

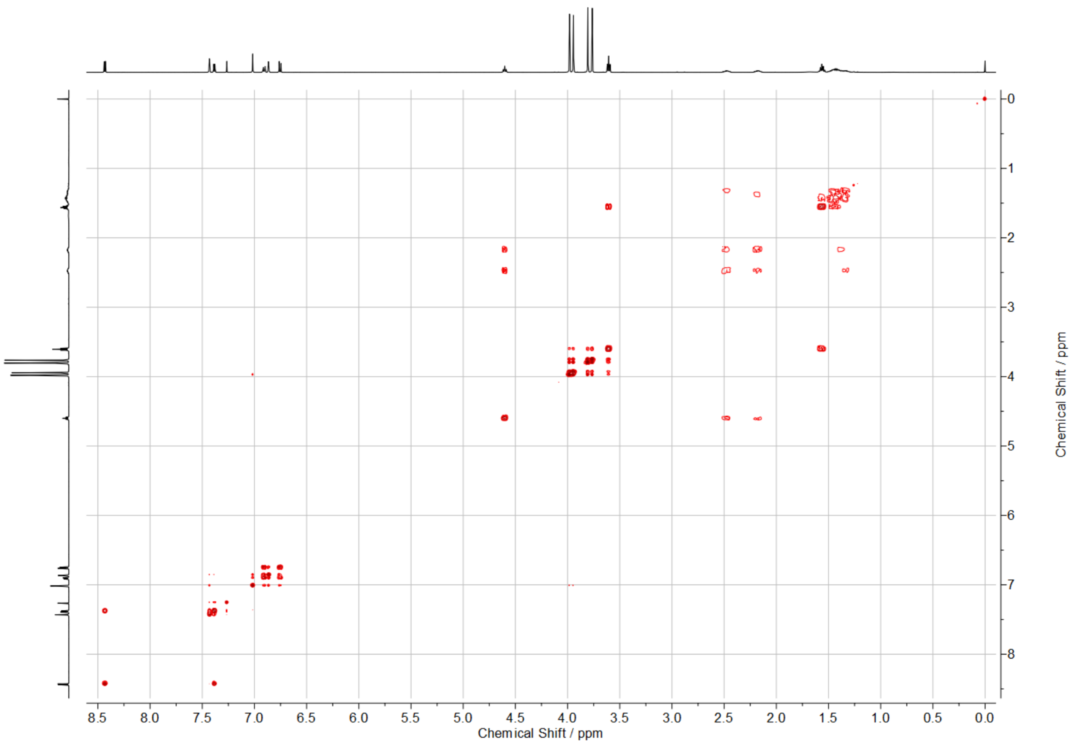

Figure S26. $^1H^1H$ COSY NMR spectrum (500 MHz, $CDCl_3$) of **3f**.

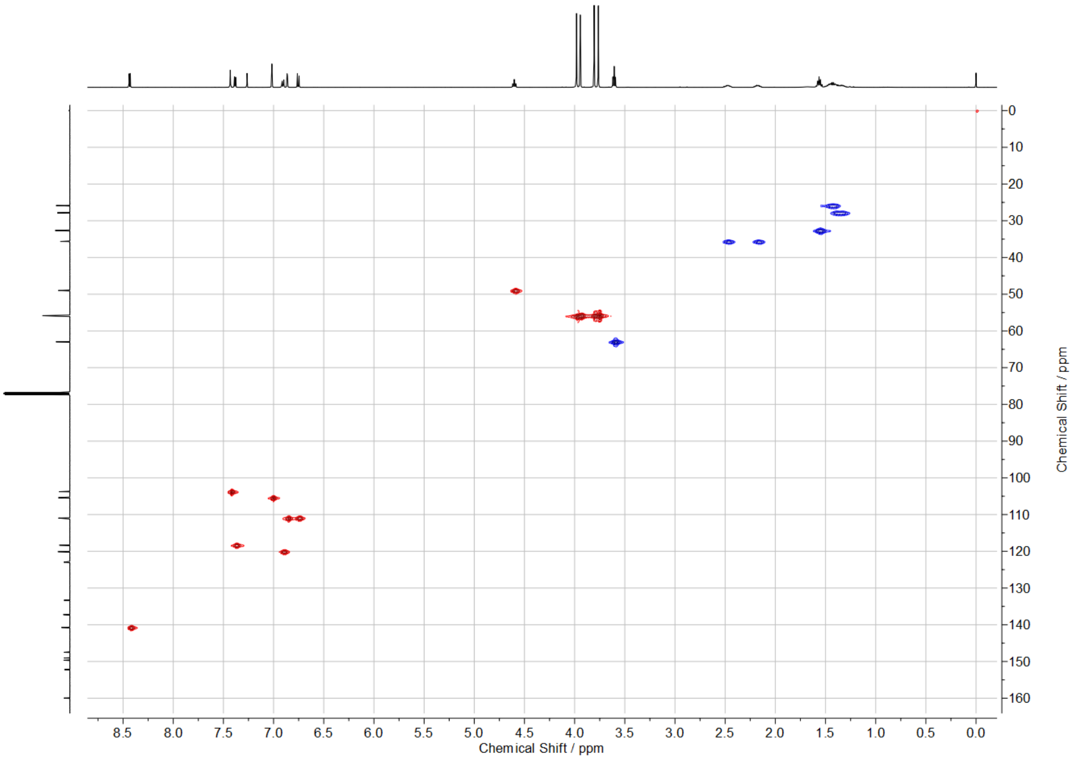

Figure S27. $^{1}H^{13}C$ HSQC NMR spectrum (500 MHz, $CDCl_3$) of **3f**.

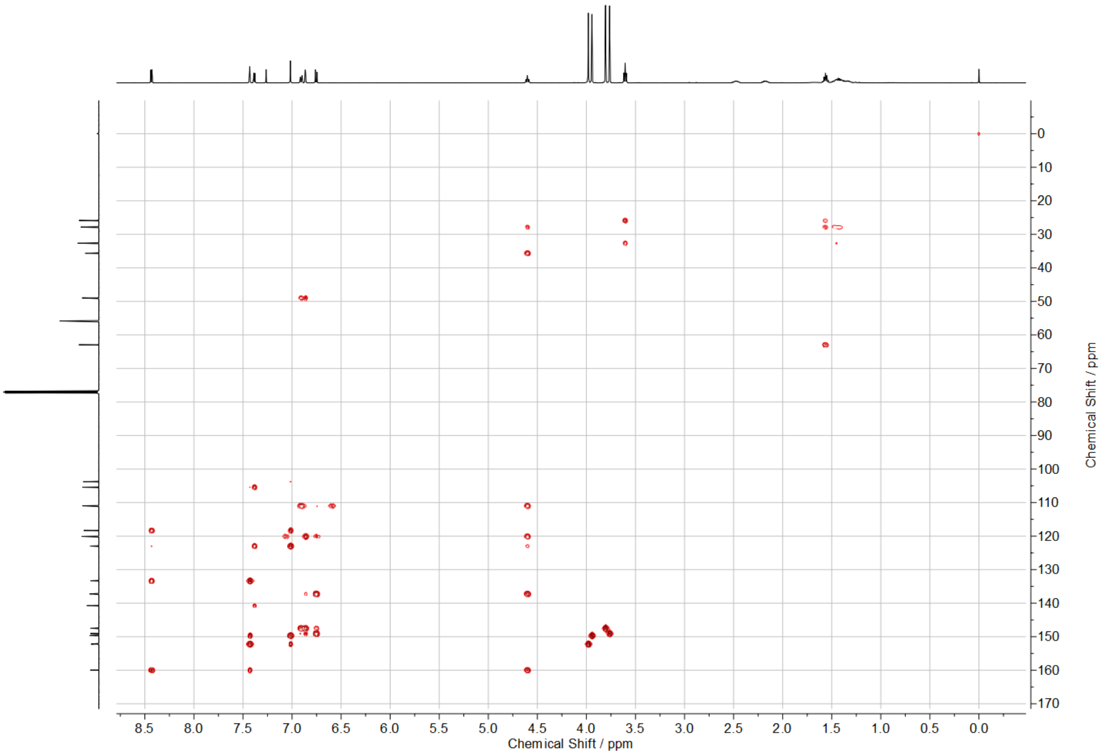

Figure S28. $^{1}H^{13}C$ HMBC NMR spectrum (500 MHz, $CDCl_3$) of **3f**.

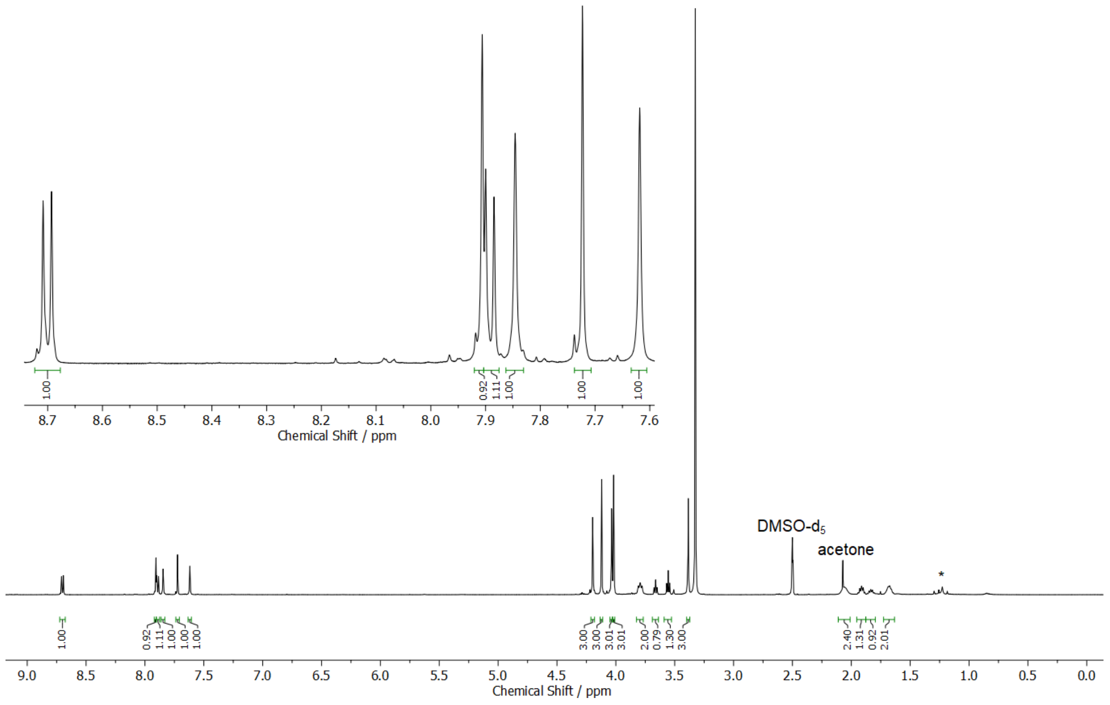

Figure S29. ^{1}H NMR spectrum (500 MHz, DMSO-*d*6) of **2b**.

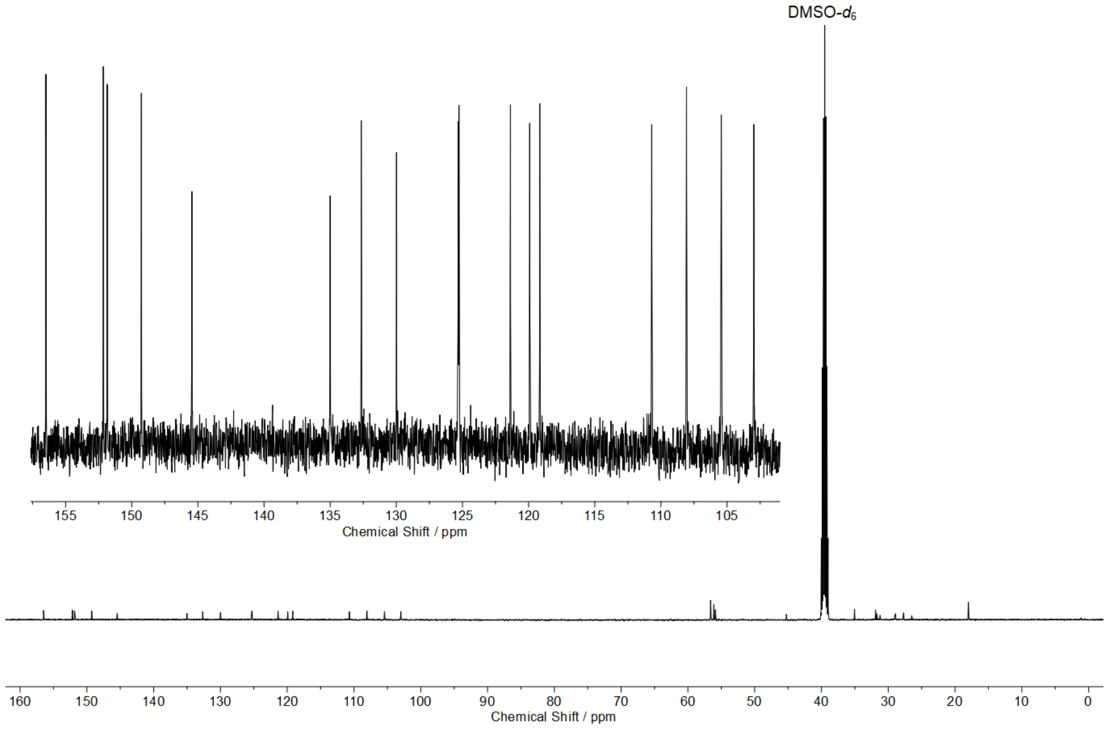

Figure S30. ^{13}C NMR spectrum (125 MHz, DMSO-*d*6) of **2b**.

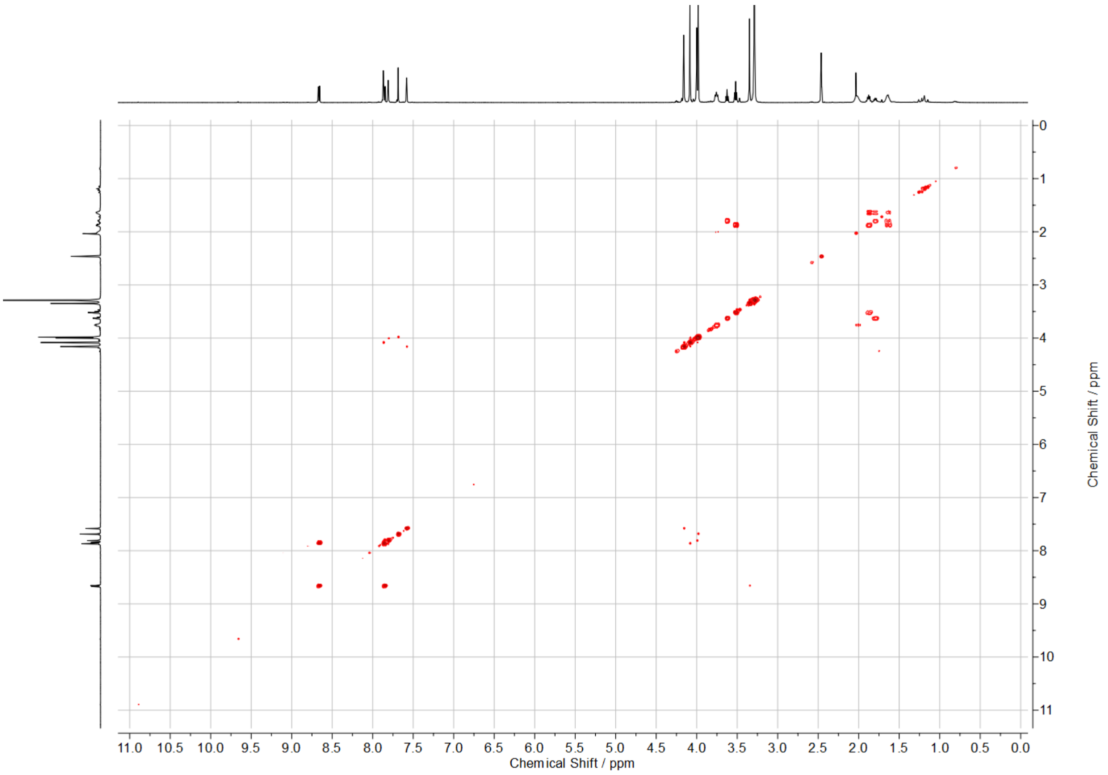

Figure S31. $^{1}H^{1}H$ COSY NMR spectrum (500 MHz, DMSO-*d*$_6$) of **2b**.

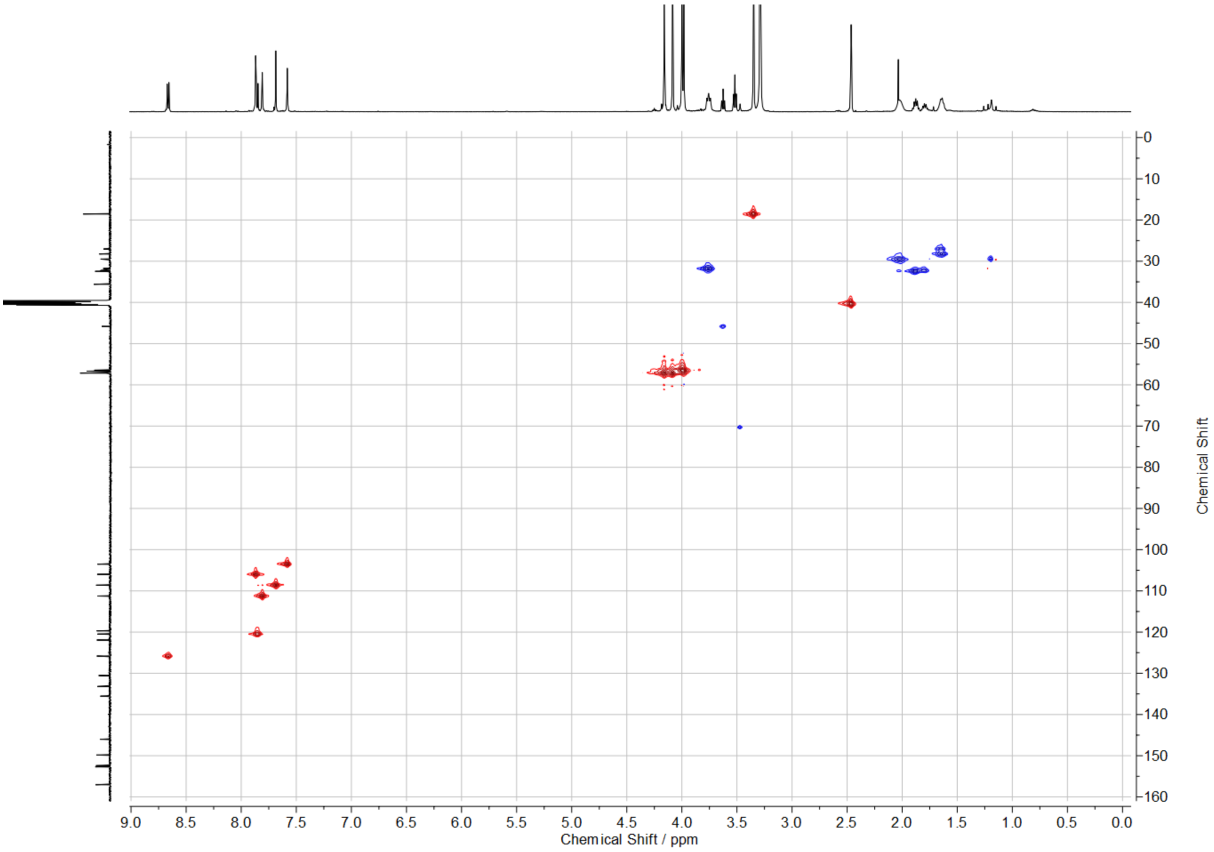

Figure S32. $^{1}H^{13}C$ HSQC NMR spectrum (500 MHz, DMSO-*d*$_6$) of **2b**.

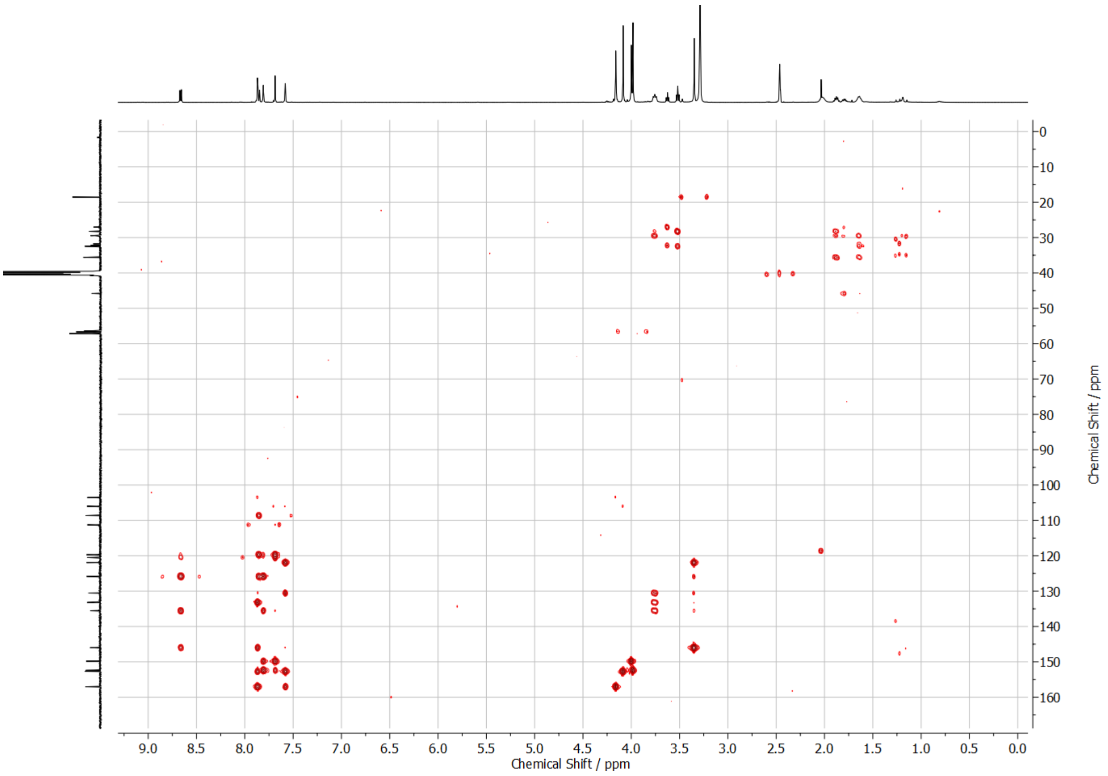

Figure S33. $^{1}H^{13}C$ HMBC NMR spectrum (500 MHz, DMSO-d_6) of **2b**.

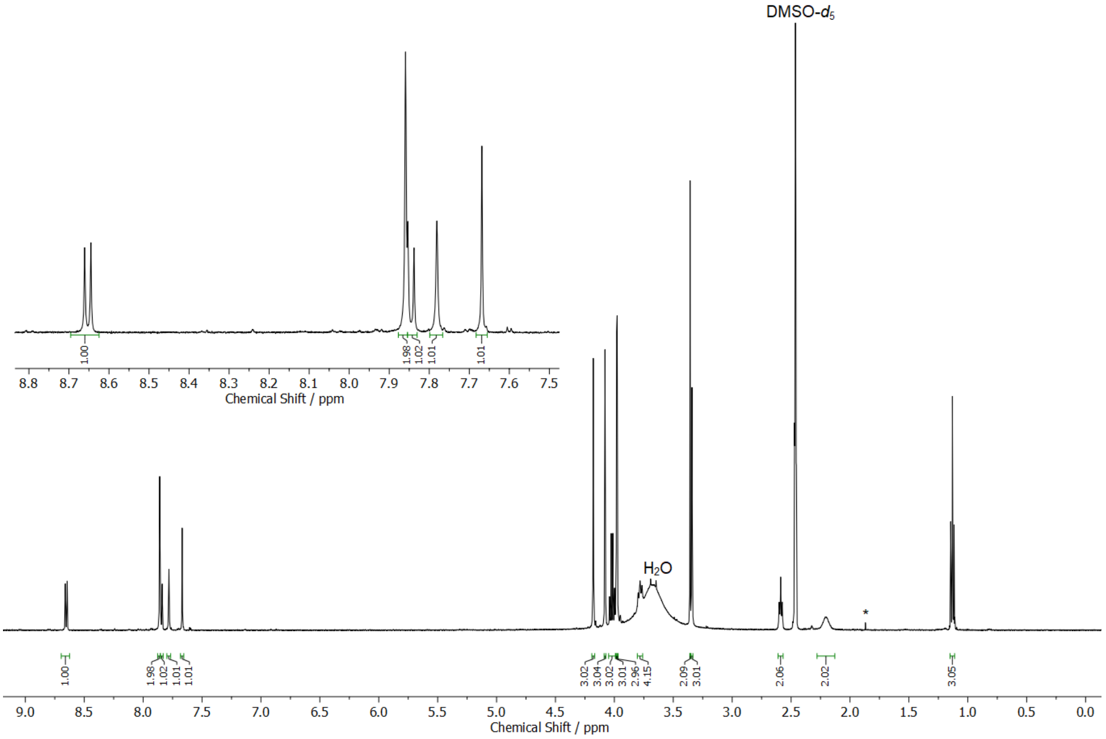

Figure S34. ^{1}H NMR spectrum (500 MHz, DMSO-d_6) of **2c** sulfoacetate salt.

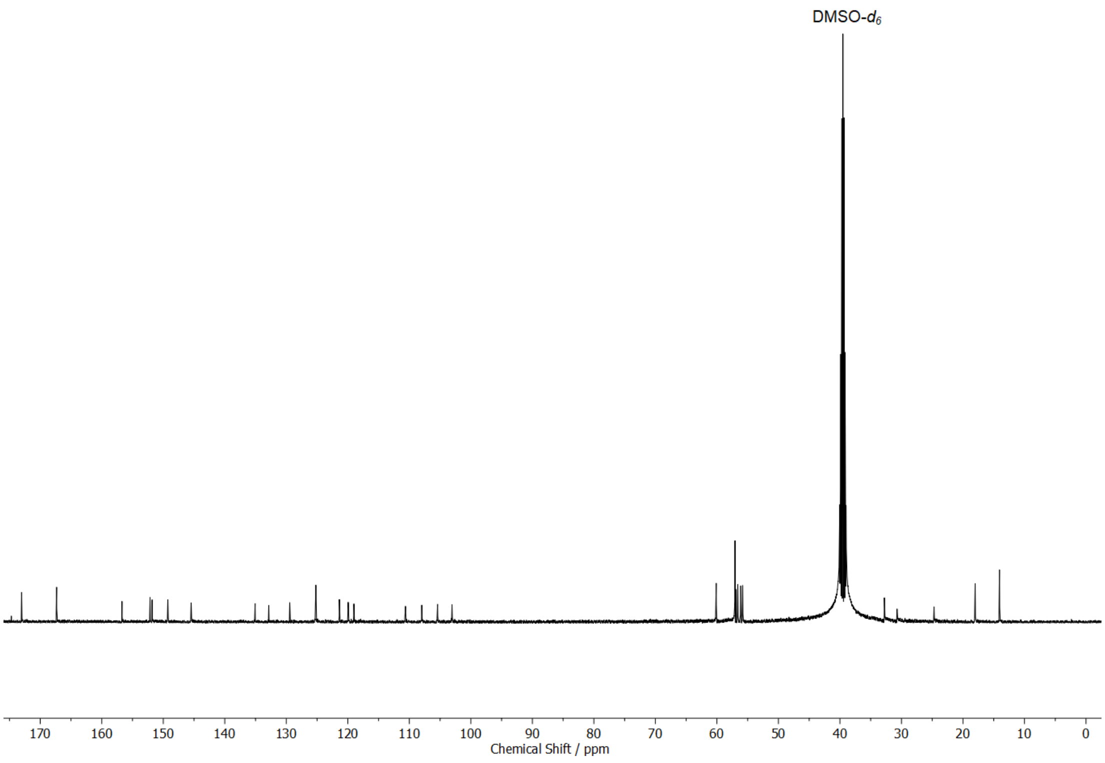

Figure S35. ^{13}C NMR spectrum (125 MHz, DMSO-d_6) of **2c** sulfoacetate salt.

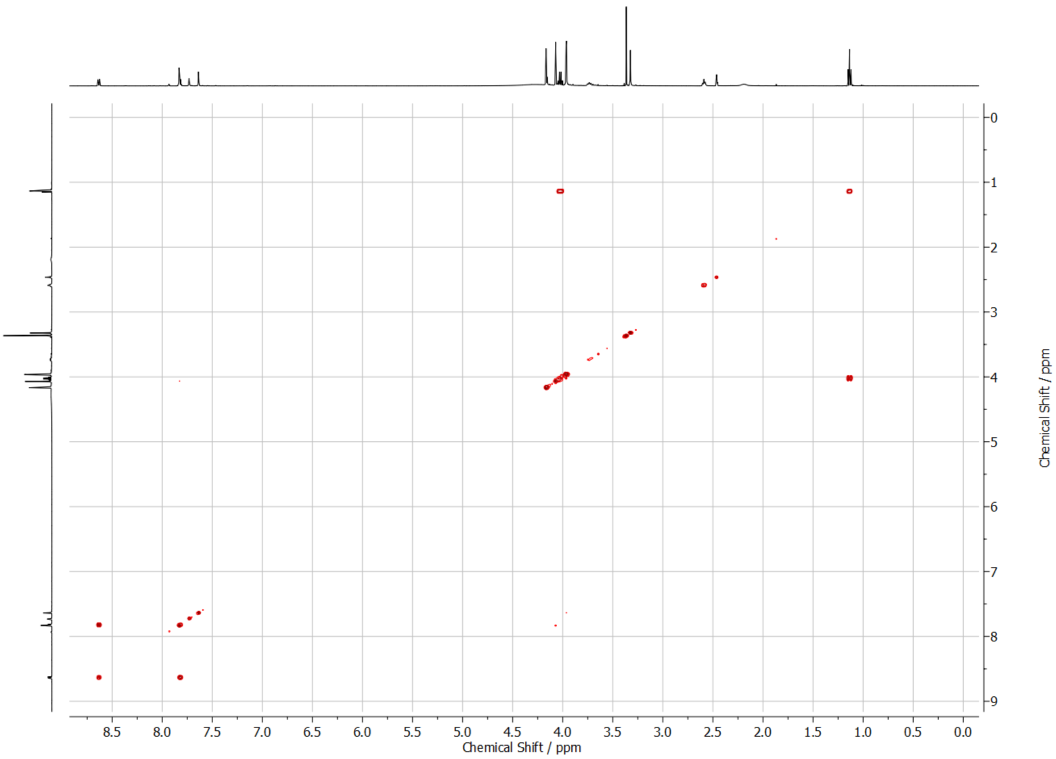

Figure S36. ^{1}H^{1}H COSY NMR spectrum (500 MHz, DMSO-d_6) of **2c** sulfoacetate salt.

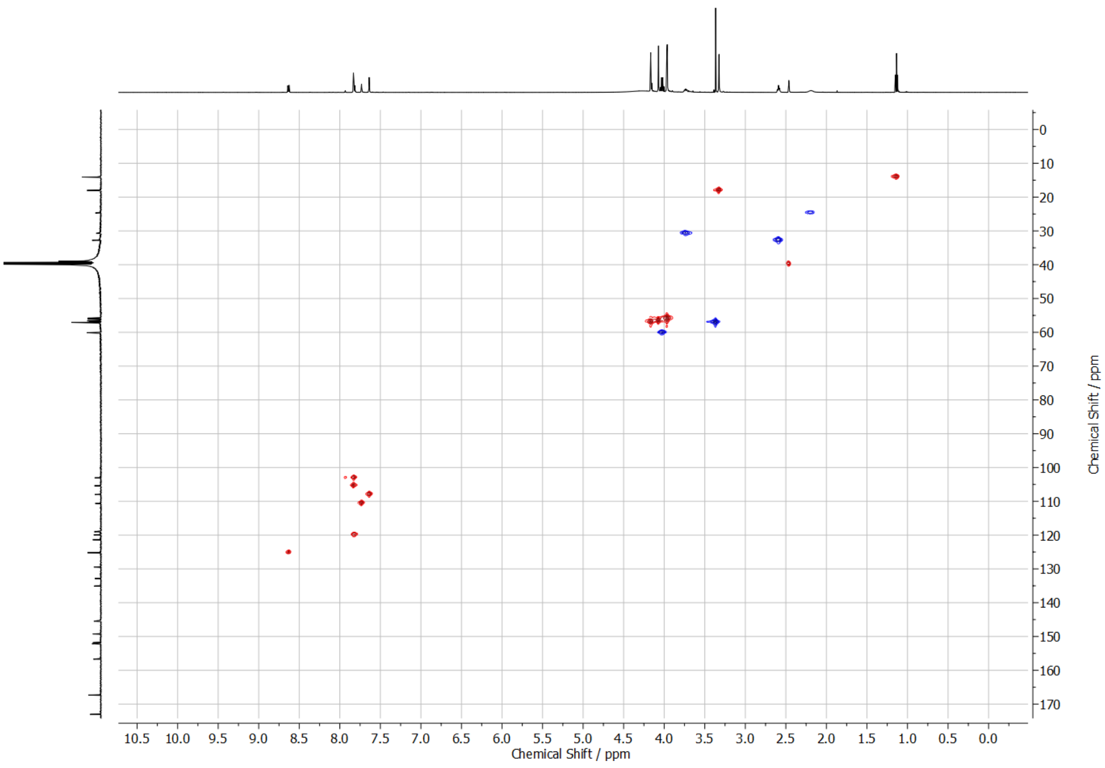

Figure S37. $^{1}H^{13}C$ HSQC NMR spectrum (500 MHz, DMSO-d_6) of **2c** sulfoacetate salt.

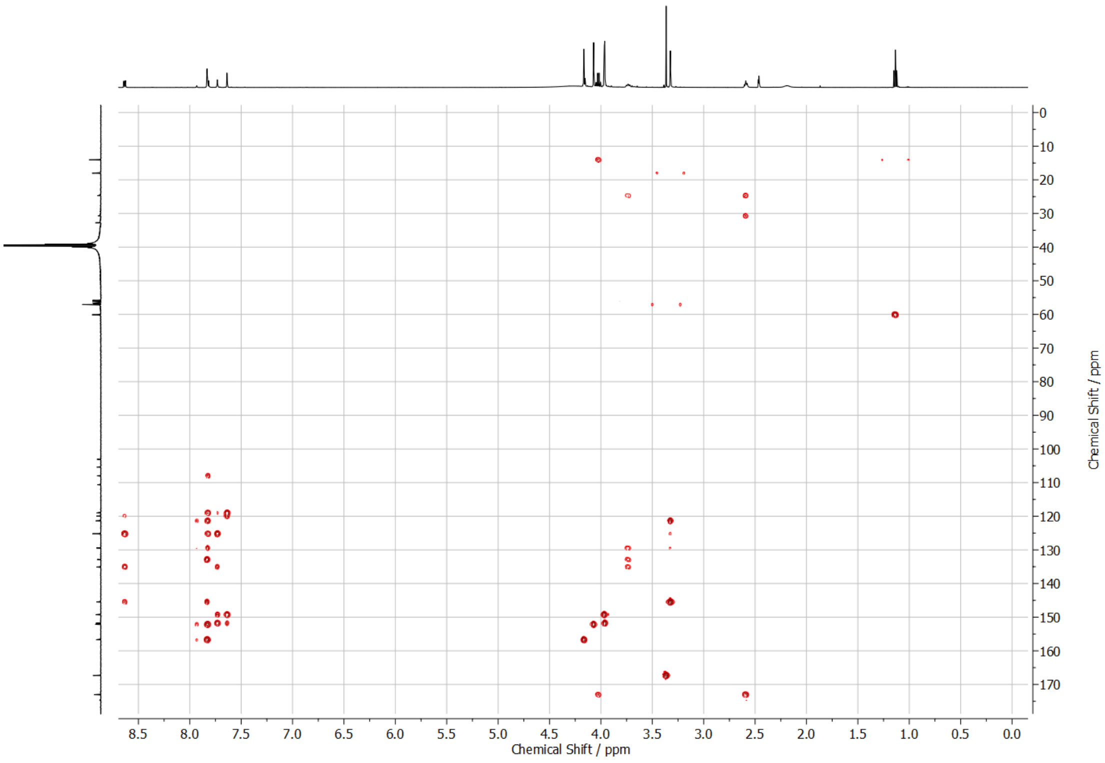

Figure S38. $^{1}H^{13}C$ HMBC NMR spectrum (500 MHz, DMSO-d_6) of **2c** sulfoacetate salt.

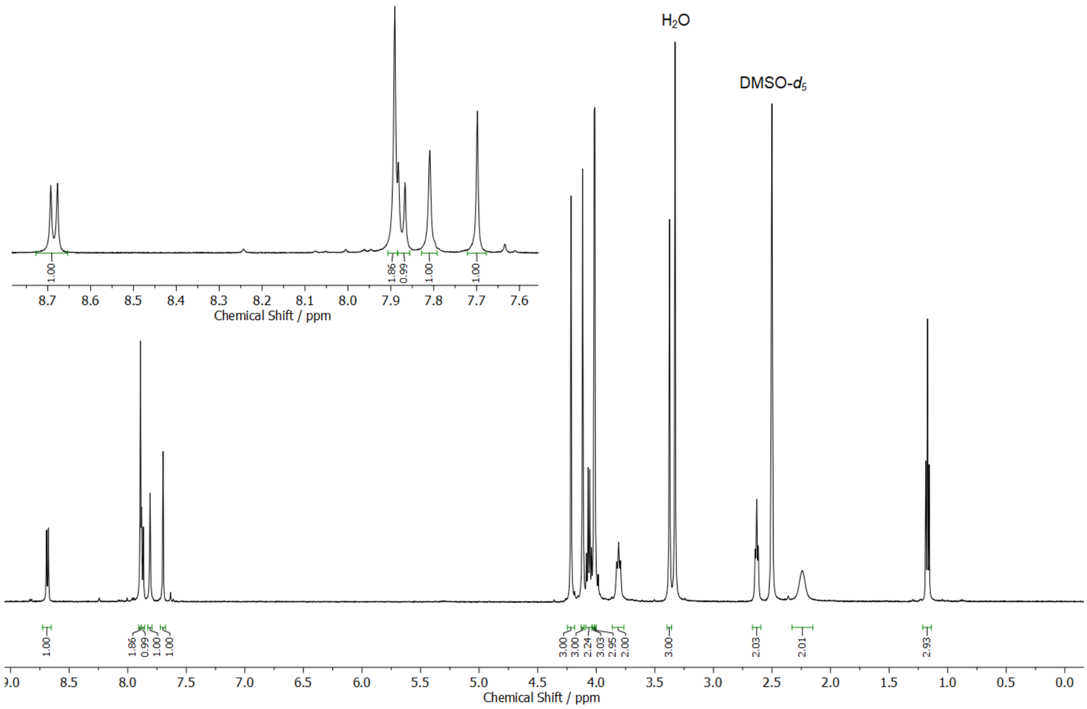

Figure S39. ^{1}H NMR spectrum (500 MHz, DMSO-d_6) of **2c**.

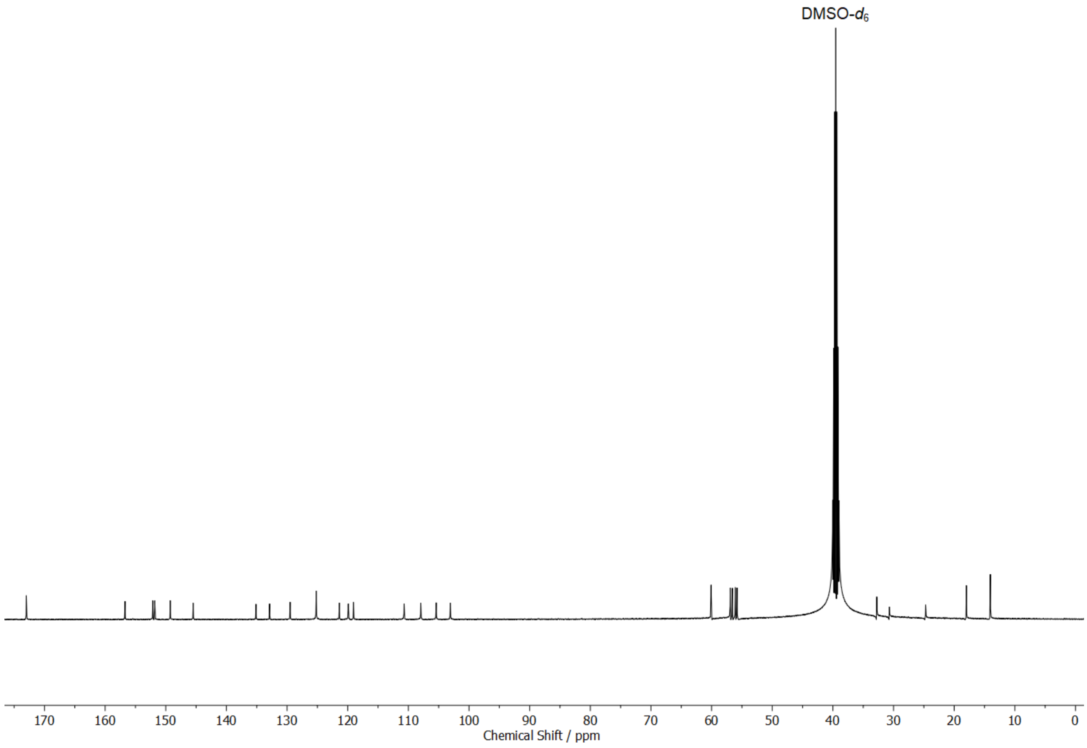

Figure S40. ^{13}C NMR spectrum (125 MHz, DMSO-d_6) of **2c**.

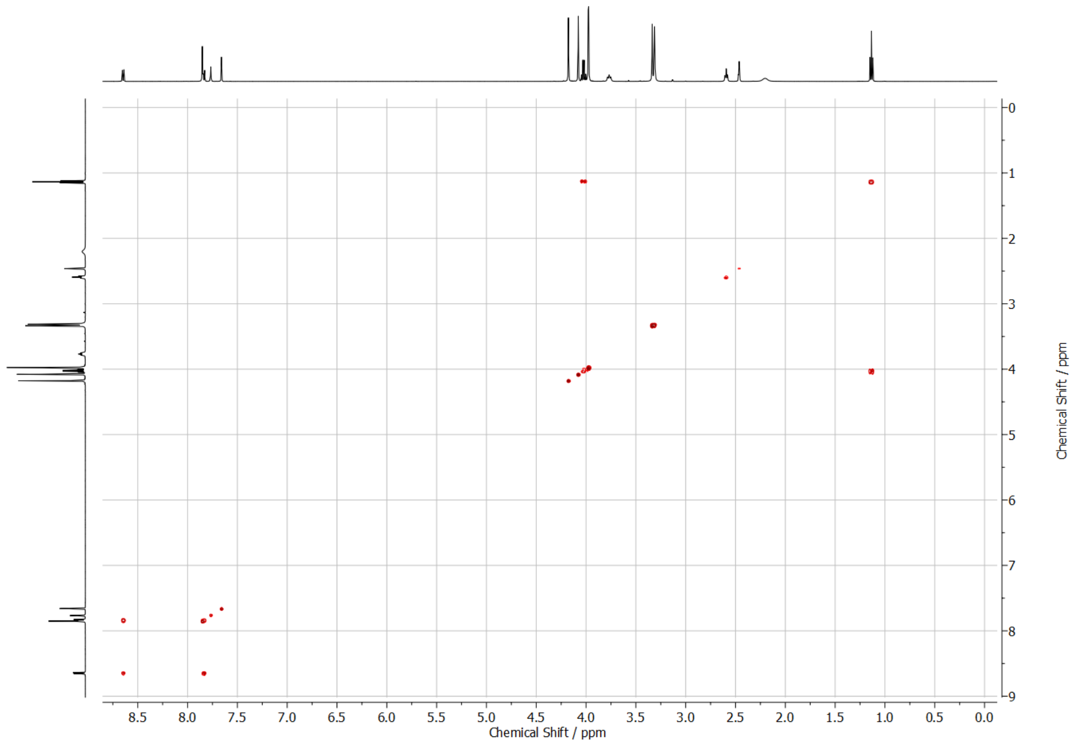

Figure S41. $^{1}H^{1}H$ COSY NMR spectrum (500 MHz, DMSO-d_6) of **2c**.

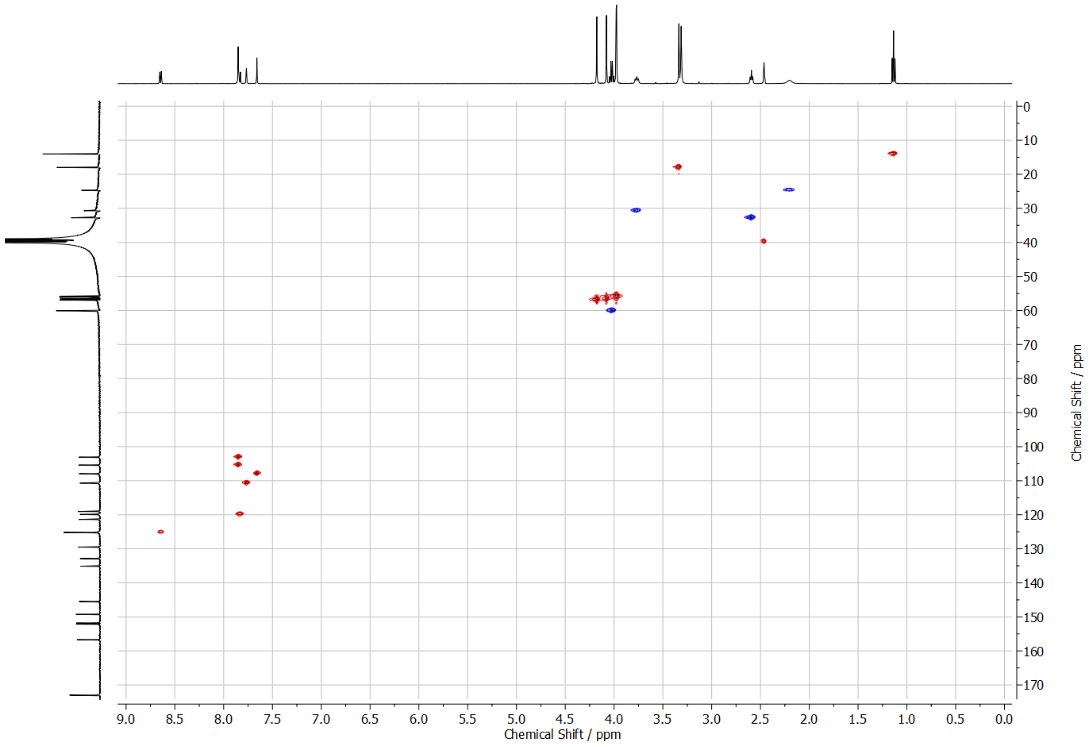

Figure S42. $^{1}H^{13}C$ HSQC NMR spectrum (500 MHz, DMSO-d_6) of **2c**.

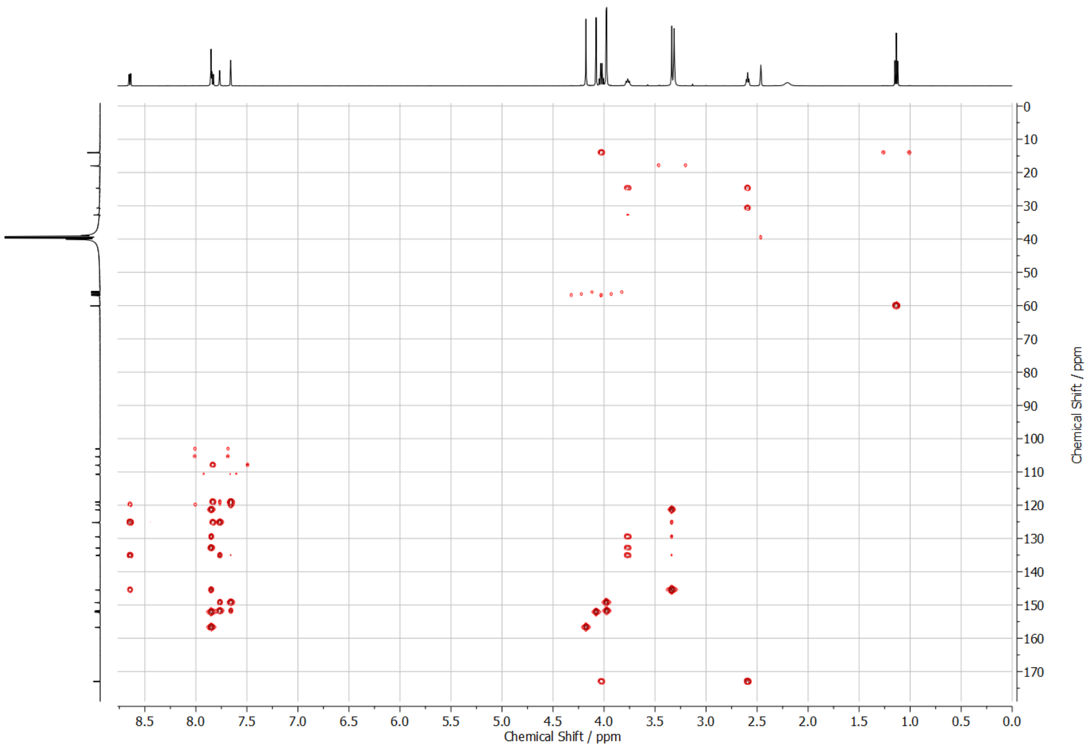

Figure S43. $^{1}H^{13}C$ HMBC NMR spectrum (500 MHz, DMSO-d_6) of **2c**.

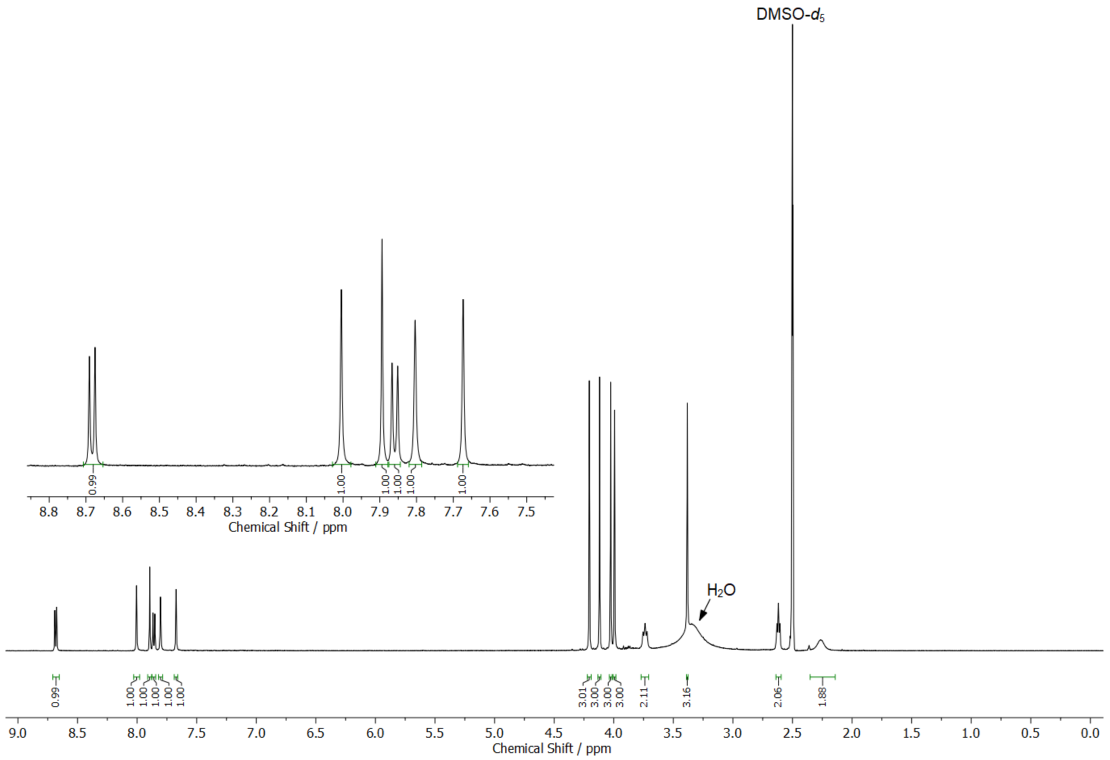

Figure S44. ^{1}H NMR spectrum (500 MHz, DMSO-d_6) of **2d**.

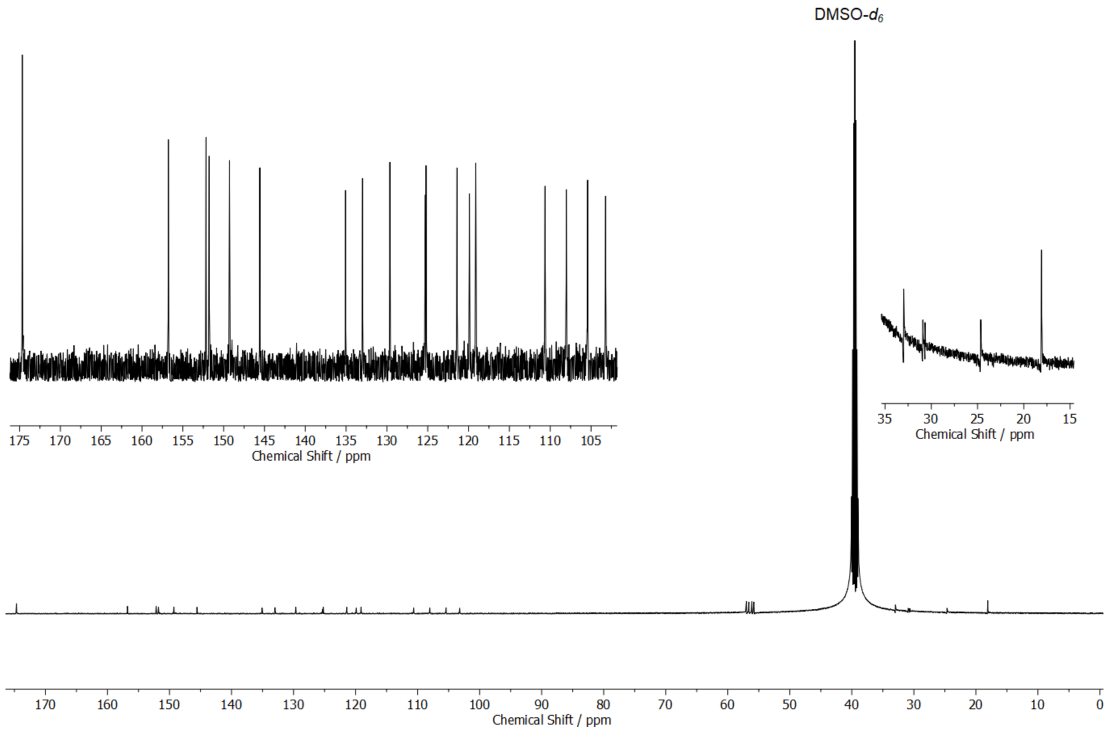

Figure S45. ^{13}C NMR spectrum (125 MHz, DMSO-d_6) of **2d**.

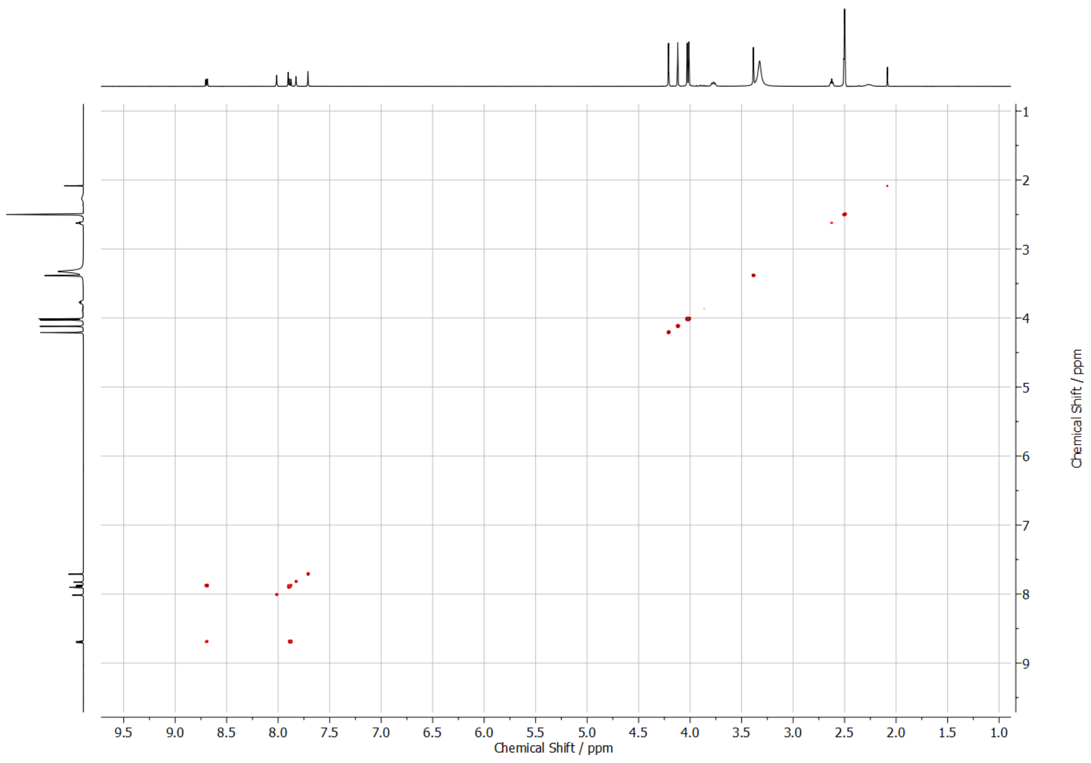

Figure S46. ^{1}H^{1}H COSY NMR spectrum (500 MHz, DMSO-d_6) of **2d**.

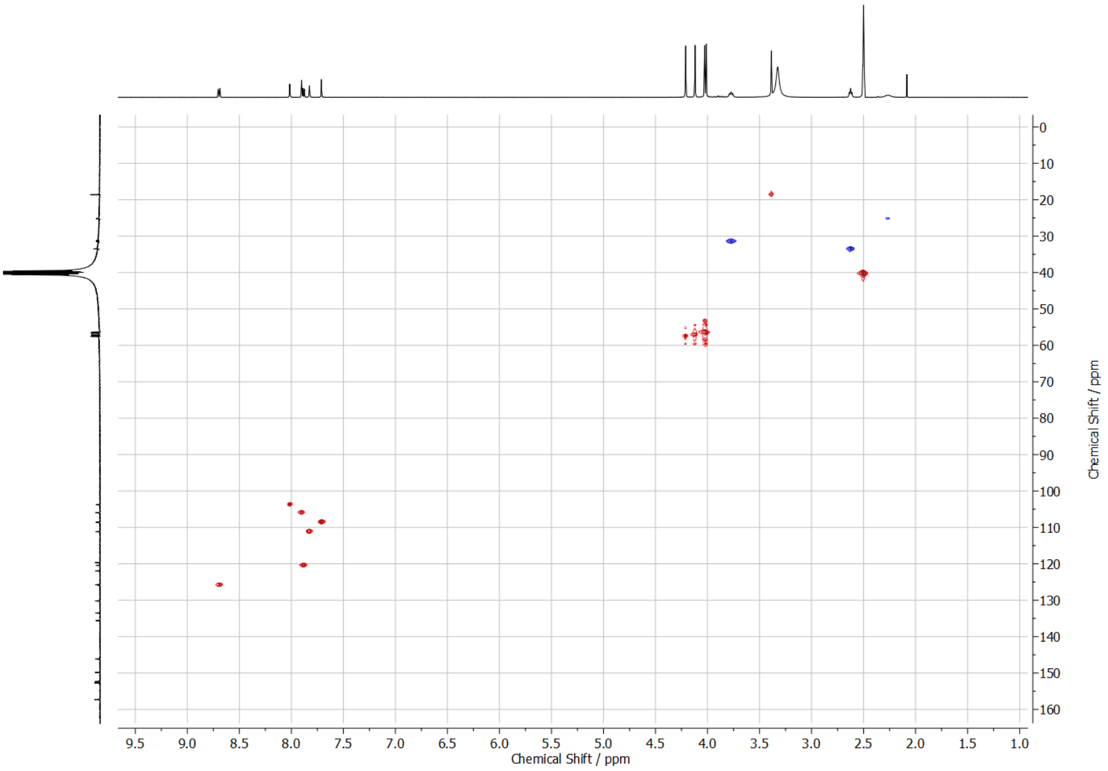

Figure S47. $^{1}H^{13}C$ HSQC NMR spectrum (500 MHz, DMSO-d_6) of **2d**.

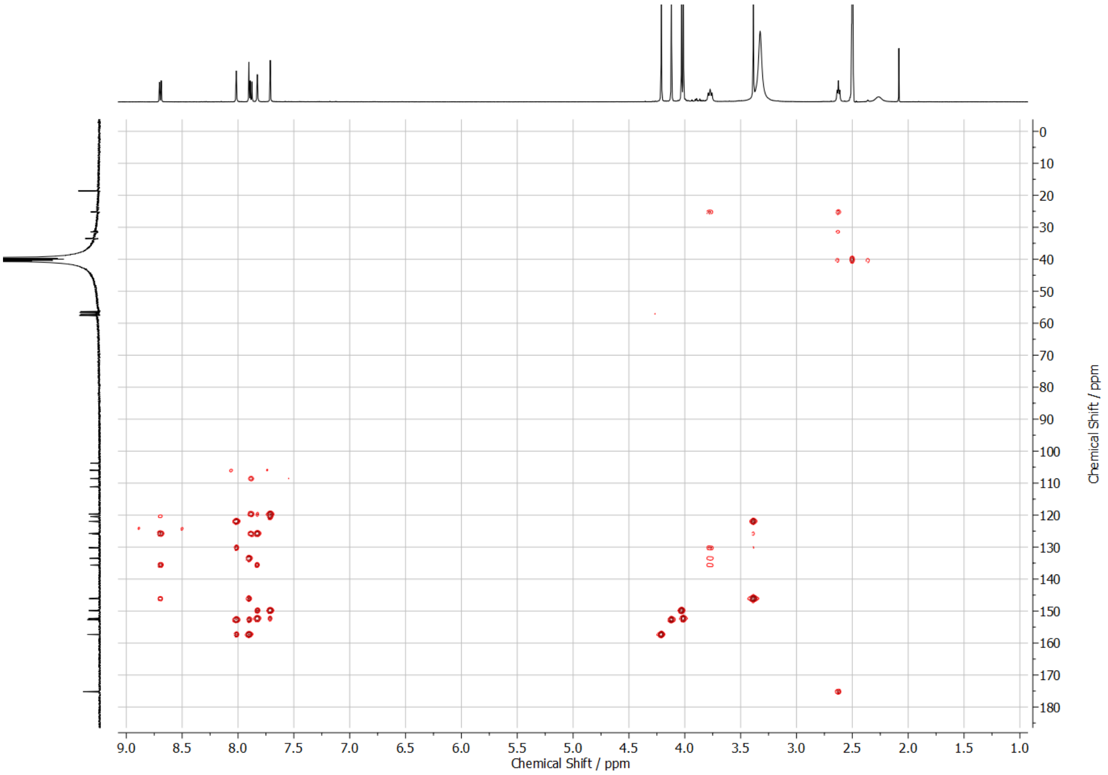

Figure S48. $^{1}H^{13}C$ HMBC NMR spectrum (500 MHz, DMSO-d_6) of **2d**.

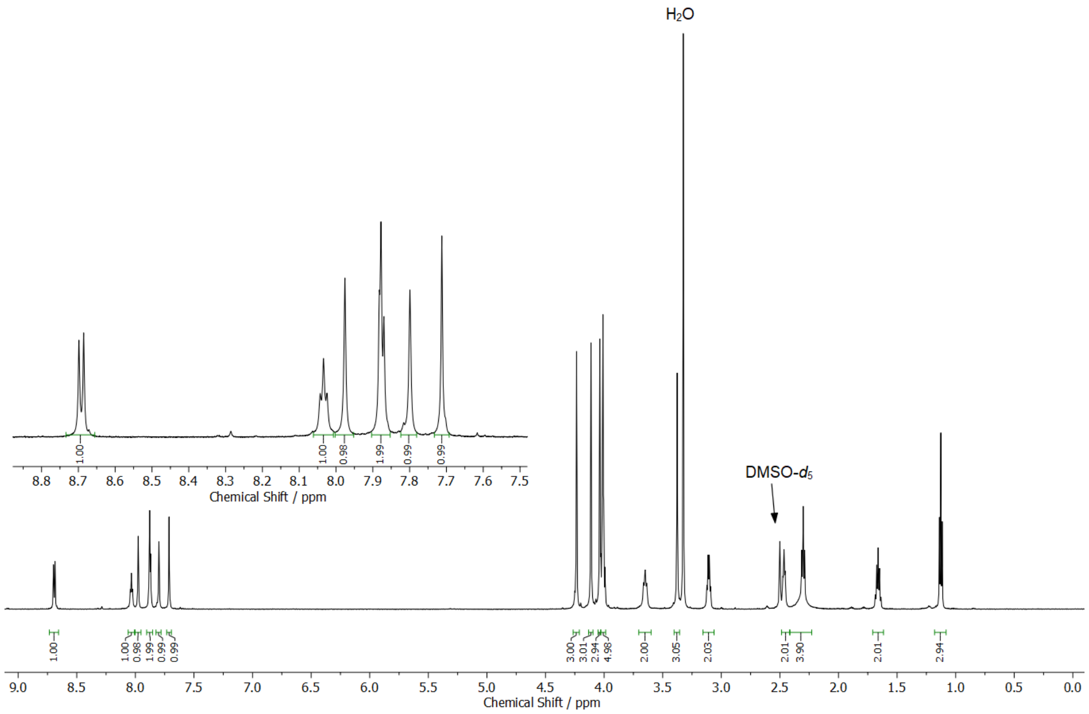

Figure S49. ^{1}H NMR spectrum (600 MHz, DMSO-d_6) of **2e**.

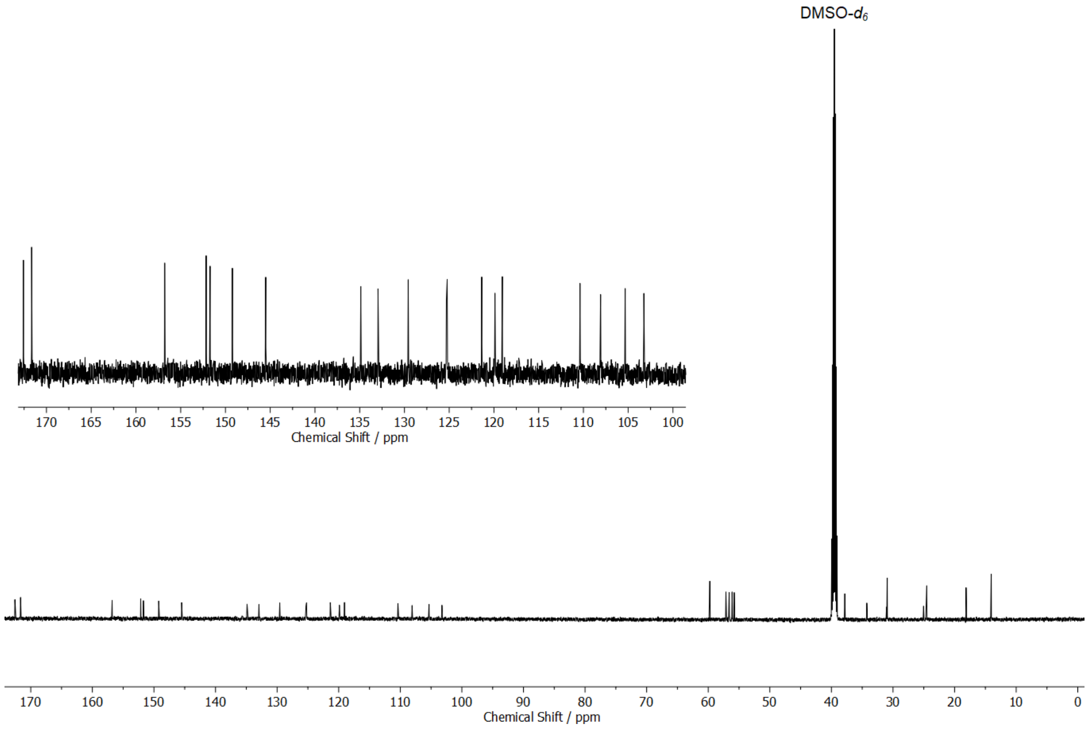

Figure S50. ^{13}C NMR spectrum (150 MHz, DMSO-d_6) of **2e**.

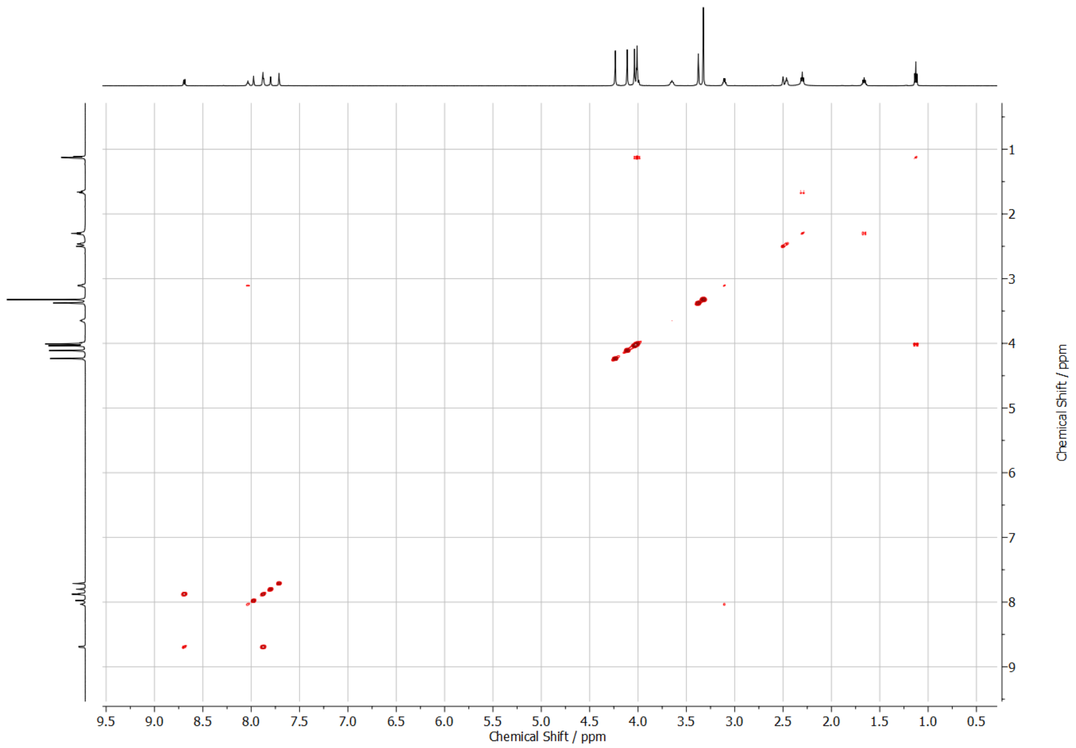

Figure S51. $^{1}H^{1}H$ COSY NMR spectrum (600 MHz, DMSO-d_6) of **2e**.

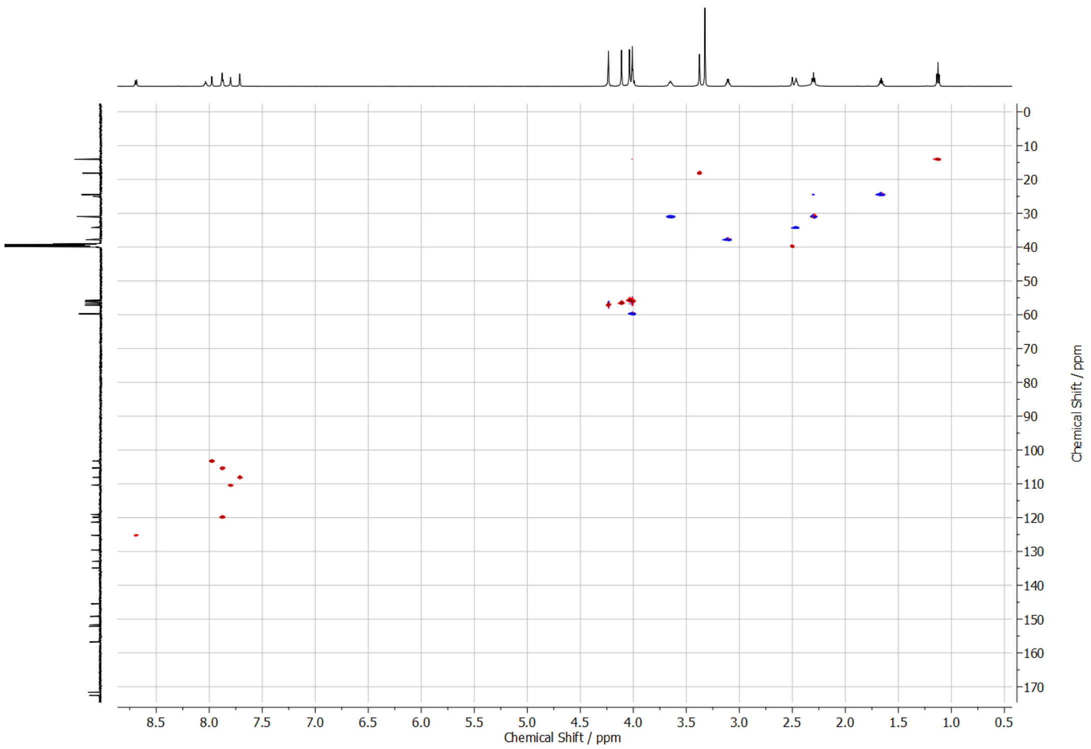

Figure S52. $^{1}H^{13}C$ HSQC NMR spectrum (600 MHz, DMSO-d_6) of **2e**.

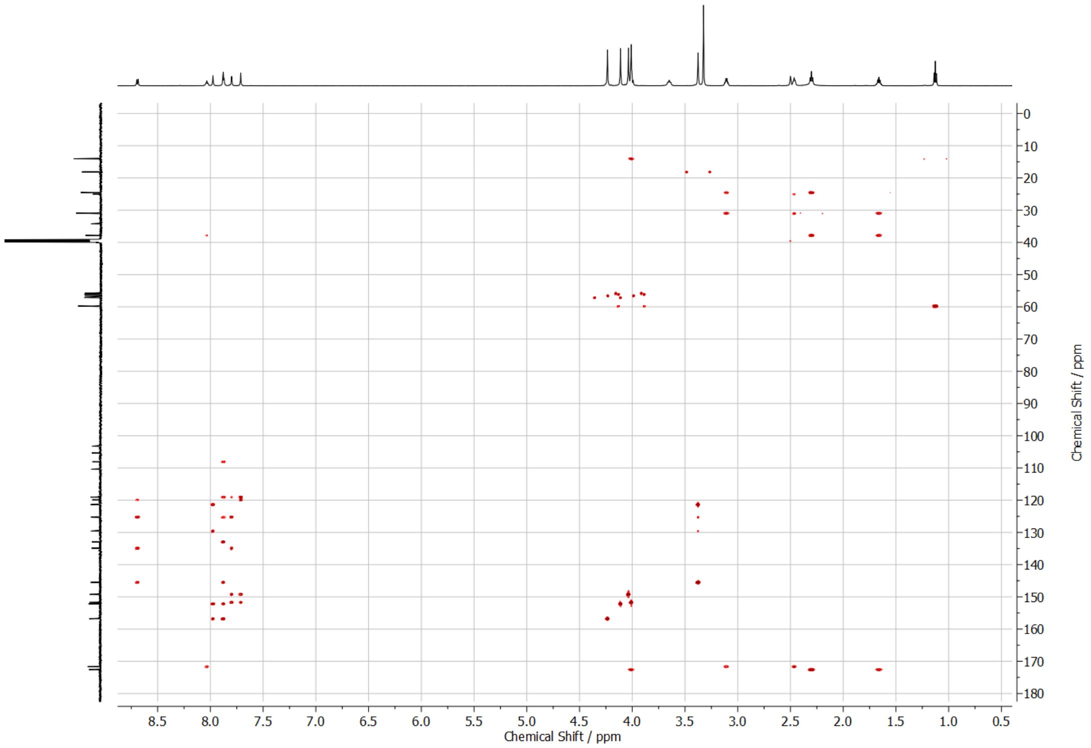

Figure S53. ^{1}H^{13}C HMBC NMR spectrum (600 MHz, DMSO-d_6) of **2e**.

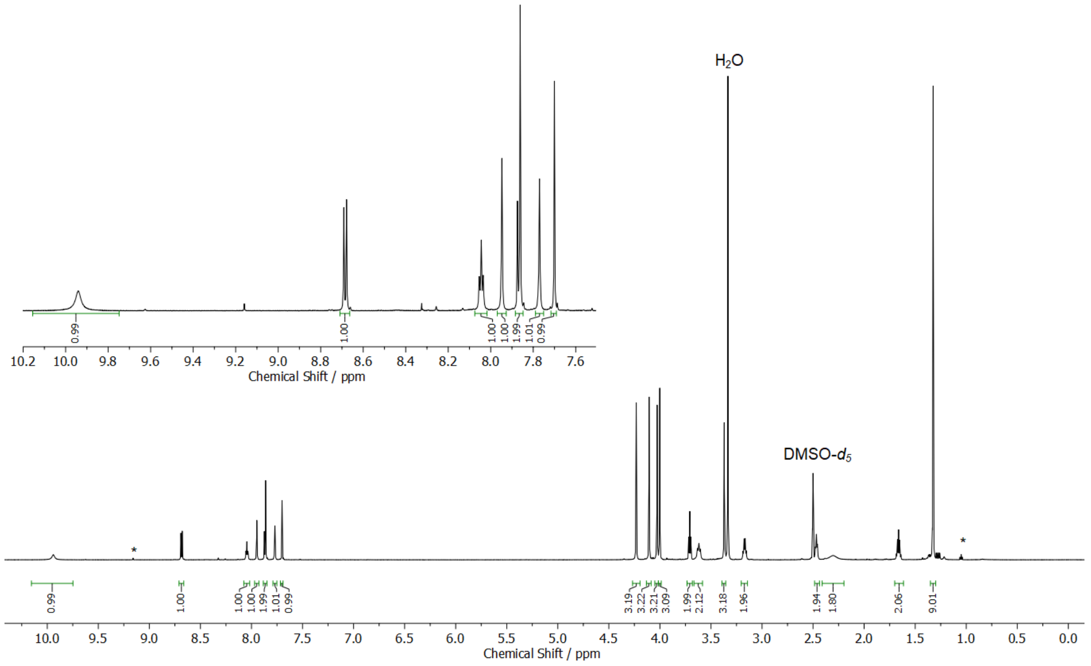

Figure S54. ^{1}H NMR spectrum (600 MHz, DMSO-d_6) of **2f** bromide salt.

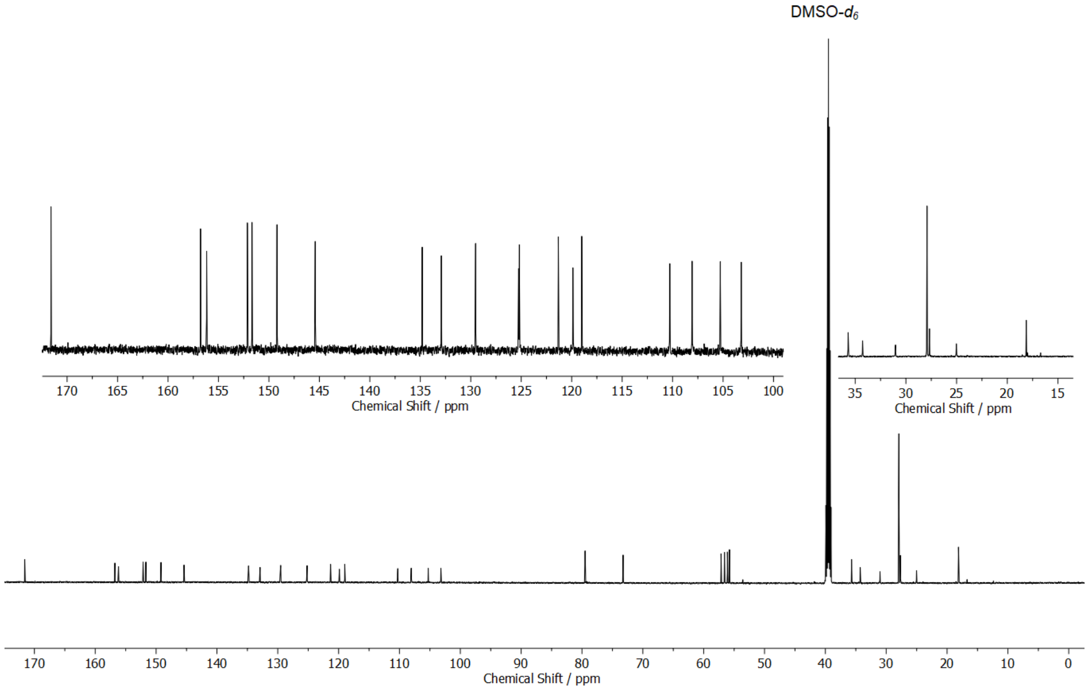

Figure S55. ^{13}C NMR spectrum (150 MHz, DMSO-d_6) of **2f** bromide salt.

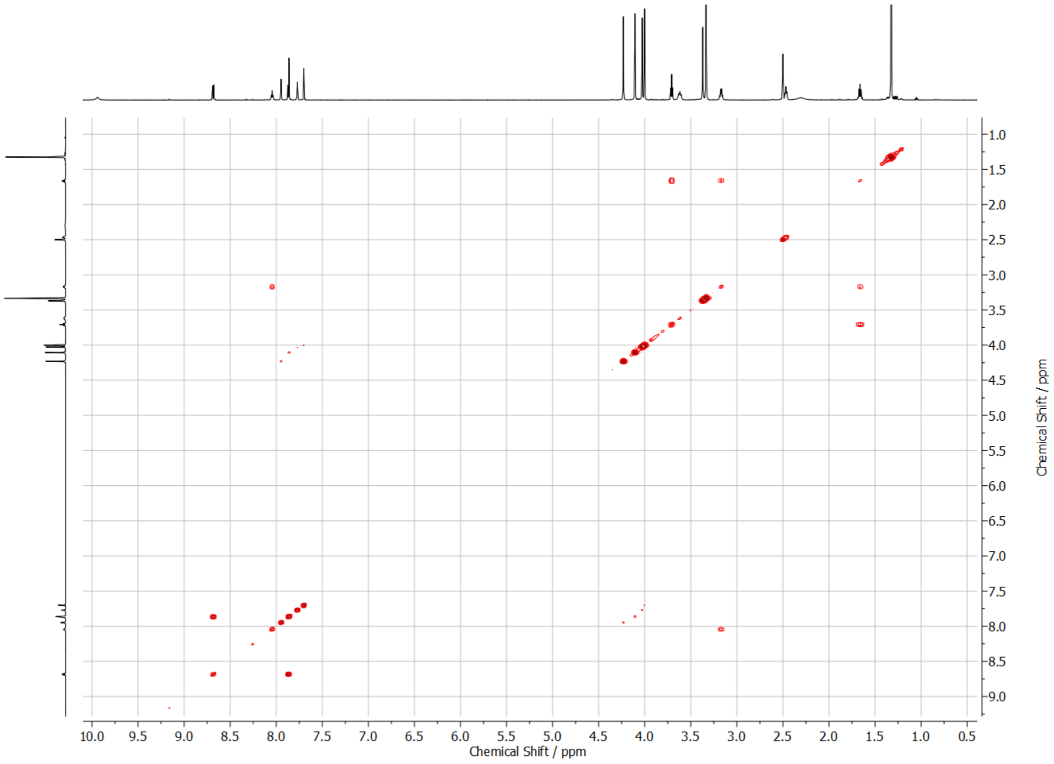

Figure S56. $^1H^1H$ COSY NMR spectrum (600 MHz, DMSO-d_6) of **2f** bromide salt.

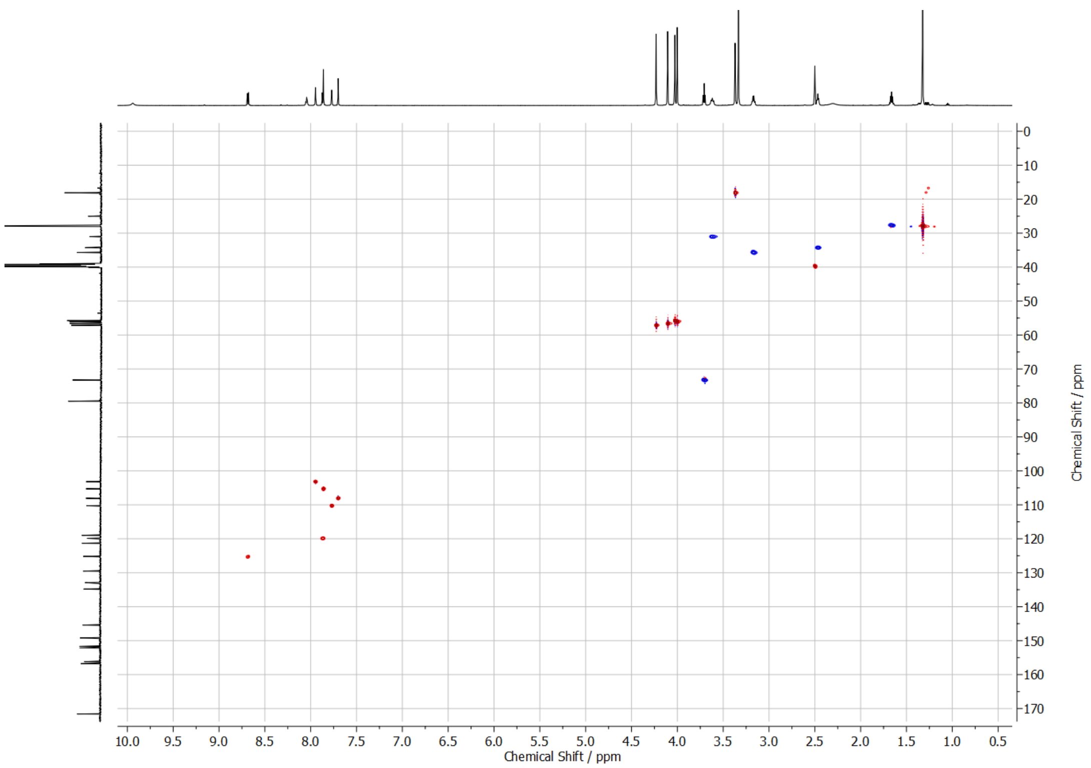

Figure S57. ^{1}H^{13}C HSQC NMR spectrum (600 MHz, DMSO-d_6) of **2f** bromide salt.

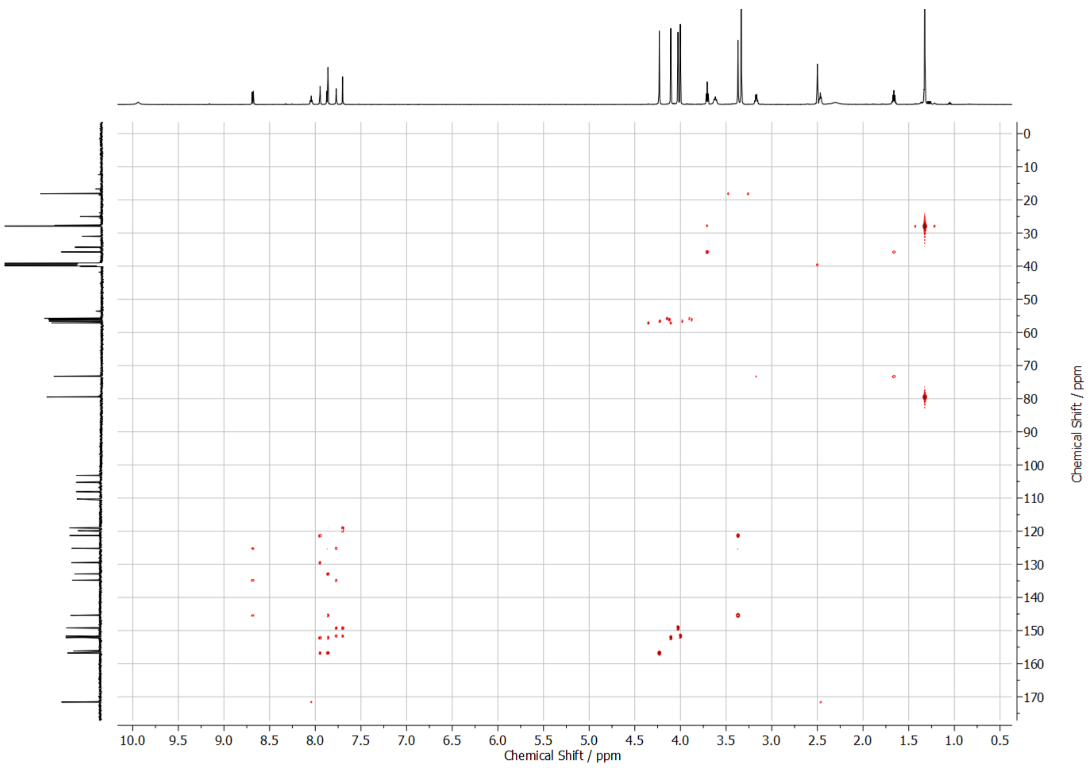

Figure S58. ^{1}H^{13}C HMBC NMR spectrum (600 MHz, DMSO-d_6) of **2f** bromide salt.

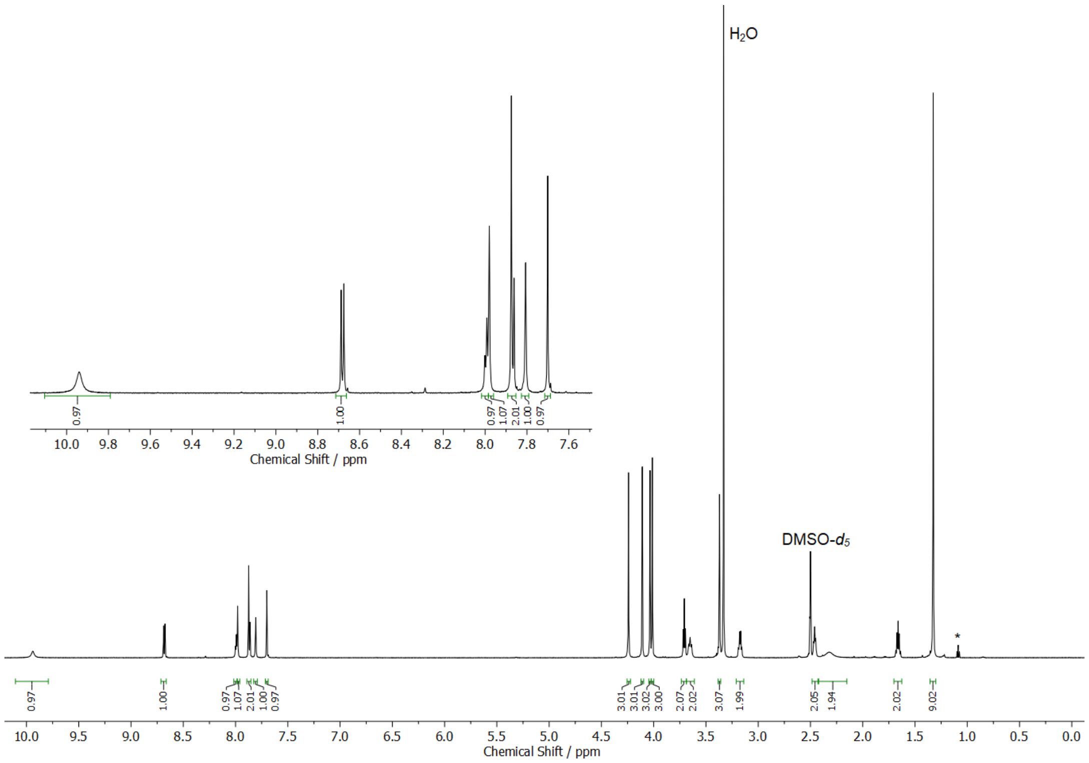

Figure S59. ^{1}H NMR spectrum (600 MHz, DMSO-d_6) of **2f**.

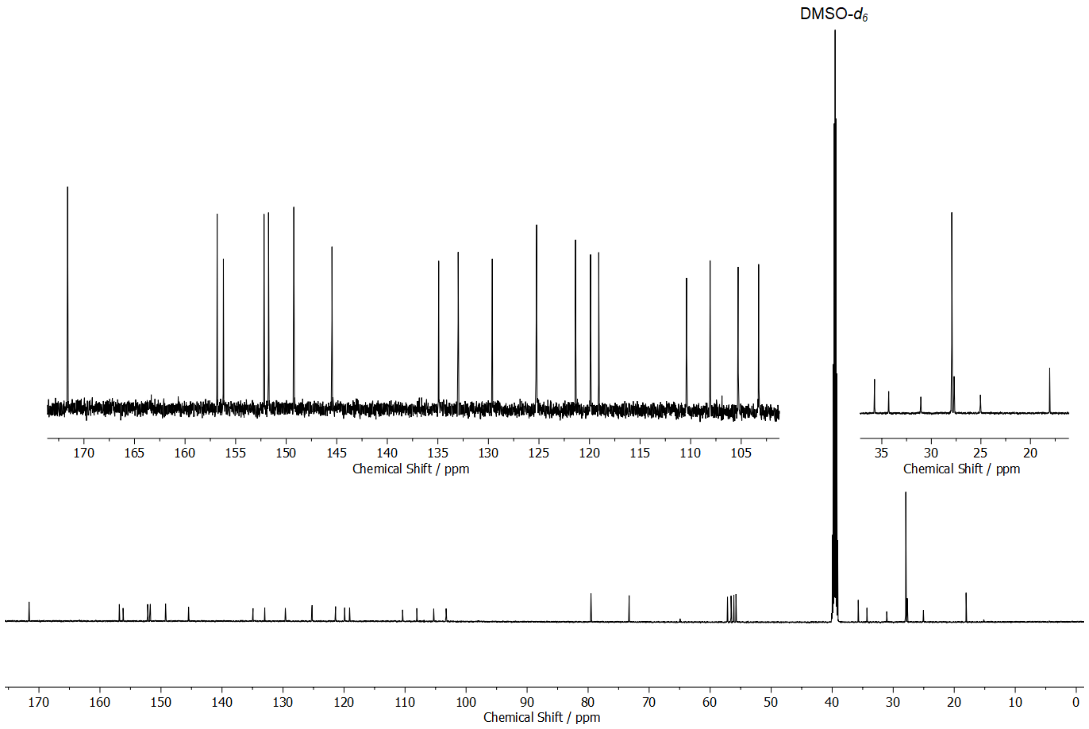

Figure S60. ^{13}C NMR spectrum (150 MHz, DMSO-d_6) of **2f**.

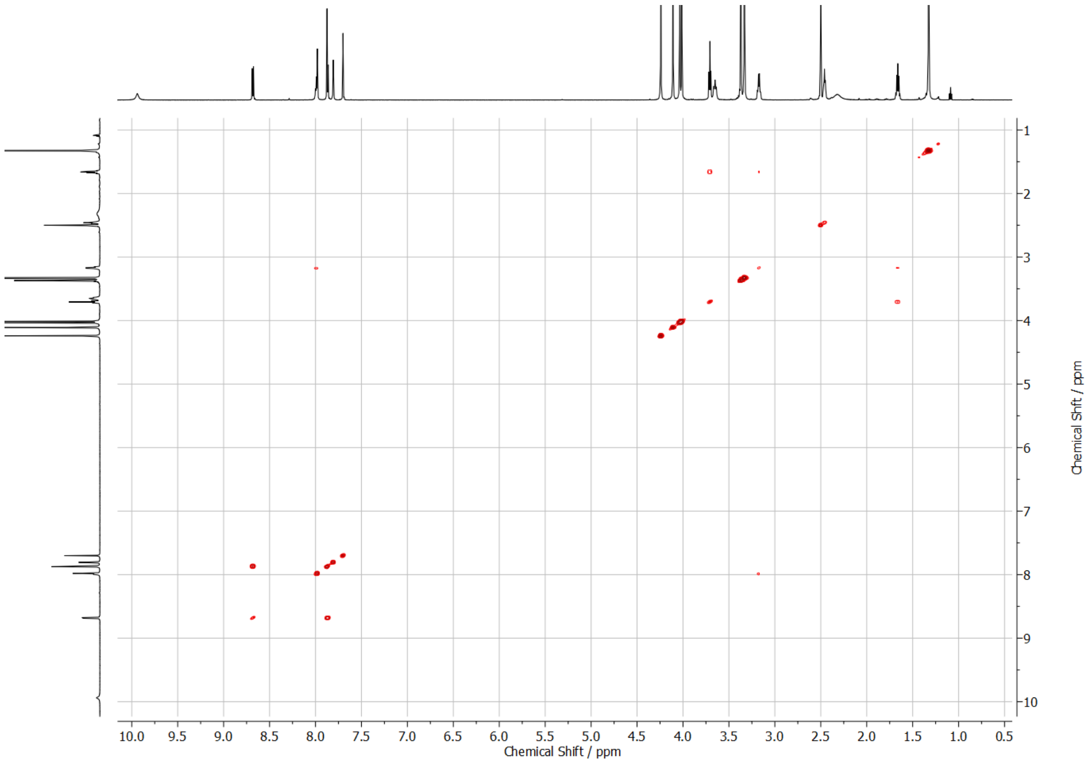

Figure S61. ^{1}H^{1}H COSY NMR spectrum (600 MHz, DMSO-d_6) of **2f**.

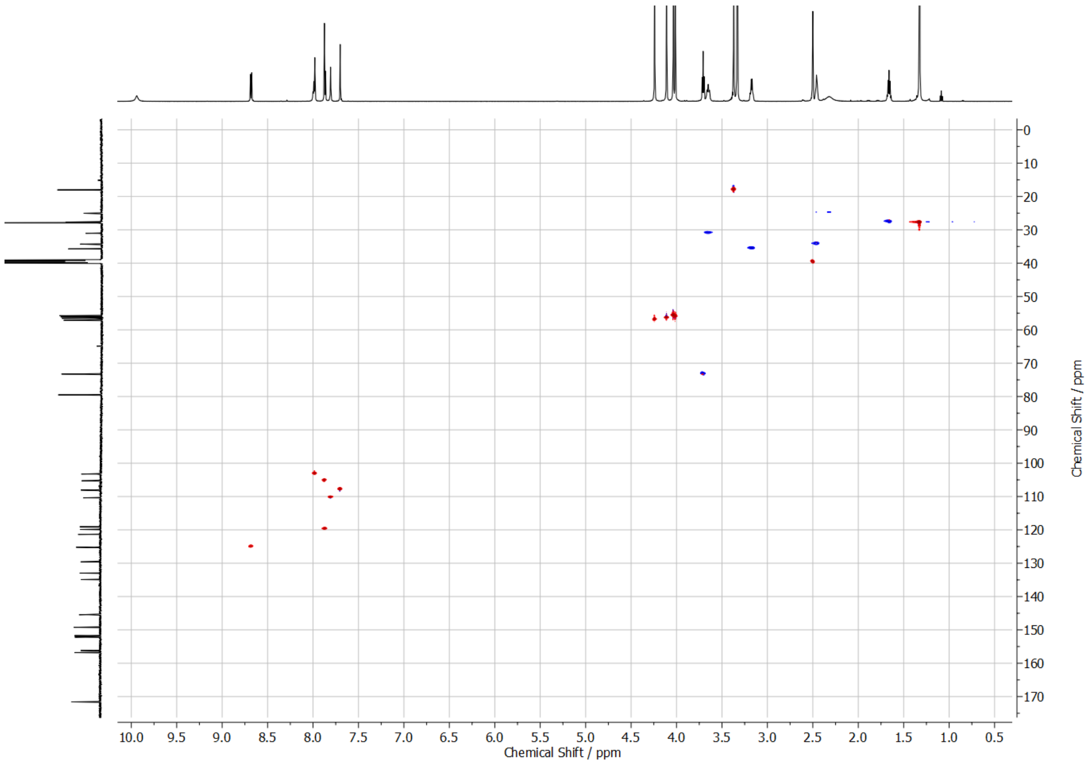

Figure S62. ^{1}H^{13}C HSQC NMR spectrum (600 MHz, DMSO-d_6) of **2f**.

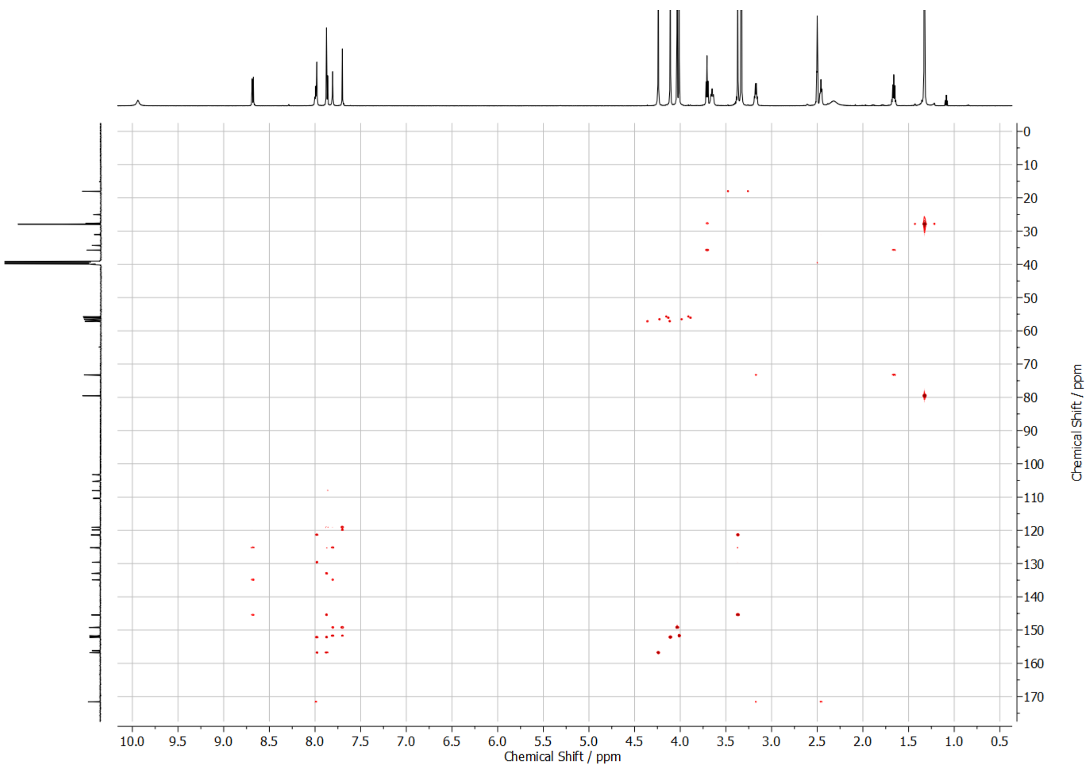

Figure S63. ^{1}H^{13}C HMBC NMR spectrum (600 MHz, DMSO-d_6) of **2f**.

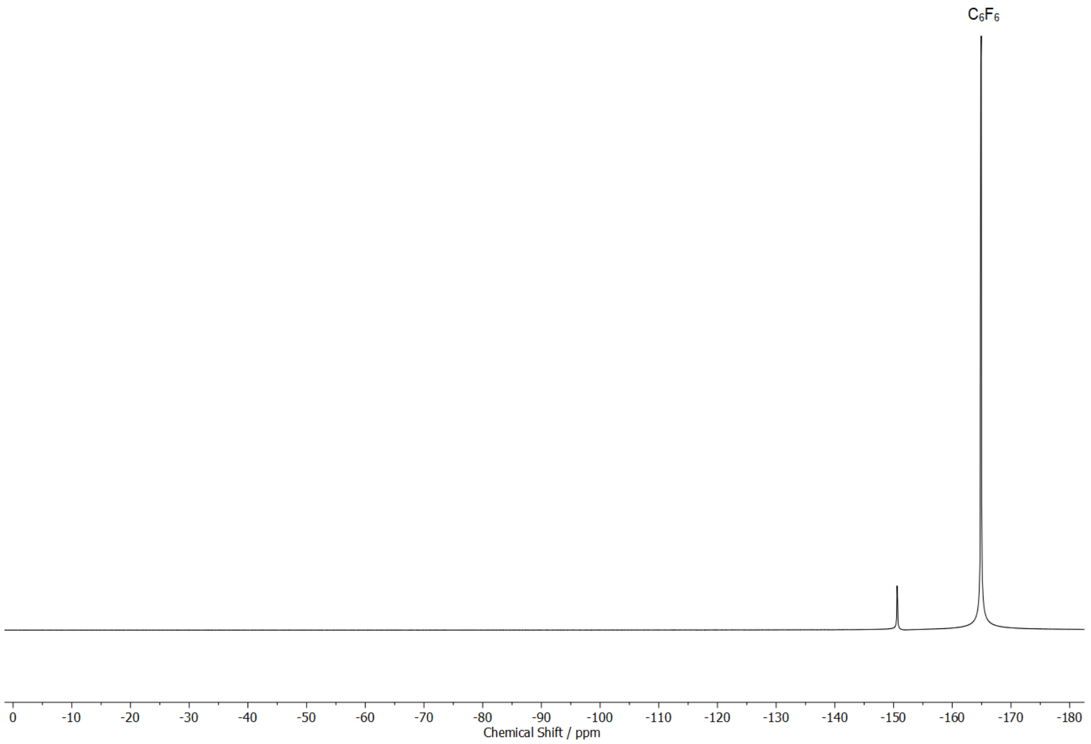

Figure S64. ^{19}F NMR spectrum (470 MHz, DMSO-d_6/C_6F_6) of **2f**.

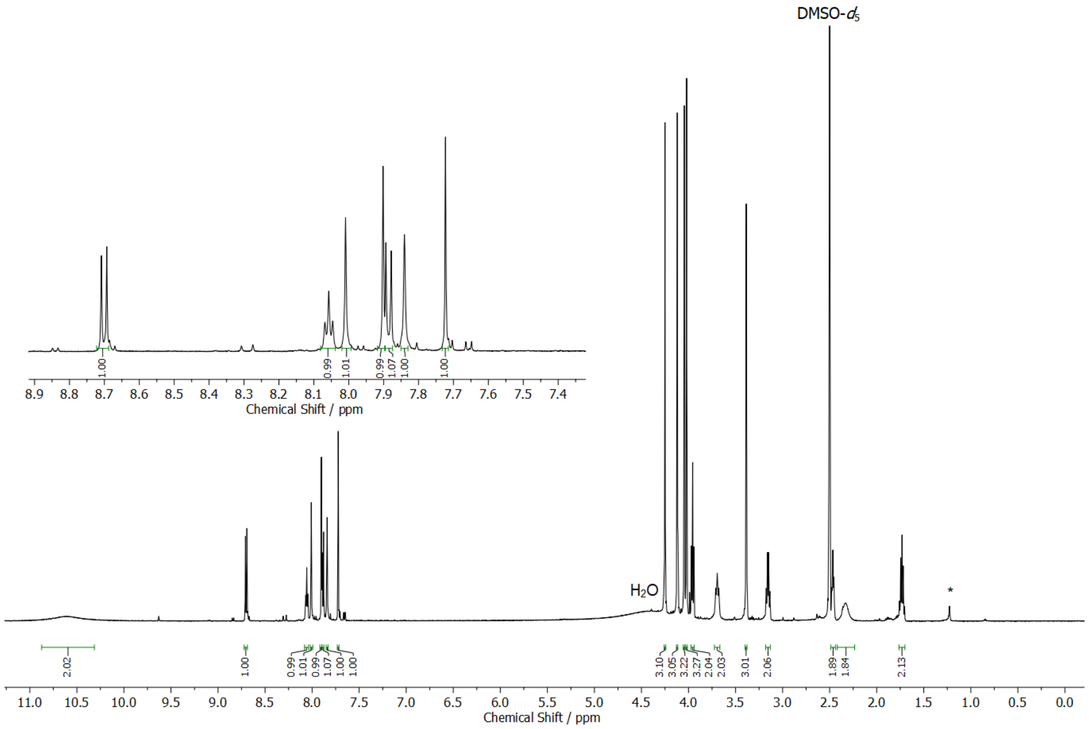

Figure S65. ^{1}H NMR spectrum (600 MHz, DMSO-d_6) of **2g**.

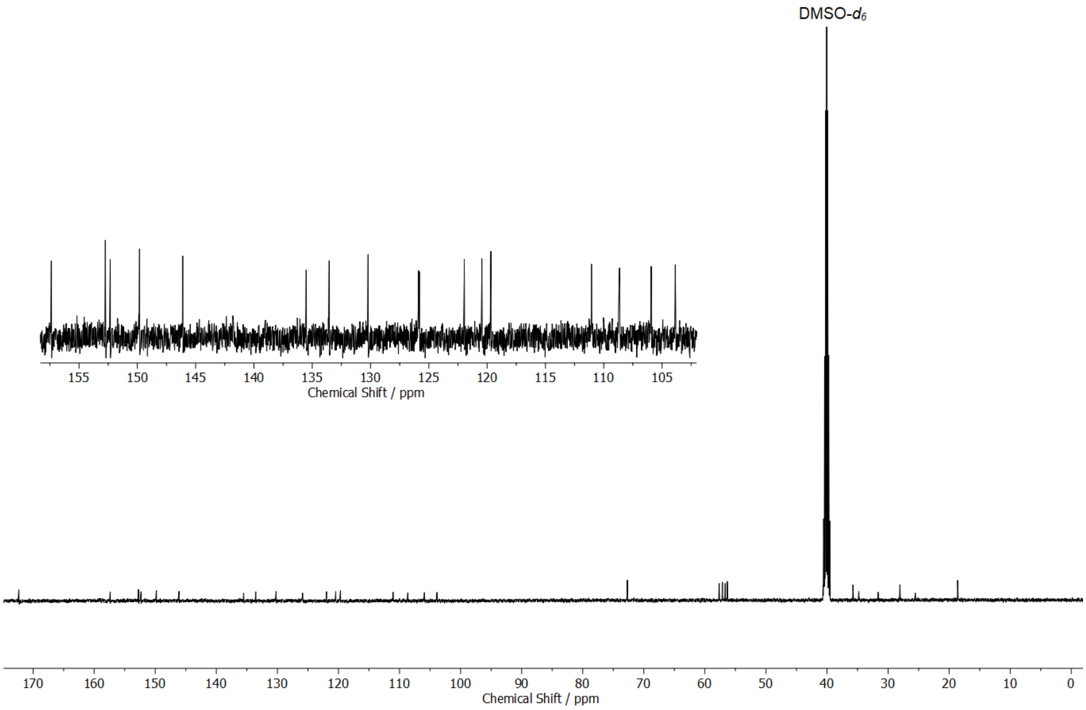

Figure S66. ^{13}C NMR spectrum (150 MHz, DMSO-d_6) of **2g**.

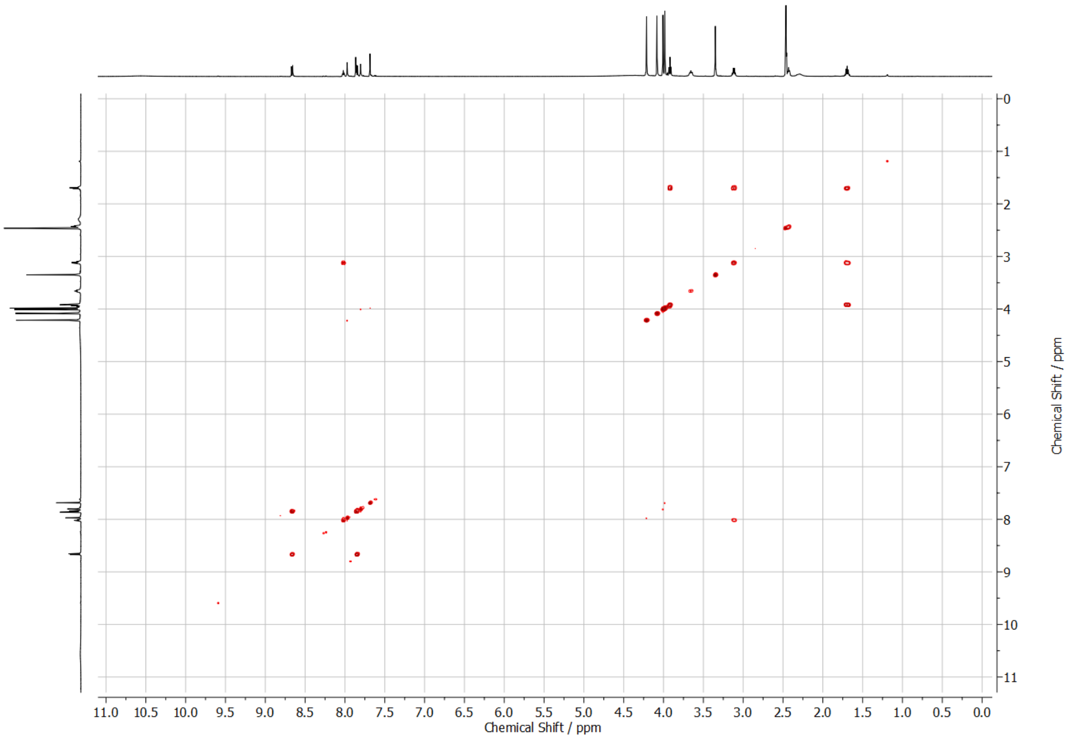

Figure S67. $^1H^1H$ COSY NMR spectrum (600 MHz, DMSO-d_6) of **2g**.

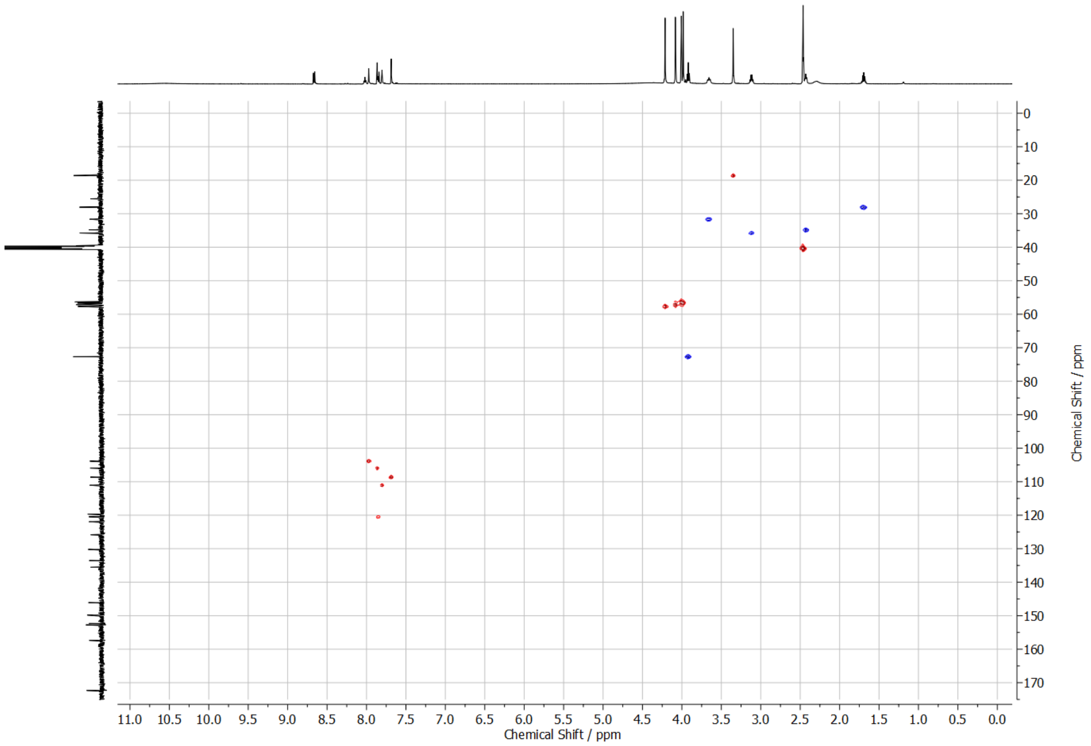

Figure S68. $^1H^{13}C$ HSQC NMR spectrum (600 MHz, DMSO-d_6) of **2g**.

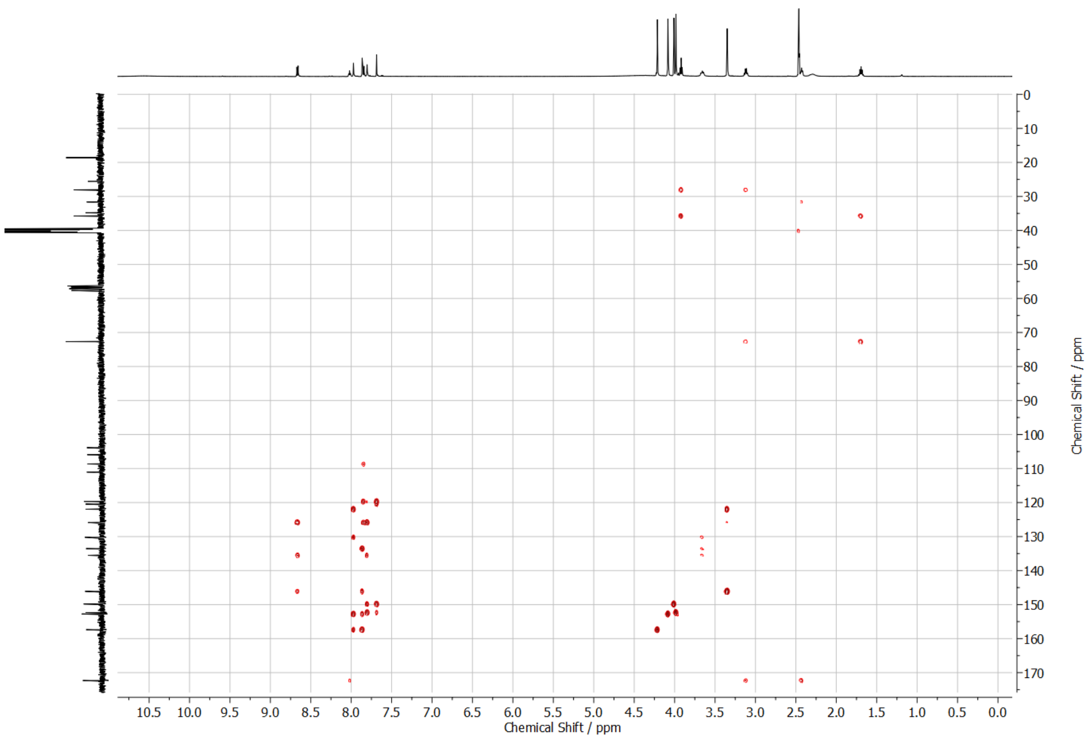

Figure S69. $^{1}H^{13}C$ HMBC NMR spectrum (600 MHz, DMSO-d_6) of **2g**.

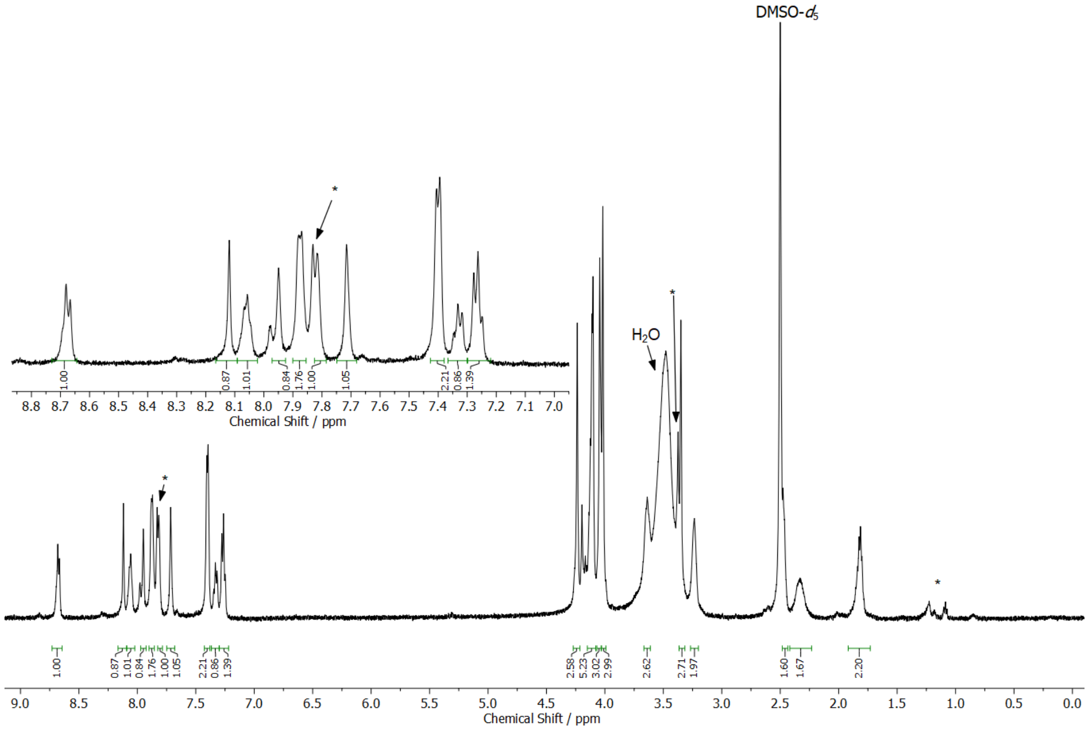

Figure S70. ^{1}H NMR spectrum (500 MHz, DMSO-d_6) of **2h**.

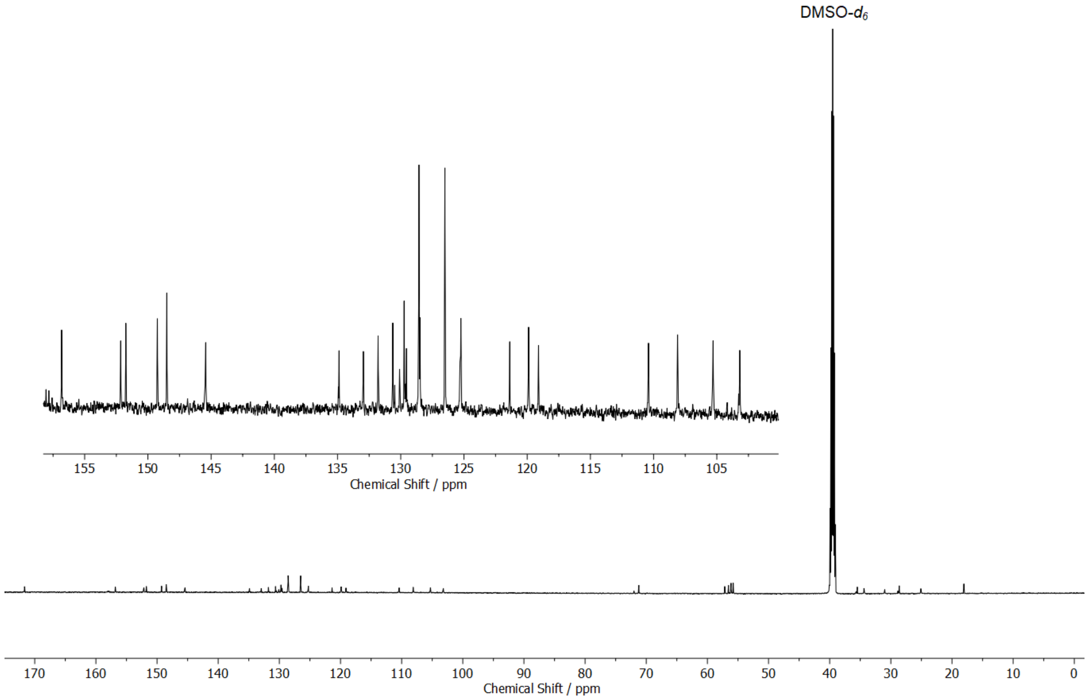

Figure S71. ^{13}C NMR spectrum (125 MHz, DMSO-d_6) of **2h**.

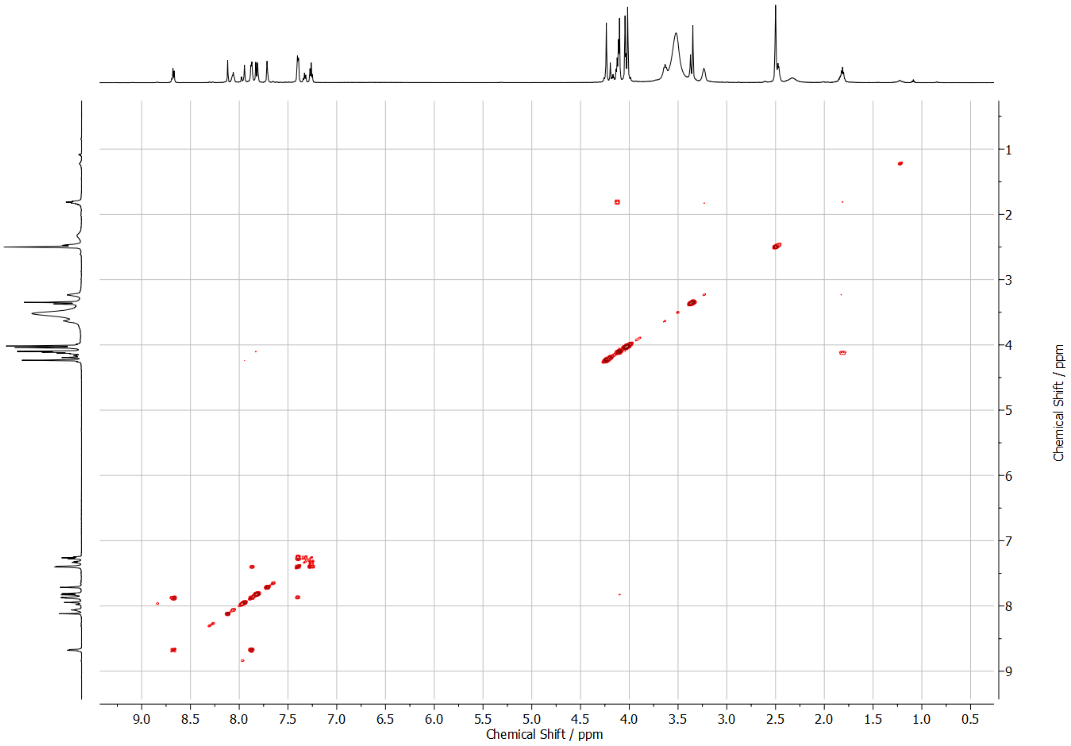

Figure S72. ^{1}H^{1}H COSY NMR spectrum (500 MHz, DMSO-d_6) of **2h**.

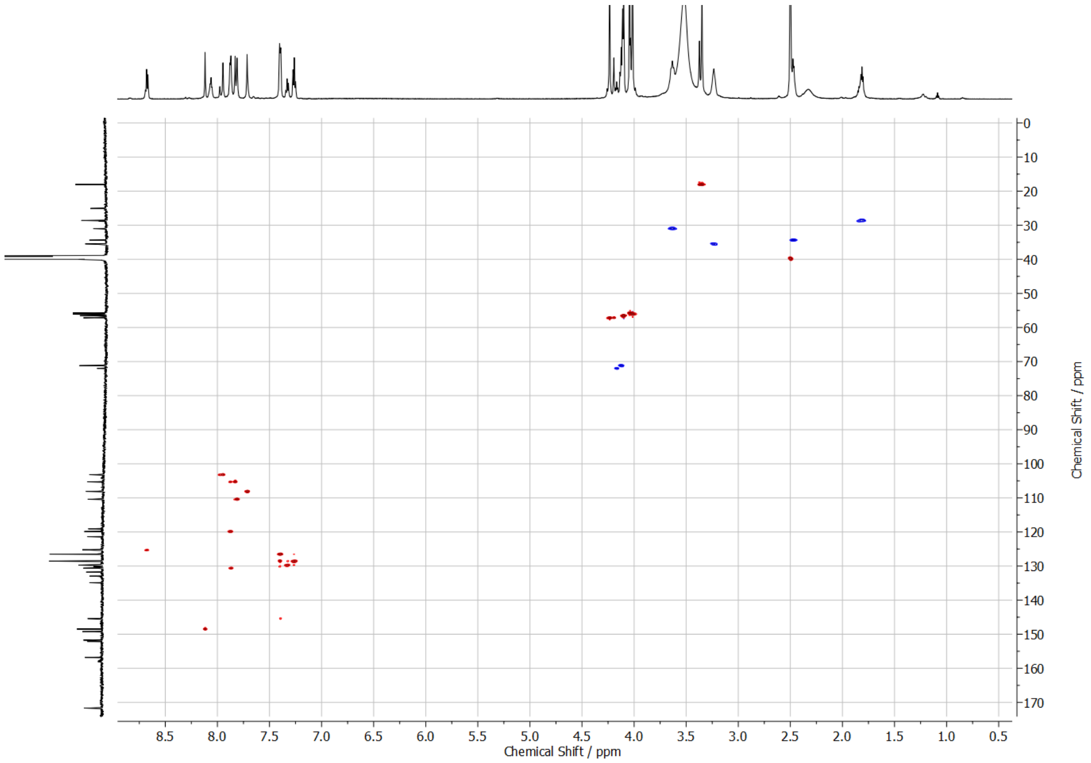

Figure S73. ^{1}H^{13}C HSQC NMR spectrum (500 MHz, DMSO-d_6) of **2h**.

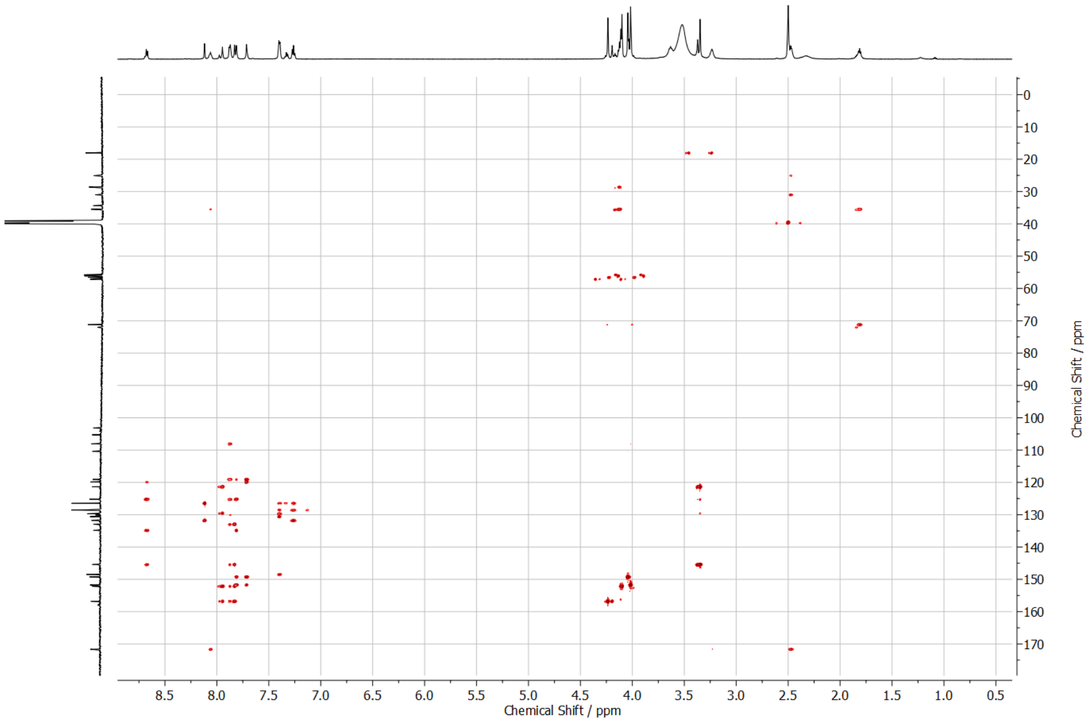

Figure S74. ^{1}H^{13}C HMBC NMR spectrum (500 MHz, DMSO-d_6) of **2h**.
